# Synthesis of imidazo[1,5-*a*]pyridines via cyclocondensation of 2-(aminomethyl)pyridines with electrophilically activated nitroalkanes

**DOI:** 10.3762/bjoc.16.239

**Published:** 2020-11-26

**Authors:** Dmitrii A Aksenov, Nikolai A Arutiunov, Vladimir V Maliuga, Alexander V Aksenov, Michael Rubin

**Affiliations:** 1 Department of Chemistry, North Caucasus Federal University, 1a Pushkin St., Stavropol 355017, Russian Federation; 2 Department of Chemistry, University of Kansas, 1567 Irving Hill Road, Lawrence, KS 66045, USA

**Keywords:** cyclization, heterocycles, imidazo[1,5-*a*]pyridines, nitroalkanes, polyphosphoric acid

## Abstract

Imidazo[1,5-*a*]pyridines were efficiently prepared via the cyclization of 2-picolylamines with nitroalkanes electrophilically activated in the presence of phosphorous acid in polyphosphoric acid (PPA) medium.

## Introduction

**Figure 1:**
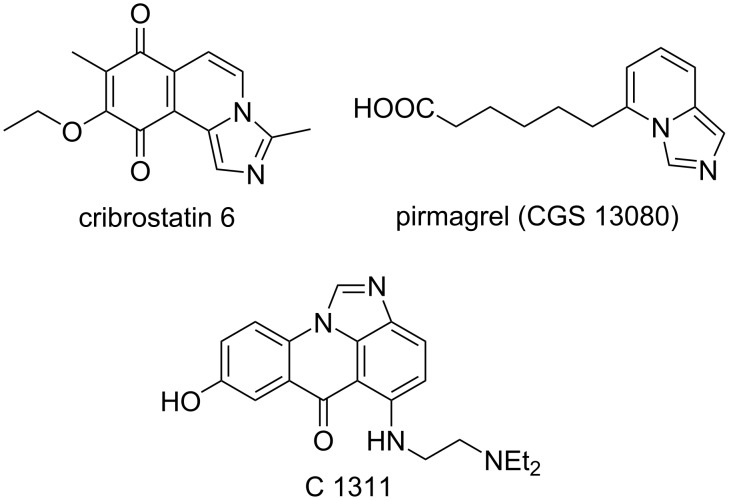
Biologically active imidazo[1,5-*a*]pyridines.

It is hard to overstate the importance of imidazo[1,5-*a*]pyridines in modern organic and medicinal chemistry. Several natural alkaloids possessing this core were isolated from marine sponges, for example, cribrostatin 6 (Figure 1) [1–3]. The imidazo[1,5-*a*]pyridine core is also considered to be one of the privileged pharmacophoric scaffolds and can be found in many biologically active compounds, for example, the potent antitumor agent C 1311 inhibiting topoisomerase II [4–9] or pirmagrel, a cytotoxic immunosuppressant and DNA synthesis inhibitor (Figure 1) [10–12]. In addition, compounds with this structure were investigated as photoluminescent sensors [13] and have been employed to generate pincer and heterocyclic carbene ligands for transition metal catalysis [14–15]. A lot of efforts have been dedicated to the development of efficient synthetic methods to access imidazo[1,5-*a*]pyridines, with more than 120,000 individual compounds prepared to date. Most synthetic approaches rely on various cyclocondensations of nucleophilic (2-aminomethyl)pyridine precursors, introducing a new five-membered ring. Typically, carboxylic acids [16–20], acyl anhydrides [21–23], acyl chlorides [24–27], esters [28], thioamides [29–31], dithionates [32–33], or thiocarbamates [34] are employed as electrophilic components, but oxidative cyclocondensations with aldehydes have also been showcased [35–37]. However, given the importance of these targets, new preparative methods are still in high demand. Herein we demonstrate a new synthetic approach towards imidazo[1,5-*a*]pyridines, taking advantage of the unusual electrophilic properties of nitroalkanes activated by PPA.

## Results and Discussion

**Scheme 1:**
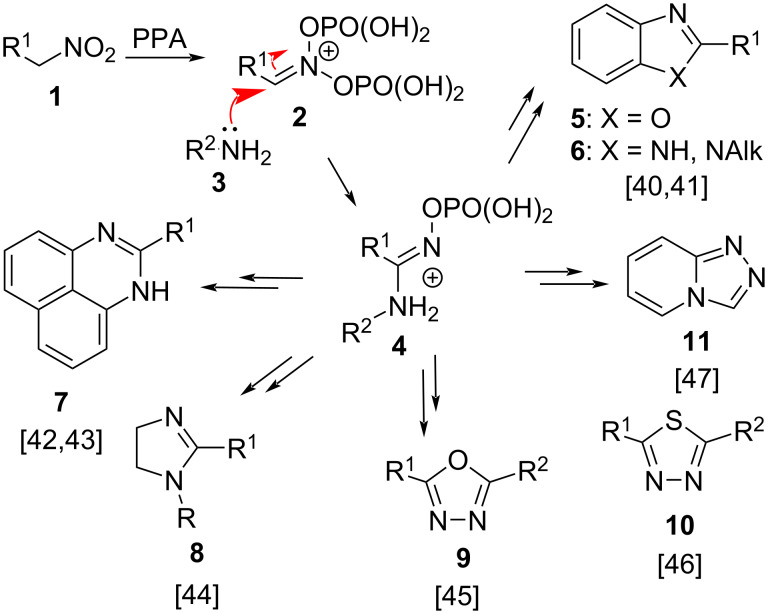
Activation of nitroalkanes towards nucleophilic attack by amines.

Within the last few years, we have developed and implemented a number of novel synthetic methodologies in our laboratories, involving acid-mediated cascade transformations of nitroalkenes and nitroalkanes, which target materials science and medicinal chemistry applications. It was demonstrated that upon heating in polyphosphoric acid, the nitroalkanes **1** convert into the phosphorylated nitronates **2**, which exhibit strong electrophilic properties. This allowed for the employment of these species in reactions wherein electron-rich arenes serve as carbon-based nucleophilies [38–40]. It was also discovered that the nucleophilic amines **3** can be successfully employed in this type of transformations as well, providing the amidinium intermediates **4**, which are susceptible to a variety of subsequent cyclizations. This approach opens up a novel avenue by which to access the benzoxazoles **5** [40], benzimidazoles **6** [40–41], diazines **7** [42–43], or imidazolines **8** (Scheme 1) [44]. We have also shown that a nucleophilic attack on the phosphorylated nitronate species **2** can be carried out with the participation of *N*-acylhydrazides or thiosemicarbazides to afford the 1,3,4-oxadiazoles **9** [45] and the 1,3,4-thiadiazoles **10** [46], respectively (Scheme 1). Finally, it was found that the reaction of 2-hydrazinylpyridine with electrophilically activated nitroalkanes provides the corresponding triazolopyridines **11** (Scheme 1) [47].

**Scheme 2:**
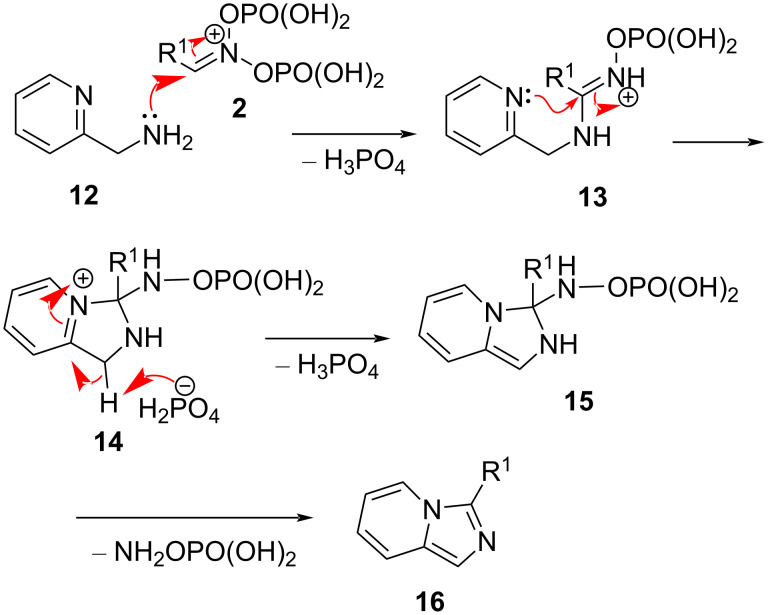
Mechanistic rationale.

These results prompted the desire to implement this general scheme in order to access highly important imidazo[1,5-*a*]pyridines. It was imagined that the initial nucleophilic attack of the 2-(aminomethyl)pyridine (**12**) on the nitronate **2** would provide an amidinium species **13**, which is very well suited for a 5-*exo*-trig cyclization involving the masked imine moiety of the pyridine ring, providing a 2,3-dihydro-1*H*-imidazo[1,5-*a*]pyridin-4-ium ion **14**. After deprotonation, the latter would form the 2,3-dihydroimidazo[1,5-*a*]pyridine **15**, which after the elimination of *O*-phosphorylated hydroxylamine, would afford the imidazo[1,5-*a*]pyridines **16** (Scheme 2).

**Scheme 3:**
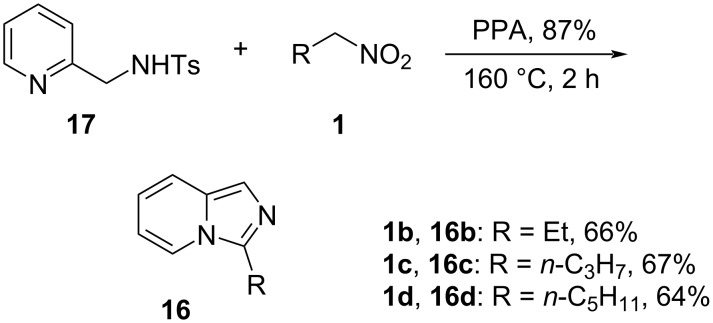
Reaction of the *N*-tosylate **17** with electrophilic nitroalkanes.

To test this idea, nitroethane (**1а**, 5 equiv) was heated at 110 °C with 85% PPA for 30 min, and 2-(aminomethyl)pyridine (**12**) was added in small portions. After 3 h of stirring at 110 °C, the reaction mixture was quenched with ice-cold water, neutralized with aqueous ammonia, and extracted with ethyl acetate. The organic layer was analyzed by GC and ^1^H NMR to reveal that the desired product **16a** was formed in a disappointingly low yield (Table 1, entry 1). Heating of the reaction mixture to 130 °C, together with the employment of even more highly concentrated 87% PPA, resulted in an improved conversion, but only insignificantly so (Table 1, entries 2 and 3). To the contrary, an attempt to carry out the reaction in 80% PPA provided a conversion of only 6% even at 140 °C (Table 1, entry 4), whereas no reaction was observed in 100% orthophosphoric acid (Table 1, entry 5). Evidently, under acidic conditions, protonation of the primary amino group in **12** attenuates the nucleophilicity of the molecule, rendering the attack on the relatively bulky phosphorylated nitronate **2** inefficient. To prove this, we decided to moderate the basicity of the amine function by protection with a sulfonyl group. Gratifyingly, the *N*-tosylamine **17** [48] reacted smoothly with 1-nitropropane (**1b**), 1-nitrobutane (**1c**), and 1-nitrohexane (**1d**) in the presence of 87% PPA, although this required heating to 160 °C to bring the reaction to completion. The corresponding imidazo[1,5-*a*]pyridines **16b**–**d** were isolated in good practical yield in all three cases (Scheme 3).

**Table 1:** Optimization of the reaction conditions for the cyclization of 2-(aminomethyl)pyridine (**12**) with nitroethane (**1a**).

entry	medium^a^	*T*, °C (*t*, h)	yield, %^b^

1	PPA 85% 1 g/mmol	110 (3)	4
2	PPA 85% 1 g/mmol	130 (3)	13
3	PPA 87% 1 g/mmol	130 (3)	15
4	PPA 80%	140 (3)	6
5	H_3_PO_4_ 100%	140 (5)	0
6	PPA 87% 0.5 g/H_3_PO_3_ 0.25 g/mmol	110 (5)	22
7	PPA 87% 0.5 g/H_3_PO_3_ 0.25 g/mmol	140 (2)	43
8	PPA 87% 0.5 g/H_3_PO_3_ 0.5 g/mmol	140 (1.5)	62
9	PPA 87% 0.5 g/H_3_PO_3_ 0.5 g/mmol	160 (2)	77^c^

^a^Amounts are provided in grams per mmol of substrate **12**. ^b^NMR yields, unless specified otherwise. ^c^Isolated yield of purified material.

This approach, however, seems less attractive as it requires an additional step for the proper modification of the starting material. An alternative, more appealing method would capitalize on the in situ generation of a less sterically hindered, and therefore more reactive electrophilic nitronate. As we have recently demonstrated, this type of species can be efficiently generated upon the treatment of nitroalkanes with anhydrous H_3_PO_4_ or in combination with 87% PPA [44]. We were pleased to find that the performance of the direct transformation of **12** into **16a** was significantly improved after doping the PPA medium with H_3_PO_3_. Indeed, in a mixture of 87% PPA and H_3_PO_3_ 2:1 (mass/mass ratio), the yield reached 22% and 43% at 110 °C and 140 °C, respectively (Table 1, entries 6 and 7). A further increase of the H_3_PO_3_ concentration and temperature improved the result. In the 1:1 87% PPA/H_3_PO_3_ mixture at 140 °C, the yield of **16a** improved to 62% (Table 1, entry 8), while at 160 °C, it reached 77% (Table 1, entry 9). It should be pointed out that the reported yield of 77% is the isolated yield given complete conversion, with no detected side products aside from easily removable polymeric resins.

**Scheme 4:**
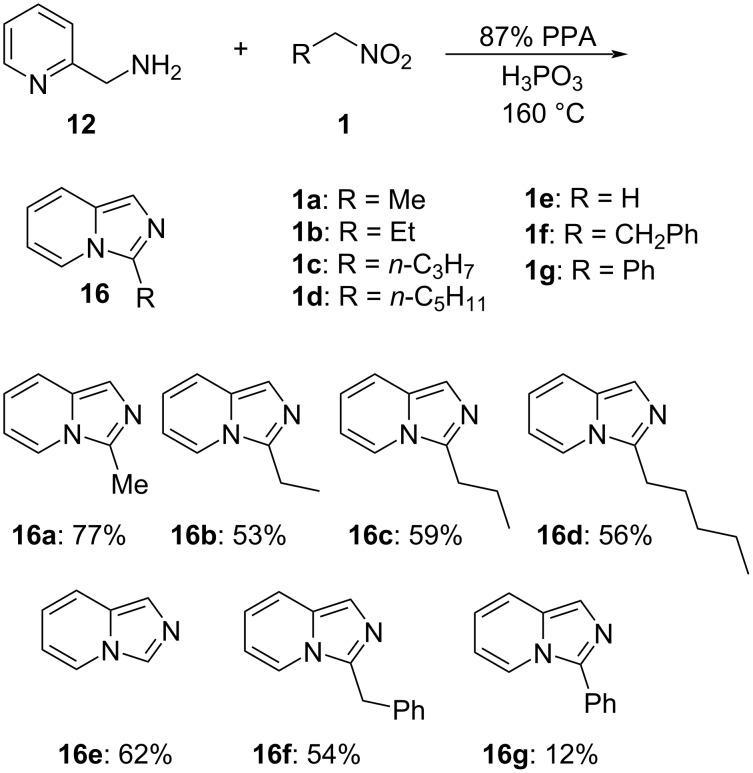
Reaction of 2-(aminomethyl)pyridine (**12**) with electrophilic nitroalkanes.

With optimized reaction conditions in hand, we proceeded with the examination of the scope of, and tolerance towards the differrent nitroalkanes **1b**–**f**. Overall, the reaction proceeded smoothly, providing moderate yields of the corresponding imidazo[1,5-*a*]pyridines **16b**–**f** (Scheme 4). The only exception to this general trend was the reaction with α-nitrotoluene (**1g**), which proceeded sluggishly and provided a disappointingly low yield of 3-phenylimidazo[1,5-*a*]pyridine (**16g**, Scheme 4).

**Scheme 5:**
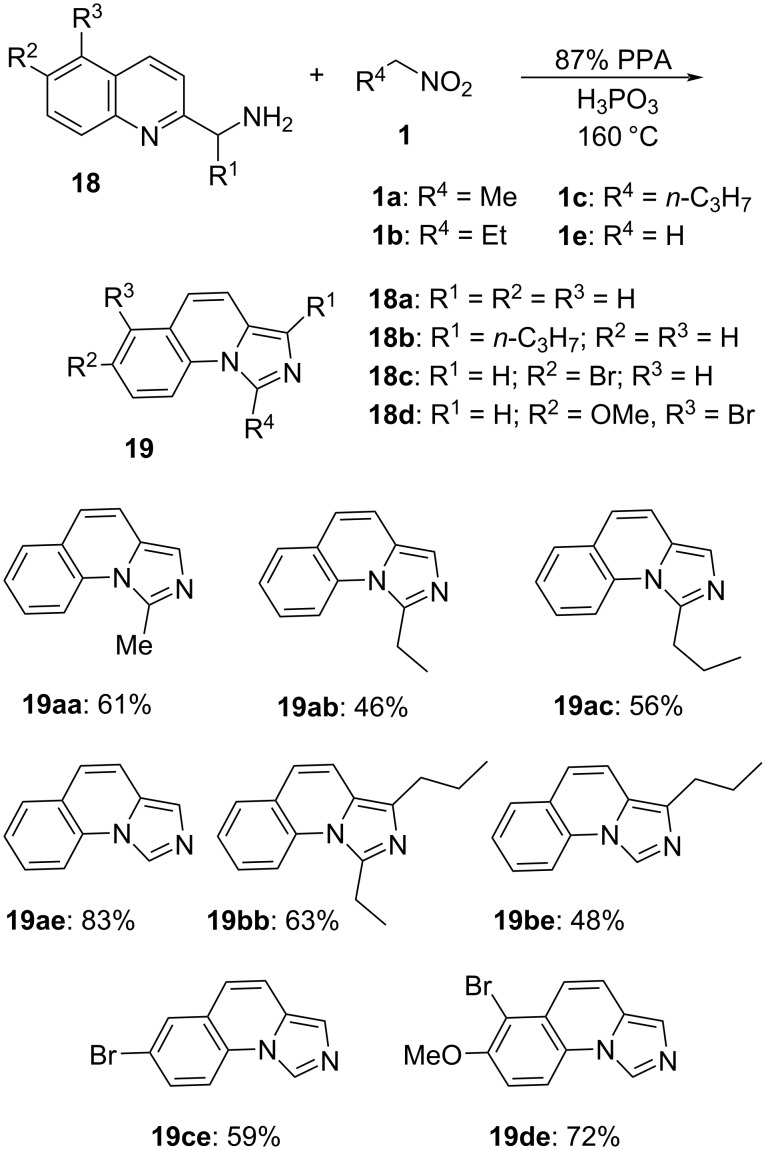
Reaction of the 2-(aminomethyl)quinolines **18** with electrophilic nitroalkanes.

Next, the scope of nucleophilic substrates was investigated. The readily available 2-(aminomethyl)quinolines **18** bearing various substituents at C-5, C-6, and C-1' were tested in reactions with the nitroalkanes **1a**–**c** and **1e**. All of these reactions proceeded uneventfully, affording the corresponding imidazo[1,5-*a*]pyridines in moderate to good yield (Scheme 5).

**Scheme 6:**
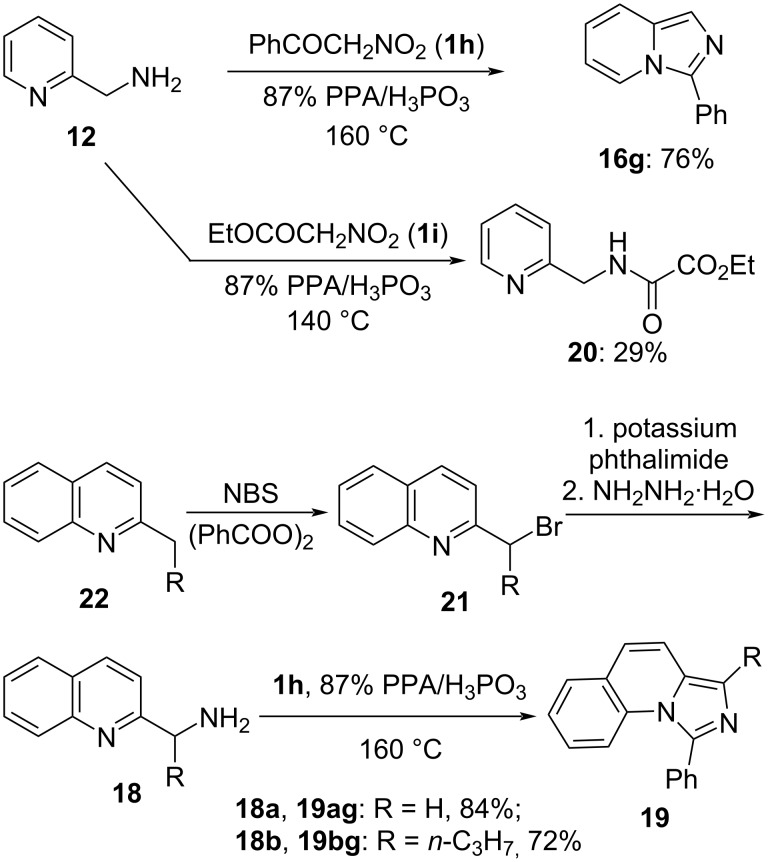
Reactivity of α-nitroacetophenone (**1h**) and α-nitroacetic ester (**1i**).

We also attempted to address the issue of the low reactivity of α-nitrotoluene (**1g**). We have previously demonstrated that in the reaction with nucleophilic 1,2-diamines, α-nitroacetophenone (**1h**) can be employed as a much more versatile synthetic equivalent to afford the corresponding 2-phenylimidazolines [44]. Gratifyingly, this approach also worked well with the substrates **12**, **18a**, and **18b**. The corresponding imidazo[1,5-*a*]pyridines **16g**, **19ag**, and **18bg** were obtained in a highly efficient manner (Scheme 6). In addition, α-nitroacetic ester (**1i**) was probed as an electrophile in the reaction with substrate **12** in order to demonstrate that the phosphorylated nitronate function is a superior electrophile in comparison to the ester function. Indeed, this bielectrophilic reagent reacted only at the nitro group, providing the amide **20** as the sole product. The yield was quite marginal, primarily due to decomposition of the fragile ester functionality under the harsh reaction conditions employed. Unfortunately, cyclization did not take place (Scheme 6).

## Conclusion

In conclusion, a new method to access imidazo[1,5-*a*]pyridines via the unusual cyclization of 2-(aminomethyl)pyridines or 2-(aminomethyl)quinolines with nitroalkanes that are electrophilically activated in the presence of phosphorous acid in polyphosphoric acid medium was described. The reaction is quite sensitive to steric factors and affords medium to good yields of the products, although relatively harsh reaction conditions are required. The introduction of a phenyl substituent proved problematic in the reactions involving α-nitrotoluene, but this could be easily circumvented by replacing it with the better-performing α-nitroacetophenone (**1h**).

## Experimental

**General information**. ^1^H and ^13^C NMR spectra were recorded on a Bruker Avance-III spectrometer (400 or 100 MHz, respectively) equipped with a BBO probe in CDCl_3_ or DMSO-*d*_6_, using TMS as an internal standard. High-resolution mass spectra were obtained using a Bruker Maxis spectrometer (electrospray ionization, MeCN solution, using HCO_2_Na/HCO_2_H for calibration). Melting points were measured with a Stuart smp30 apparatus. All reactions were performed in oven-dried flasks equipped with reflux condensers and magnetic stir bars. All reactions were followed by thin-layer chromatography (TLC) using Silufol UV-254 plates, which were visualized under UV light (254 nm), with acetone, hexane/acetone, or hexane/ethanol/acetone mixtures as eluent. Polyphosphoric acid (87%) was obtained by dissolving a precise amount of P_2_O_5_ in 85% orthophosphoric acid according to the published protocols [49–50]. All other reagents and solvents were purchased from commercial vendors and used as received.

### 1-(6-Bromoquinolin-2-yl)methanamine (**18c**): typical procedure 1

First, 6-bromo-2-(bromomethyl)quinoline (**21c**) was prepared from commercially available 6-bromo-2-methylquinoline (**22c**, via radical bromination in the presence of NBS and dibenzoyl peroxide [51]. To this end, the methylquinoline **22c** (3.33 g, 15 mmol) was dissolved in carbon tetrachloride (30 mL), and *N*-bromosuccinimide (2.94 g, 16.5 mmol) was added, followed by dibenzoyl peroxide (194 mg, 0.80 mmol). The mixture was refluxed for 5–7 h while the reaction progress was monitored by TLC. Upon completion, the reaction mixture was cooled to room temperature, the formed precipitate of succinimide was filtered off, and the filtrate was concentrated in vacuum. The crude product was purified by flash column chromatography eluting with EtOAc/petroleum ether 1:6. Then, the amine was afforded via a Gabriel synthesis using a modified protocol described in the literature [52]. To a stirred solution of 6-bromo-2-(bromomethyl)quinoline (3.01 g, 10 mmol) in DMF (16 mL) was added potassium phthalimide (1.85 g, 10 mmol) in portions. The mixture was stirred at room temperature for 5 h. Then, water (30 mL) was added, and the formed precipitate was collected by filtration and washed with water. Recrystallization from ethanol afforded colorless crystals of intermediate 2-((6-bromoquinolin-2-yl)methyl)isoindoline-1,3-dione. This material (3.05 g, 8.3 mmol) was suspended in ethanol, and hydrazine hydrate (50%, 1.0 g, 10 mmol) was added. The reaction mixture was refluxed for 5 h and then cooled down to 0 °C. The precipitated phthalyl hydrazide was filtered off and rinsed several times with cold ethanol. The combined filtrates were concentrated under reduced pressure to provide the crude amine, which was purified by flash column chromatography on silica gel, eluting with a mixture of dichloromethane/ethanol/triethylamine, gradient 80:10:1–80:30:1. The titled compound was obtained as colorless powder, overall yield 80% (1.89 g, 8.00 mmol). Overall yield 1.89 g (8.00 mmol, 80%). Mp 73–74 °C; *R*_f_ 0.50 (CH_2_Cl_2_/EtOH/NEt_3_ 80:30:1, v/v/v); ^1^H NMR (400 MHz, CDCl_3_) δ 8.01 (d, *J* = 8.5 Hz, 1H, 9-H), 7.95 (d, *J* = 1.0 Hz, 1H, 4-H), 7.91 (d, *J* = 9.0 Hz, 1H, 6-H), 7.75 (dd, *J* = 8.8; 1.5 Hz, 1H, 8-H), 7.41 (d, *J* = 8.5 Hz, 1H, 3-H), 4.17 (s, 2H, CH_2_), 2.49 (s, 2H, NH); ^13^C NMR (101 MHz, CDCl_3_) δ 162.2, 146.4, 135.7, 133.1, 130.8, 129.8, 128.5, 120.8, 120.0, 48.1; ATR-FTIR (ZnSe) ν_max_: 3293, 2920, 2597, 1908, 1735, 1594, 1489, 1314, 1186, 1061 cm^−1^; HRESIMS (TOF) *m*/*z*: [M + H]^+^ calcd for C_10_H_10_BrN_2_, 237.0022; found, 237.0022.

### 3-Methylimidazo[1,5-*a*]pyridine (**16a**) [53]: typical procedure 2

A 5 mL Erlenmeyer flask equipped with a magnetic stirring bar was charged with nitroethane (**1a**, 150 mg, 2.00 mmol), 2-picolylamine (**12**, 108 mg, 1.00 mmol), 87% polyphosphoric acid (500 mg), and phosphorous acid (500 mg). The flask was capped with a septum, placed into an oil bath preheated to 160 °C, and stirred for 2 h. Then, the mixture was poured into ice-cold water (20 mL), neutralized with aqueous ammonia, and extracted with ethyl acetate (4 × 20 mL). The combined organic extracts were concentrated, the residue dried in vacuum, and then purified by preparative column chromatography on silica gel, eluting with a mixture of petroleum ether and ethyl acetate. The titled compound was obtained as yellow solid (lit. [53] yellow crystals), yield 77% (101 mg, 0.77 mmol). Mp 50–52 °C (lit. [53] mp 49–50 °C, cyclohexane); *R*_f_ 0.32 (EtOAc); ^1^H NMR (400 MHz, DMSO-*d*_6_) δ 8.04 (dd, *J* = 7.1; 1.0 Hz, 1H, 5-H), 7.48 (dt, *J* = 9.1; 1.1 Hz, 1H, 8-H), 7.24 (d, *J* = 0.6 Hz, 1H, 1-H), 6.69 (ddd, *J* = 9.1; 6.3; 0.8 Hz, 1H, 7-H), 6.61 (ddd, 1H, 6-H), 2.57 (s, 3H, CH_3_); ^13^C NMR (101 MHz, DMSO-*d*_6_) δ 134.8, 129.7, 121.7, 117.9, 117.72, 117.69, 111.8, 12.21. ATR-FTIR (ZnSe) ν_max_: 3041, 2924, 2857, 1635, 1494, 1442, 1400, 1357, 1322, 1267, 1248, 1170 cm^−1^; HRESIMS (TOF) *m*/*z*: [M + H]^+^ calcd for C_8_H_9_N_2_, 133.0760; found, 133.0758.

### 3-Ethylimidazo[1,5-*a*]pyridine (**16b**) [54]: typical procedure 3

A 5 mL Erlenmeyer flask equipped with a magnetic stirring bar was charged with 1-nitropropane (**1b**, 178 mg, 2.00 mmol), 4-methyl-*N*-(pyridin-2-ylmethyl)benzenesulfonamide (**17**, 262 mg, 1.00 mmol), and 87% polyphosphoric acid (2.00 g). The flask was capped with a septum, placed into an oil bath preheated to 160 °C, and stirred for 2 h. Upon reaction completion, the aqueous work-up, isolation, and purification of the product was performed in the same way as described in the typical procedure 1. The titled compound was obtained as yellow solid (lit. [54] yellow crystals), yield 66% (96 mg, 0.66 mmol). Alternatively, the same compound was obtained via the typical procedure 1 starting with 1-nitropropane (**1b**, 178 mg, 2.00 mmol) and 2-picolylamine (**12**, 108 mg, 1.00 mmol), yield 53% (77 mg, 0.53 mmol). Mp 59–62 °C (lit. [54] mp 61 °C, cyclohexane); *R*_f_ 0.27 (EtOAc/petroleum ether 1:1, v/v); ^1^H NMR (400 MHz, DMSO-*d*_6_) δ 8.10 (d, *J* = 6.6 Hz, 1H, 5-H), 7.49 (d, *J* = 8.9 Hz, 1H, 8-H), 7.26 (s, 1H, 1-H), 6.70 (t, 1H, 7-H), 6.61 (t, *J* = 6.0 Hz, 1H, 6-H), 2.96 (dd, *J* = 14.7; 7.3 Hz, 2H, CH_2_, 3-Et), 1.30 (t, *J* = 7.4 Hz, 3H, CH_3_, 3-Et); ^13^C NMR (101 MHz, DMSO-*d*_6_) δ 139.5, 129.8, 121.6, 118.1, 117.9, 117.6, 111.8, 19.1, 11.2; ATR-FTIR (ZnSe) ν_max_: 2975, 1638, 1504, 1491, 1454, 1367, 1326, 1241 cm^−1^; HRESIMS (TOF) *m*/*z*: [M + H]^+^ calcd for C_9_H_11_N_2_, 147.0917; found, 147.0913.

### 3-Phenylimidazo[1,5-*a*]pyridine (**16g**) [36]: typical procedure 4

A 5 mL Erlenmeyer flask equipped with a magnetic stirring bar was charged with 2-nitro-1-phenylethan-1-one (**1h**, 330 mg, 2.00 mmol), 2-picolylamine (**12**, 108 mg, 1.00 mmol), 87% polyphosphoric acid (500 mg), and phosphorous acid (500 mg). The flask was capped with a septum, placed into an oil bath preheated to 160 °C, and stirred for 2 h. Upon reaction completion, the aqueous work-up, isolation, and purification of the product were performed in the same way as described in the typical procedure 1. The titled compound was obtained as yellow solid (lit. [36] yellow solid), yield 76% (147 mg, 0.76 mmol). Alternatively, the same compound was obtained via the typical procedure 2 starting with (nitromethyl)benzene (274 mg, 2.00 mmol) and 2-picolylamine (**12**, 108 mg, 1.00 mmol), yield 12% (24 mg, 0.12 mmol). Mp 108–110 °C (lit. [36] mp 105–110 °C); *R*_f_ 0.51 (EtOAc/petroleum ether 1:1, v/v); ^1^H NMR (400 MHz, DMSO-*d*_6_) δ 8.44 (d, *J* = 7.2 Hz, 1H, 5-H), 7.83 (d, *J* = 7.3 Hz, 2H, 2,6-H 3-Ph), 7.64 (d, *J* = 9.1 Hz, 1H, 8-H), 7.55 (dd, *J* = 9.1; 6.1 Hz, 3H, 3,4,5-H 3-Ph), 7.46 (t, *J* = 7.4 Hz, 1H, 1-H), 6.83 (dd, *J* = 9.0; 6.4 Hz, 1H, 7-H), 6.71 (t, *J* = 6.8 Hz, 1H, 6-H); ^13^C NMR (101 MHz, DMSO-*d*_6_) δ 137.3, 131.4, 130.2, 129.0 (2C), 128.4, 127.6 (2C), 121.8, 120.4, 119.3, 118.5, 113.5; ATR-FTIR (ZnSe) ν_max_: 3066, 1749, 1676, 1603, 1473, 1458, 1433, 1358, 1305, 1251, 1267, 1120 cm^−1^; HRESIMS (TOF) *m*/*z*: [M + H]^+^ calcd for C_13_H_11_N_2_, 195.0917; found, 195.0918.

### 6-Bromo-7-methoxyimidazo[1,5-*a*]quinoline (**19de**)

The title compound was obtained via the typical procedure 2, starting from nitromethane (**1e**, 122 mg, 2.00 mmol) and (5-bromo-6-methoxyquinolin-2-yl)methanamine (**18d**, 267 mg, 1.00 mmol). Pale yellow solid, yield 72% (198 mg, 0.72 mmol). Mp 177–179 °C; *R*_f_ 0.43 (acetone/petroleum ether 1:1, v/v); ^1^H NMR (400 MHz, DMSO-*d*_6_) δ 9.11 (d, *J* = 0.8 Hz, 1H, 1-H), 8.42 (dd, *J* = 9.2; 0.7 Hz, 1H, 9-H), 7.48 (s, 1H, 3-H), 7.45 (d, *J* = 9.2 Hz, 1H, 4-H), 7.44 (s, 1H, 5-H), 7.37 (d, *J* = 9.8 Hz, 1H, 8-H), 3.96 (s, 3H, CH_3_); ^13^C NMR (101 MHz, DMSO-*d*_6_) δ 153.4, 129.7, 127.4, 125.7, 124.0, 123.0, 119.3, 118.8, 115.9, 113.4, 109.9, 56.9; ATR-FTIR (ZnSe) ν_max_: 3106, 2938, 2846, 1603, 1466, 1436, 1423, 1273, 1208, 1129, 1069 cm^−1^; HRESIMS (TOF) *m*/*z*: [M + H]^+^ calcd for C_12_H_10_BrN_2_O, 276.9971; found, 276.9974.

## Supporting Information

File 1 Synthetic procedures and characterization data for compounds **16c**–**f**, **18b** and **18d**, **19aa**, **19ab**, **19ac**, **19ag**, **19bb**, **19bg**, **19ce**, and **20** as well as ^1^H NMR, ^13^C NMR, and HRMS spectral charts for all new compounds.
