# Supplementary material for: Synthesis of imidazo[1,5-a]pyridines via cyclocondensation of 2-(aminomethyl)pyridines with electrophilically activated nitroalkanes
Source: Beilstein J Org Chem. 2020 Nov 26;16:2903–10. doi: 10.3762/bjoc.16.239 (PMC7705866; doi:10.3762/bjoc.16.239)
Supplement: File 1 — Synthetic procedures and characterization data for compounds 16c–f, 18b and 18d, 19aa, 19ab, 19ac, 19ag, 19bb, 19bg, 19ce, and 20 as well as 1H NMR, 13C NMR, and HRMS spectral charts for all new compounds. [file Beilstein_J_Org_Chem-16-2903-s001.pdf]

BEILSTEIN JOURNAL OF ORGANIC CHEMISTRY

Supporting Information

for

Synthesis of imidazo[1,5-*a*]pyridines via cyclocondensation of 2-(aminomethyl)pyridines with electrophilically activated nitroalkanes

Dmitrii A. Aksenov, Nikolai A. Arutiunov, Vladimir V. Maliuga, Alexander V. Aksenov and Michael Rubin

Beilstein J. Org. Chem. **2020,** *16,* 2903–2910. doi:10.3762/bjoc.16.239

Synthetic procedures and characterization data for compounds 16c–f, 18b and 18d, 19aa, 19ab, 19ac, 19ag, 19bb, 19bg, 19ce, and 20 as well as ^{1}H NMR, ^{13}C NMR, and HRMS spectral charts for all new compounds

License and Terms: This is a supporting information file under the terms of the Creative Commons Attribution License (https://creativecommons.org/licenses/by/4.0). Please note that the reuse, redistribution and reproduction in particular requires that the authors and source are credited.
The license is subject to the *Beilstein Journal of Organic Chemistry* terms and conditions: (https://www.beilstein-journals.org/bjoc)

Table of contents

Experimental procedures S2

Cited literature S5

NMR spectral charts S6

HRMS spectral charts S29

Experimental procedures

1-(Quinolin-2-yl)butan-1-amine (**18b**): Intermediate 2-(1-bromobutyl)quinoline (**21b**) was prepared according to typical procedure 1 starting with commercially available 2-butylquinoline (**22b**, 2.78 g, 15.0 mmol). Purification was performed by preparative column chromatography, eluting with EtOAc/hexane 1:10–1:6. Yellow oil, R_f 0.63, (EtOAc/petroleum ether, 1:6). Yield 2.68 g (10.2 mmol, 68%). ^{1}H NMR (400 MHz, DMSO) δ 8.39 (d, *J* = 8.4 Hz, 1H), 8.03-7.94 (m, 2H), 7.76 (ddd, *J* = 8.4, 6.9, 1.2 Hz, 1H), 7.70 (d, *J* = 8.5 Hz, 1H), 7.60 (ddd, *J* = 8.3, 6.9, 1.2 Hz, 1H), 5.38 (t, *J* = 7.5 Hz, 1H), 2.40–2.21 (m, 2H), 1.51-1.43 (m, 1H), 1.38–1.21 (m, 1H), 0.90 (t, *J* = 7.3 Hz, 3H); ^{13}C NMR (101 MHz, DMSO) δ 160.0, 146.6, 137.4, 130.0, 128.7, 127.8, 127.2, 127.0, 120.3, 56.3, 38.8, 20.8, 13.2; ATR-FTIR (ZnSe) *ν* (cm^{-1}): 3062, 2970, 2934, 2876, 1626, 1604, 1566, 1505, 1469, 1433, 1380, 1310, 1187, 829, 757. HRMS (ESI-TOF): m/z (M+H)$^+$, Calcd. for $C_{13}H_{15}BrN$: 264.0382; Found: 264.0376. The title compound was obtained as yellow oil, R_f 0.54, (CH_2Cl_2/EtOH/NEt_3 80:20:1). Purification was performed by preparative column chromatography, eluting with CH_2Cl_2/EtOH/NEt_3 80:10:1–80:20:1. Yield 1.90 g (9.50 mmol, 95%). ^{1}H NMR (400 MHz, $CDCl_3$) δ 8.08 (d, *J* = 8.5 Hz, 1H, 8-H), 8.04 (d, *J* = 8.5 Hz, 1H, 4-H), 7.76 (d, *J* = 8.1 Hz, 1H, 5-H), 7.69–7.64 (m, 1H, 7-H), 7.48 (t, *J* = 7.5 Hz, 1H, 6-H), 7.39 (d, *J* = 8.5 Hz, 1H, 3-H), 4.18 (t, *J* = 6.7 Hz, 1H, CH- 2-Bu), 2.61 (s, 2H, NH), 1.87–1.68 (m, 2H, CH_2- 2-Bu), 1.47–1.26 (m, 2H, CH_2- 2-Bu), 0.91 (t, *J* = 7.4 Hz, 3H, CH_3- 2-Bu). ^{13}C NMR (101 MHz, $CDCl_3$) δ 164.9, 147.7, 136.6, 129.5, 129.2, 127.6, 127.4, 126.1, 119.4, 57.5, 40.7, 19.6, 14.1. ATR-FTIR (ZnSe) *ν* (cm^{-1}): 3370, 3062, 2964, 2976, 1619, 1601, 1563, 1502, 1429,1308, 1123. HRMS (ESI-TOF): m/z (M+Na)$^+$, Calcd. for $C_{13}H_{16}N_2Na$: 223.1206; Found: 223.1204.

(5-Bromo-6-methoxyquinolin-2-yl)methanamine (**18d**): Intermediate 5-bromo-2-(bromomethyl)-6-methoxyquinoline (**21d**) was prepared according to typical procedure 1 starting with commercially available 5-bromo-6-methoxy-2-methylquinoline (**22d**, 3.78 g, 15 mmol). Purification was performed by preparative column chromatography, eluting with EtAOAc/hexane, gradient 1:6–1:3. Yellow solid, mp 130-132 °C, R_f 0.37 (EtOAc/hexane, 1:3). Yield 4.47 g (13.5 mmol, 90%). ^{1}H NMR (400 MHz, $CDCl_3$) δ 8.53 (d, *J* = 8.8 Hz, 1H, 4-H), 8.07 (d, *J* = 9.3 Hz, 1H, 8-H), 7.61 (d, *J* = 8.8 Hz, 1H, 7-H), 7.51 (d, *J* = 9.3 Hz, 1H, 3-H), 4.71 (s, 2H, -CH_2-), 4.05 (s, 3H, OCH_3); ^{13}C NMR (101 MHz, $CDCl_3$) δ 155.3, 154.6, 143.3, 136.2, 130.0, 128.0, 122.7, 117.0, 107.5, 57.2, 33.8. ATR-FTIR (ZnSe) *ν* (cm^{-1}): 2935, 1613, 1587, 1491, 1446, 1259, 1219, 1147, 1042. HRMS (ESI-TOF): m/z (M+Na)$^+$, Calcd. for $C_{11}H_{10}Br_2NO$: 329.9124; Found: 329.9118. The title compound was obtained as pale brown solid, mp 75-77 °C, R_f 0.30 (CH_2Cl_2/EtOH 5:1). Yield 3.06 g (11.5 mmol, 85%). ^{1}H NMR (400 MHz, DMSO-*d*$_6$) δ 8.40 (d, *J* = 8.8 Hz, 1H, 4-H), 8.02 (d, *J* = 9.3 Hz, 1H, 8-H), 7.73 (d, *J* = 9.3 Hz, 1H, 7-H), 7.70 (d, *J* = 8.8 Hz, 1H, 3-H), 4.04 (s, 2H, -CH_2-), 4.01 (s, 3H, -OCH_3); ^{13}C NMR (101 MHz, DMSO-*d*$_6$) δ 160.5, 153.4, 142.8, 134.0, 129.6, 126.7, 121.8, 117.2, 106.1, 57.0, 46.8; ATR-FTIR (ZnSe) *ν* (cm^{-1}): 3102, 2974, 2842, 1593, 1557, 1493, 1469, 1436, 1311, 1269, 1209, 1126, 1062. HRMS (ESI-TOF): m/z (M+H)$^+$, Calcd. for $C_{11}H_{12}BrN_2O$ 267.0128; Found: 267.0127.

3-Propylimidazo[1,5-*a*]pyridine (**16c**):[S1] The title compound was obtained according to the typical procedure 2 starting with 1-nitrobutane (**1c**, 206 mg, 2.00 mmol) and 2-picolylamine (**12**, 108 mg, 1.00 mmol). Yellow oil, R_f 0.27 (EtOAc/ petroleum ether, 1:1). Yield 94 mg (0.59 mmol, 59%). Alternatively, the same compound was prepared via typical procedure 3 starting with 4-methyl-*N*-(pyridin-2-ylmethyl)-benzenesulfonamide (**17**, 262 mg, 1.00 mmol) and 1-nitrobutane (**1c**, 206 mg, 2.00 mmol). Yield 107 mg (0.67 mmol, 67%). ^{1}H NMR (400 MHz, DMSO-*d*$_6$) δ 8.12 (d, *J* = 7.2 Hz, 1H, 5-H), 7.48 (d, *J* = 9.0 Hz, 1H, 8-H), 7.25 (s, 1H, 1-H), 6.68 (dd, *J* = 9.0, 6.4 Hz, 1H, 7-H), 6.62–6.55 (m, 1H, 6-H), 2.92 (t, *J* = 7.4 Hz, 2H, -CH_2- 3-Pr), 1,90-1,44 (m, 2H, -CH_2- 3-Pr), 0.94 (t, *J* = 7.4 Hz, 3H, -CH_3- 3-Pr). ^{13}C NMR (101 MHz, DMSO-*d*$_6$) δ 138.5, 129.7, 121.6, 118.0, 117.84, 117.77, 111.8, 27.6, 20.11, 13.7. ATR-FTIR (ZnSe) *ν* (cm^{-1}): 3114, 2967, 1685, 1652, 1509, 1488, 1455, 1339, 1325, 1273. HRMS (ESI-TOF): m/z (M+H)$^+$, Calcd. for $C_{10}H_{13}N_2$: 161.1073; Found: 161.1070.

3-Pentylimidazo[1,5-*a*]pyridine (**16d**): The title compound was obtained according to the typical procedure 2 starting with 1-nitrohexane (**1d**, 262 mg, 2.00 mmol) and 2-picolylamine (**12**, 108 mg, 1.00 mmol). Yellow oil, R_f 0.61 (EtOAc/petroleum ether 1:1). Yield 105 mg (0.56 mmol, 56 %). Alternatively, the same compound was prepared via typical procedure 3 starting with 4-methyl-*N*-(pyridin-2-ylmethyl)benzenesulfonamide (**17**, 262 mg, 1.00 mmol), and 1-nitrohexane (**1d**, 262 mg, 2.00 mmol). Yield 120 mg (0.64 mmol, 64%). ^{1}H NMR (400 MHz, DMSO-d_6) δ 8.11 (d, *J* = 7.3 Hz, 1H, 5-H), 7.48 (d, *J* = 9.2 Hz, 1H, 8-H), 7.25 (s, 1H, 1-H), 6.68 (dd, *J* = 9.2, 6.1 Hz, 1H, 7-H), 6.59 (t, *J* = 6.7 Hz, 1H, 6-H), 2.93 (t, *J* = 7.5 Hz, 2H, -CH_2- 3-Am), 1.78–1.67 (m, 2H, -CH_2- 3-Am), 1.36–1.28 (m, 4H, -CH_2- 3-Am), 0.85 (t, *J* = 6.6 Hz, 3H, -CH_3- 3-Am).^{13}C NMR (101 MHz, DMSO-d_6) δ 138.6, 129.7, 121.6, 118.1, 117.84, 117.76, 111.8, 30.9, 26.4, 25.6, 21.9, 13.9.ATR-FTIR (ZnSe) *ν* (cm^{-1}): 2953, 2927, 2868, 1698, 1650, 1557, 1504, 1458, 1363, 1326, 1272. HRMS (ESI-TOF): m/z (M+H)$^+$, Calcd. for $C_{12}H_{17}N_2$ + ([M+H]+): 189.1386, found: 189.1381 (*δ* =2.6 ppm).

Imidazo[1,5-*a*]pyridine (**16e**):[S2] The title compound was obtained via typical procedure 2 starting from nitromethane (**1e**, 122 mg, 2.00 mmol) and 2-picolylamine (**12**, 108 mg, 1.00 mmol). Light-brown solid, R_f 0.27 (EtOAc), mp 51-52, lit[S2] m.p. 52-54 °C ($CHCl_3$, hexane). Yield 73 mg (0.62 mmol, 62 %). ^{1}H NMR (400 MHz, DMSO-d_6) δ 8.36 (s, 1H, 3-H), 8.32 (d, *J* = 7.0 Hz, 1H, 5-H), 7.52 (d, *J* = 9.1 Hz, 1H, 8-H), 7.34 (s, 1H, 1-H), 6.74 (dd, *J* = 8.9, 6.4 Hz, 1H, 7-H), 6.62 (t, *J* = 6.7 Hz, 1H, 6-H). ^{13}C NMR (101 MHz, DMSO-d_6) δ 129.6, 128.2, 123.2, 119.2, 119.0, 117.8, 112.2.ATR-FTIR (ZnSe) *ν* (cm^{-1}): 3052, 2935, 2857, 1651, 1504, 1455, 1370, 1327, 1246, 1221, 1113. HRMS (ESI-TOF): m/z (M+H)$^+$, Calcd. for $C_7H_7N_2$: 119.0604; Found: 119.0602.

3-Benzylimidazo[1,5-*a*]pyridine (**16f**):[S1] The title compound was obtained via typical procedure 2 starting from (2-nitroethyl)benzene (**1f**, 302 mg, 2.00 mmol) and 2-picolylamine (**12**, 108 mg, 1.00 mmol). Yellow solid, R_f 0.58 (EtOAc/petroleum ether, 1:1), mp 84-87 °C, lit[S1] yellow crystals mp 84-87 °C(cyclohexane). Yield 112 mg (0.54 mmol, 54%). ^{1}H NMR (400 MHz, DMSO-d_6) δ 8.09 (dd, *J* = 7.2, 1.1 Hz, 1H, 5-H), 7.52 (dt, *J* = 9.1, 1.2 Hz, 1H, 8-H), 7.32 (d, *J* = 1.0 Hz, 1H, 1-H), 7.30–7.25 (m, 2H, 2,6-H 3-Bn), 7.24–7.18 (m, 3H, 3,4,5-H 3-Bn), 6.71 (ddd, *J* = 9.1, 6.4, 1.0 Hz, 1H, 7-H), 6.59 (ddd, *J* = 7.4, 6.3, 1.2 Hz, 1H, 6-H), 4.41 (s, 2H, -CH_2- 2-Bn); ^{13}C NMR (101 MHz, DMSO-d_6) δ 137.26, 137.17, 130.1, 128.5 (2C), 128.3 (2C), 126.4, 121.5, 118.3, 118.1 (2C), 112.1, 32.0; ATR-FTIR (ZnSe) *ν* (cm^{-1}): 3026, 2971, 2934, 1683, 1557, 1507, 1456, 1361, 1336, 1277. HRMS (ESI-TOF): m/z (M+H)$^+$, Calcd. for $C_{14}H_{13}N_2$: 209.1073; Found: 209.1075.

1-Methylimidazo[1,5-*a*]quinoline (**19aa**):[S3] The title compound was obtained via typical procedure 2 starting from nitroethane (**1a**, 150 mg, 2.00 mmol) and quinolin-2-ylmethanamine (**18a**, 158 mg, 1.00 mmol). Yellow oil, R_f 0.36 (EtOAc). Yield 111 mg (0.61 mmol, 61%). ^{1}H NMR (400 MHz, $CDCl_3$) δ 8.07 (d, *J* = 8.4 Hz, 1H, 9-H), 7.51 (d, *J* = 7.7 Hz, 1H, 6-H), 7.39 (ddd, *J* = 8.7, 7.3, 1.7 Hz, 1H, 7-H), 7.28 (t, *J* = 7.5 Hz, 1H, 8-H), 7.24 (s, 1H, 3-H), 7.13 (d, *J* = 9.4 Hz, 1H, 5-H), 6.80 (d, *J* = 9.4 Hz, 1H, 4-H), 2.98 (s, 3H, CH_3).^{13}C NMR (101 MHz, $CDCl_3$) δ 140.0, 133.2, 130.3, 128.5, 127.6, 125.6, 124.8, 120.55, 120.52, 117.2, 116.1, 19.6. ATR-FTIR (ZnSe) *ν* (cm^{-1}): 3059, 1606, 1559, 1478, 1453, 1387, 1306, 1268, 1211. HRMS (ESI-TOF): m/z (M+H)$^+$, Calcd. for $C_{12}H_{11}N_2$: 183.0917; Found: 183.0918.

1-Ethylimidazo[1,5-a]quinoline (**19ab**):[S4] The title compound was obtained via typical procedure 2 starting from 1-nitropropane (**1b**, 178 mg, 2.00 mmol) and quinolin-2-ylmethanamine (**18a**, 158 mg, 1.00 mmol). Yellow oil, R_f 0.31, (EtOAc/petroleum ether, 1:4). Yield 90 mg (0.46 mmol, 46%). ^{1}H NMR (400 MHz, $CDCl_3$) δ 8.15 (d, *J* = 8.5 Hz, 1H, 9-H), 7.63 (dd, *J* = 7.7, 1.7 Hz, 1H, 7-H), 7.52 (ddd, *J* = 8.7, 7.3, 1.7 Hz, 1H, 8-H), 7.40 (dd, *J* = 7.5, 1.0 Hz, 1H, 6-H), 7.38 (s, 1H, 3-H), 7.23 (d, *J* = 2.4 Hz, 1H, 4-H), 6.96 (d, *J* = 9.3 Hz, 1H, 5-H), 3.45 (q, *J* = 7.4 Hz, 2H, CH_2 1-Et), 1.57 (t, *J* = 7.4 Hz, 3H, CH_3 1-Et); ^{13}C NMR (101 MHz, $CDCl_3$) δ 145.0, 133.1, 130.4, 129.0, 128.2, 126.0, 125.4, 121.5, 119.5, 117.4, 116.8, 25.6, 12.0. ATR-FTIR (ZnSe) *ν* (cm^{-1}): 2978, 2927, 1559, 1473, 1391, 1376, 1326, 1304, 1268, 1217, 1162, 1056. HRMS (ESI-TOF): Calcd. for m/z (M+H)$^+$, $C_{13}H_{13}N_2{}^+$ 197.1073; Found: 197.1069.

1-Propylimidazo[1,5-*a*]quinoline (**19ac**):[S3] The title compound was obtained via typical procedure 2 starting from 1-nitrobutane (**1c**, 206 mg, 2.00 mmol) and quinolin-2-ylmethanamine (**18a**, 158 mg, 1.00 mmol). Colorless oil, R_f 0.42, (EtOAc/petroleum ether, 1:1). Yield 117 mg (0.56 mmol, 56%). ^{1}H NMR (400 MHz, DMSO-*d*$_6$) δ 8.23 (d, *J* = 8.5 Hz, 1H, 9-H), 7.79 (dd, *J* = 7.7, 1.4 Hz, 1H, 6-H), 7.62 (ddd, *J* = 8.6, 7.3, 1.5 Hz, 1H, 7-H), 7.48–7.44 (m, 1H, 8-H), 7.42 (d, *J* = 9.4 Hz, 1H, 5-H), 7.34 (s, 1H, 3-H), 7.09 (d, *J* = 9.4 Hz, 1H, 4-H), 3.33 (t, 2H, -CH_2- 1-Pr), 1.98-1.80 (m, 2H, -CH_2- 1-Pr), 1.07 (t, *J* = 7.4 Hz, 3H, -CH_3- 1-Pr). ^{13}C NMR (101 MHz, DMSO-*d*$_6$) δ 143.6, 132.4, 129.8, 128.6, 128.2, 125.3, 125.0, 120.6, 120.3, 117.4, 116.7, 33.5, 20.1, 13.8.ATR-FTIR (ZnSe) *ν* (cm^{-1}): 2967, 2890, 2810, 1665, 1477, 1453, 1391, 1264, 1211,1160. HRMS (ESI-TOF): m/z (M+H)$^+$, $C_{14}H_{15}N_2$: 211.1230; Found: 211.1228.

Imidazo[1,5-*a*]quinoline (**19ae**):[S5] The title compound was obtained via typical procedure 2 starting from nitromethane (**1e**, 122 mg, 2.00 mmol) and quinolin-2-ylmethanamine (**18a**, 158 mg, 1.00 mmol). Yellow solid, R_f 0.33, (EtOAc/petroleum ether, 1:1), m.p. 71-72 °C, lit[S4] yellow crystals mp 73-75 °C. Yield 139 mg (0.83 mmol, 83%). ^{1}H NMR (400 MHz, DMSO-*d*$_6$) δ 9.53 (s, 1H, 1-H), 8.43 (d, *J* = 8.1 Hz, 1H, 9-H), 7.88 (d, *J* = 7.0 Hz, 1H, 5-H), 7.69 (dd, *J* = 8.4, 5.2 Hz, 2H, 7,3-H), 7.56 (dd, *J* = 8.1, 5.6 Hz, 2H, 8,6-H), 7.33 (d, *J* = 9.5 Hz, 1H, 4-H). ^{13}C NMR (101 MHz, DMSO-*d*$_6$) δ 130.3, 129.2, 128.9, 128.8, 128.1, 126.5, 123.8, 122.4, 119.4, 116.6, 115.9. ATR-FTIR (ZnSe) *ν* (cm^{-1}): 3125, 2927, 1740, 1555, 1478, 1451, 1240, 1217, 1142,1114. HRMS (ESI-TOF): m/z (M+H)$^+$, $C_{11}H_9N_2$: 169.0760; Found: 169.0761.

1-Phenylimidazo[1,5-a]quinoline (**19ag**):[S4] The title compound was obtained via typical procedure 4 starting from 2-nitro-1-phenylethan-1-one (**1h**, 330 mg, 2.00 mmol) and quinolin-2-ylmethanamine (**18a**, 158 mg, 1.00 mmol). Yellow solid, R_f 0.51 (EtOAc), mp 116-117 °C, lit[S5] yellow solid, mp 113-115°C. Yield 200 mg (0.84 mmol, 84%). ^{1}H NMR (400 MHz, DMSO-*d*$_6$) δ 7.81 (dd, *J* = 7.7, 1.6 Hz, 1H, 9-H), 7.63–7.57 (m, 5H, 1-Ph), 7.56 (s, 1H, 3-H), 7.53 (d, *J* = 9.4 Hz, 1H, 5-H), 7.43–7.33 (m, 2H, 6,8-H), 7.27 (ddd, *J* = 8.7, 7.0, 1.6 Hz, 1H, 7-H), 7.22 (d, *J* = 9.4 Hz, 1H, 4-H). ^{13}C NMR (101 MHz, DMSO-*d*$_6$) δ 141.7, 133.8, 131.7, 130.1, 129.51 (2C), 129.45, 128.99, 128.91 (2C), 127.6, 125.4, 125.2, 122.3, 121.4, 117.3, 116.53.ATR-FTIR (ZnSe) *ν* (cm^{-1}): 3102, 2974, 2842, 1593, 1557, 1493, 1469, 1436, 1311, 1269, 1209, 1126, 1062 HRMS (ESI-TOF): m/z (M+H)$^+$, $C_{17}H_{13}N_2$: 245.1073; Found 245.1074.

1-Ethyl-3-propylimidazo[1,5-*a*]quinoline (**19bb**): The title compound was obtained via typical procedure 2 starting from 1-nitropropane (**1b**, 178 mg, 2.00 mmol) and 1-(quinolin-2-yl)butan-1-amine (**18b**, 200 mg, 1.00 mmol). Yellow oil, R_f 0.60, (EtOAc/petroleum ether, 1:4). Yield 150 mg (0.63 mmol, 63 %). ^{1}H NMR (400 MHz, $CDCl_3$) δ 8.04 (d, *J* = 8.5 Hz, 1H, 9-H), 7.54 (dd, *J* = 7.7, 1.3 Hz, 1H, 5-H), 7.43 (ddd, *J* = 8.6, 4.5, 0.7 Hz, 1H, 8-H), 7.30 (td, *J* = 7.4, 0.6 Hz, 1H, 7-H), 7.16 (d, *J* = 9.4 Hz, 1H, 6-H), 6.77 (d, *J* = 9.4 Hz, 1H, 4-H), 3.36 (q, *J* = 7.4 Hz, 2H, CH_2- 1-Et), 2.82–2.74 (m, 2H, CH_2- 3-Pr), 1.82–1.69 (m, 2H, CH_2- 3-Pr), 1.51 (t, *J* = 7.4 Hz, 3H, CH_3- 1-Et), 0.97 (t, *J* = 7.4 Hz, 3H, CH_3- 3-Pr). ^{13}C NMR (101 MHz, $CDCl_3$) δ 143.9, 133.32, 133.31, 128.5, 127.5, 126.3, 126.1, 124.6, 118.9, 117.1, 116.5, 29.2, 25.6, 23.7, 14.1, 12.2. ATR-FTIR (ZnSe) *ν* (cm^{-1}): 2960, 2876, 1887, 1696, 1625, 1557, 1480, 1455, 1389, 1374,1218, 1136,1114. HRMS (ESI-TOF): m/z (M+H)$^+$, $C_{16}H_{19}N_2$: 239.1543; Found: 239.1539.

3-Propylimidazo[1,5-*a*]quinoline (**19be**): The title compound was obtained via typical procedure 2 starting from nitromethane (**1e**, 122 mg, 2.00 mmol) and 1-(quinolin-2-yl)butan-1-amine (**18b**, 200 mg, 1.00 mmol). Yellow oil, R_f 0.38, (EtOAc/petroleum ether, 1:1). Yield 100 mg (0.48 mmol, 48%). ^{1}H NMR (400 MHz, DMSO-*d*$_6$) δ 8.98 (s, 1H, 1-H), 8.29 (d, *J* = 8.3 Hz, 1H, 9-H), 7.79–7.73 (m, 1H, 6-H), 7.58 (ddd, *J* = 8.4, 7.2, 1.3 Hz, 1H, 7-H), 7.46–7.44 (m, 1H, 8-H), 7.42 (d, *J* = 8.1 Hz, 1H, 5-H), 7.05 (d, *J* = 9.5 Hz, 1H, 4-H), 2.76 (t, *J* = 7.3 Hz, 2H, -CH_2- 3-Pr), 1.96-1.44 (m, 2H, -CH_2- 3-Pr), 0.91 (t, *J* = 7.4 Hz, 3H, -CH_3- 3-Pr). ^{13}C NMR (101 MHz, DMSO-*d*$_6$) δ 134.7, 130.7, 128.5 (2C), 128.0, 125.3, 124.2, 123.7, 119.1, 116.6, 115.1, 28.4, 22.8, 13.7. ATR-FTIR (ZnSe) *ν* (cm^{-1}): 3114, 2964, 2868, 1683, 1557, 1489, 1458, 1378, 1339, 1225, 1129. HRMS (ESI-TOF): m/z (M+H)$^+$, Calcd. for $C_{14}H_{15}N_2$: 211.1230; Found: 211.1234.

1-Phenyl-3-propylimidazo[1,5-a]quinoline (**19bg**): The title compound was obtained via typical procedure 4 starting from 2-nitro-1-phenylethan-1-one (**1h**, 330 mg, 2.00 mmol) and 1-(quinolin-2-yl)butan-1-amine

(**18b**, 200 mg, 1.00 mmol). Yellow oil, R_f 0.34, (EtOAc/petroleum ether, 1:8). Yield 75 mg (0.26 mmol, 53%). ^{1}H NMR (400 MHz, $CDCl_3$) δ 7.67–7.62 (m, 2H, 2,6-H Ph), 7.57 (dd, *J* = 7.8, 1.6 Hz, 1H, 6-H), 7.52–7.48 (m, 3H, 3,4,5-H Ph), 7.46 (s, 1H, 9-H), 7.31–7.28 (m, 1H, 5-H), 7.28–7.24 (m, 1H, 7-H), 7.13 (ddd, *J* = 8.7, 7.2, 1.6 Hz, 1H, 8-H), 6.92 (d, *J* = 9.4 Hz, 1H, 4-H), 2.88 (t, 2H, CH_2- 3-Pr), 1.90-1.76 (m, 2H, CH_2- 3-Pr), 1.01 (t, *J* = 7.3 Hz, 3H, CH_3- 3-Pr). ^{13}C NMR (101 MHz, $CDCl_3$) δ 141.0, 135.1, 133.8, 132.6, 129.8 (2C), 129.3, 128.9 (2C), 128.6, 127.2, 126.7, 125.9, 125.1, 119.9, 117.5, 116.9, 29.3, 23.6, 14.2. ATR-FTIR (ZnSe) *ν* (cm^{-1}): 3062, 2956, 2872, 1601, 1553, 1486, 1455, 1374, 1317, 1253, 1213, 1145, 1076. HRMS (ESI-TOF): m/z $(M+H)^+$, Calcd. for $C_{20}H_{19}N_2$: 287.1543; Found: 287.1539.

6-Bromoimidazo[1,5-a]quinoline (**19ce**): The title compound was obtained via typical procedure 2 starting from nitromethane (**1e**, 122 mg, 2.00 mmol) and (6-bromoquinolin-2-yl)methanamine (**18c**, 237 mg, 1.00 mmol). Colorless solid, mp 152-153 °C, R_f 0.50, (EtOAc). Yield 144 mg (0.59 mmol, 59%). ^{1}H NMR (400 MHz, $CDCl_3$) δ 8.62 (s, 1H, 1-H), 7.83 (d, *J* = 8.8 Hz, 1H, 9-H), 7.80 (d, *J* = 2.2 Hz, 1H, 6-H), 7.64 (dd, *J* = 8.8, 2.2 Hz, 1H, 8-H), 7.48 (s, 1H, 3-H), 7.35 (d, *J* = 9.5 Hz, 1H, 4-H), 6.93 (d, *J* = 9.4 Hz, 1H, 5-H). ^{13}C NMR (101 MHz, $CDCl_3$) δ 131.5, 131.2, 130.0, 128.6, 128.2, 126.1, 123.4, 120.1, 118.7, 118.3, 116.4. ATR-FTIR (ZnSe) *ν* (cm^{-1}): 3132, 1901, 1707, 1546, 1478, 1422, 1361, 1325, 1202, 1126,1107. HRMS (ESI-TOF): m/z $(M+H)^+$, Calcd. for $C_{11}H_8BrN_2$: 246.9865; Found: 246.9864.

Ethyl 2-oxo-2-((pyridin-2-ylmethyl)amino)acetate (**20**):[S6] The title compound was obtained via typical procedure 2 starting from ethyl 2-nitroacetate (**1i**, 266 mg, 2.00 mmol) and 2-picolylamine (**12**, 108 mg, 1.00 mmol). The reaction was carried out at 140 °C. Yellow powder, R_f 0.22, (EtOAc/ petroleum ether, 1:1), mp 57-59 °C, lit[S6] yellow crystals, mp 62-63.5 °C (EtOH). Yield 60 mg (0.29 mmol, 29%). ^{1}H NMR (400 MHz, DMSO-d_6) δ 9.42 (s, 1H, NH), 8.50 (d, *J* = 4.6 Hz, 1H, 6-H py), 7.76 (td, *J* = 7.7, 1.6 Hz, 1H, 4-H py), 7.30 (d, *J* = 4.1 Hz, 3-H py), 7.27 (t, *J* = 8.1 Hz, 2H, 5-H py), 4.44 (d, *J* = 6.1 Hz, 2H, CH_2), 4.26 (q, *J* = 7.1 Hz, 2H, CH_2), 1.28 (t, *J* = 7.1 Hz, 3H, CH_3). ^{13}C NMR (101 MHz, DMSO-d_6) δ 160.6, 157.3, 157.2, 148.9, 136.8, 122.3, 121.1, 62.1, 44.3, 13.8. ATR-FTIR (ZnSe) *ν* (cm^{-1}): 3407, 2931, 1735, 1700, 1650, 1541, 1520, 1471, 1453, 1369,1206. HRMS (ESI-TOF): m/z $(M+Na)^+$, $C_{10}H_{12}N_2NaO_3$: 231.0740; Found: 231.0737.

Cited literature

S1. Li, M.; Xie, Y.; Ye, Y.; Zou, Y.; Jiang, H.; Zeng, W. *Org. Lett.* **2014,** *16*, 6232-6235.

S2. Catalan, J.; De Paz, J. L. G.; Yanez, M.; Amat-Guerri, F.; Houriet, R.; Rolli, E.; Zehringer, R.; Oelhafen, P.; Taft, R. W.; Anvia, F.; Qian, J. H. *J. Am. Chem. Soc.* **1988,** *110*, 2699-2705.

S3. Wang, Q., Zhang, S., Guo, F., Zhang, B., Hu, P., & Wang, Z. *J. Org. Chem*. **2012**, *72*, 11161–11166.

S4. Li, Z.; Wu, S.-S.; Luo, Z.-G.; Liu, W.-K.; Feng, C.-T.; Ma, S.-T. *J. Org. Chem.* **2016,** *81*, 4386-4392.

S5. Van Nispen, S. P. J. M.; Mensink, C.; Van Leusen, A. M. *Tetrahedron Lett.* **1980,** *21*, 3723-3726.

S6. Winterfeld, K.; Lampke, H.; Franzke, H. *Justus Liebigs Ann. Chem.* **1965,** *685*, 181-186.

NMR spectral charts

^{1}H (400 MHz) and ^{13}C (101 MHz) NMR spectra of **21b** in $CDCl_3$.

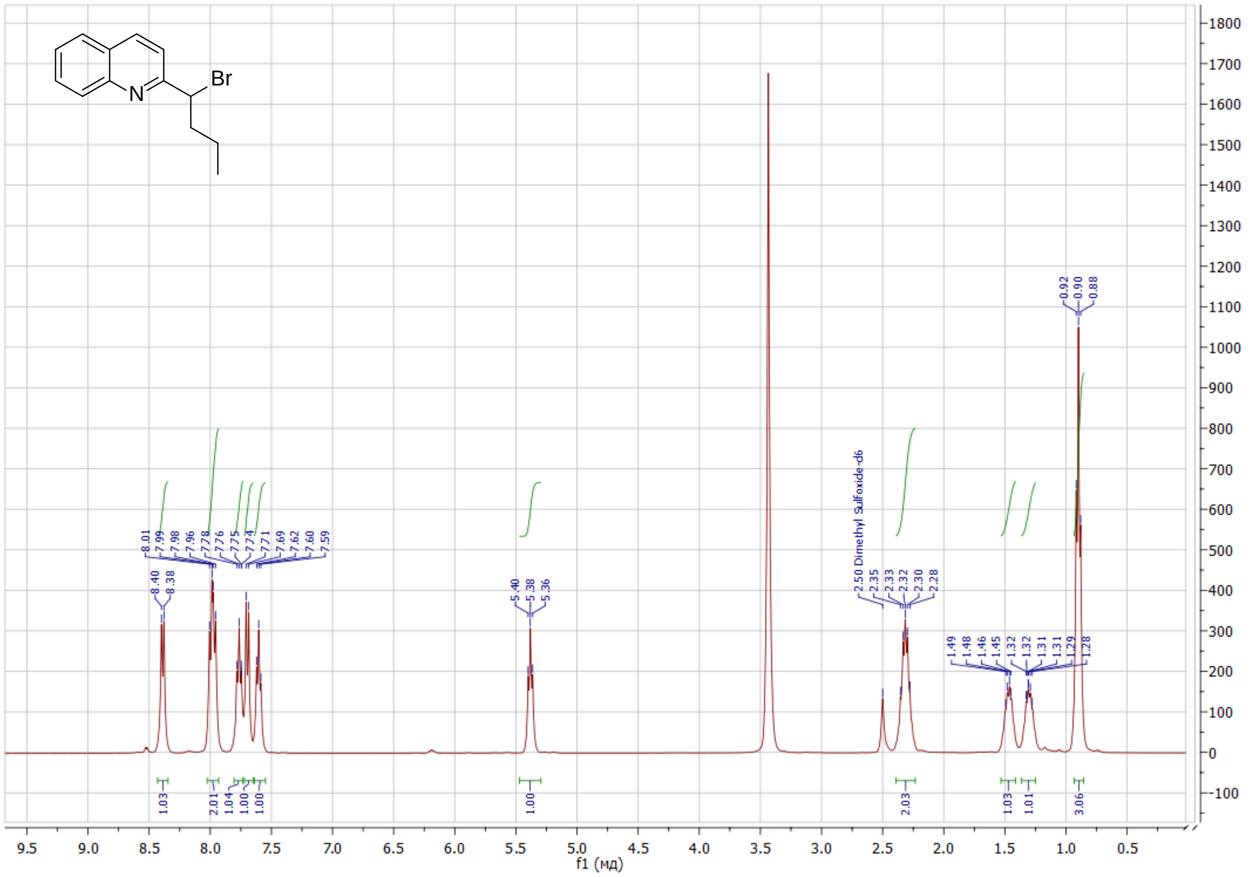

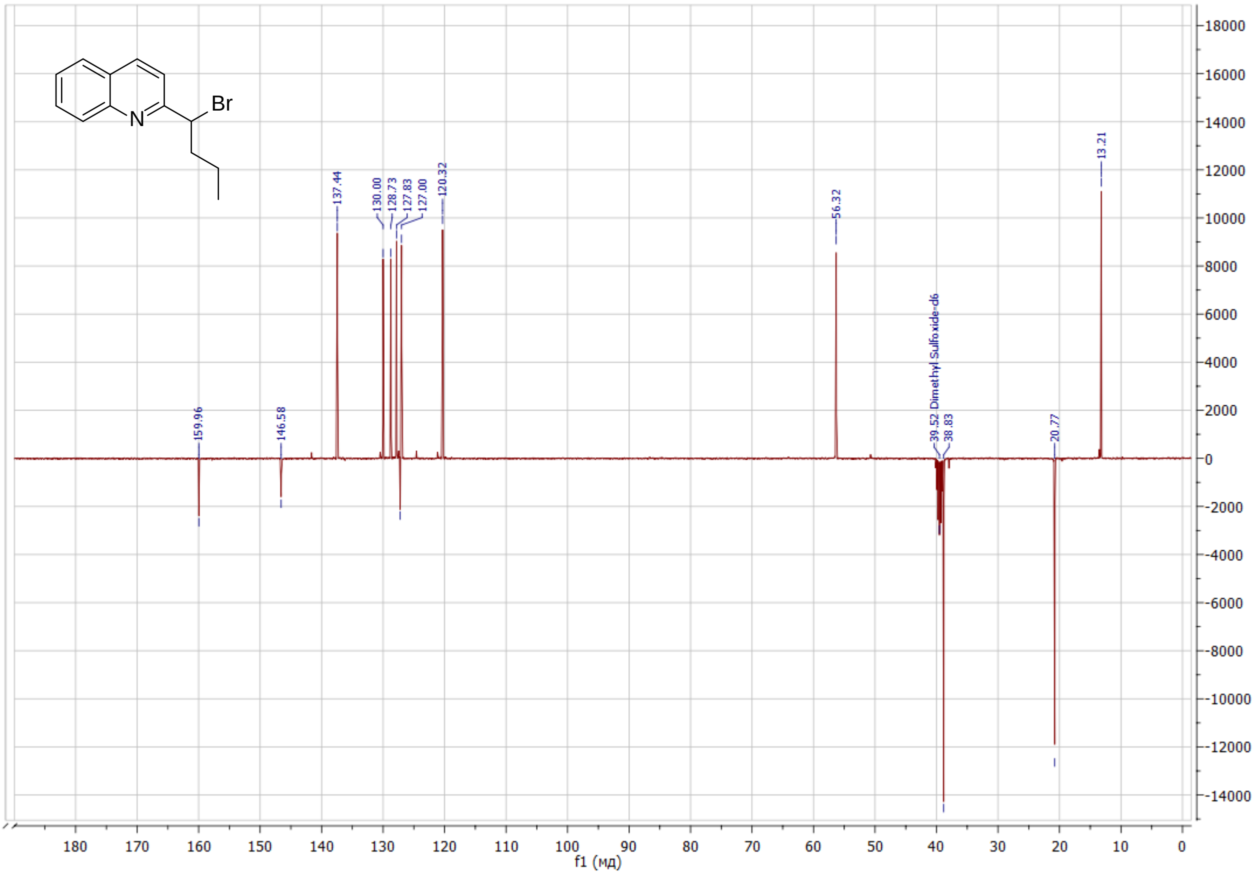

^{1}H (400 MHz) and ^{13}C (101 MHz) NMR spectra of **21d** in $CDCl_3$.

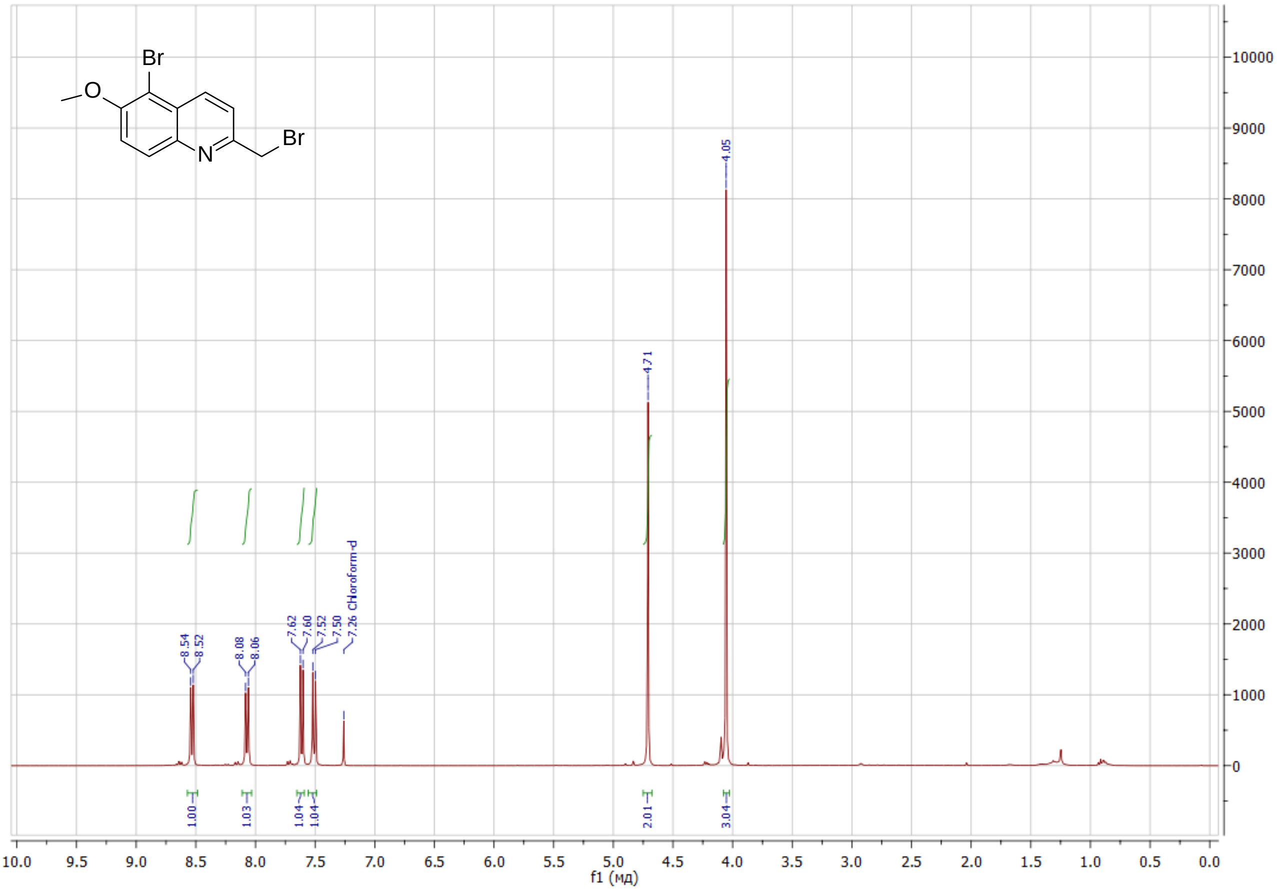

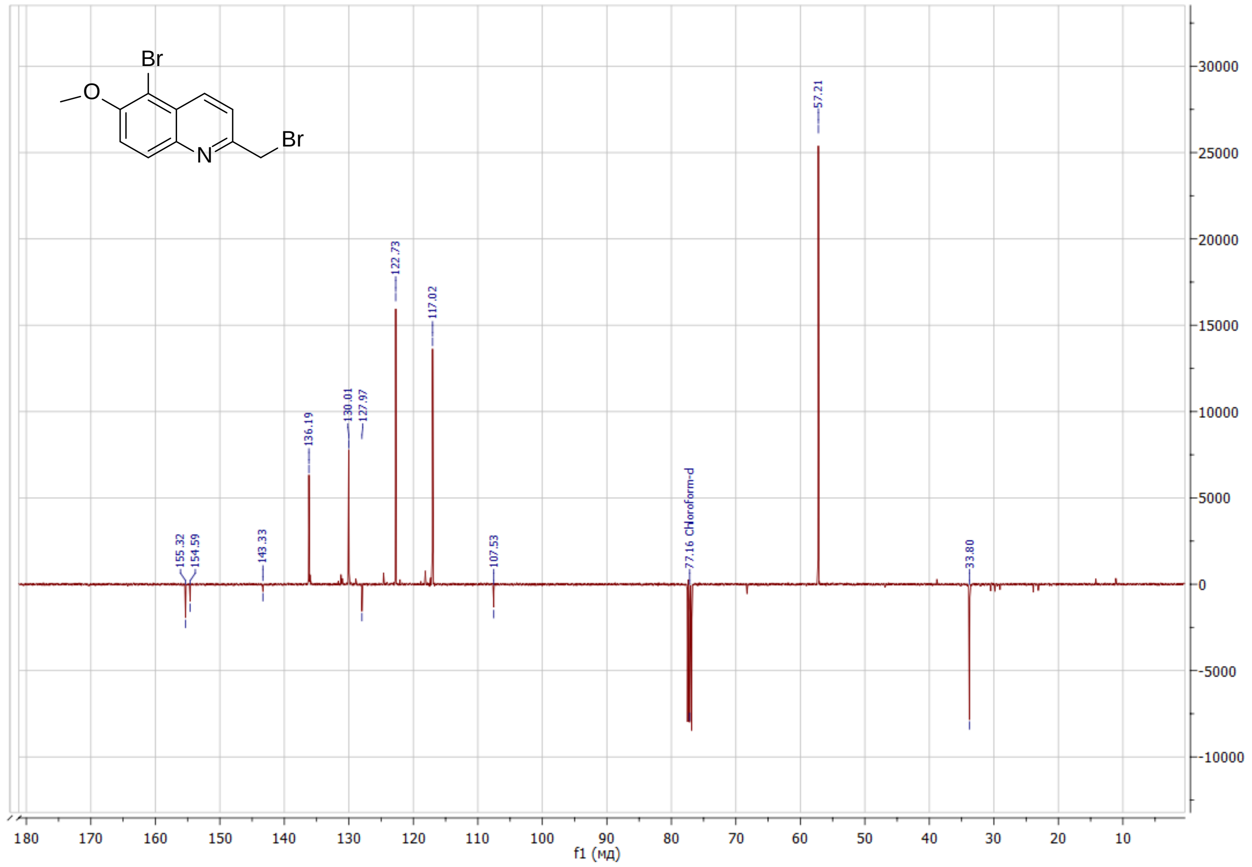

^{1}H (400 MHz) and ^{13}C (101 MHz) NMR spectra of **18b** in $CDCl_3$.

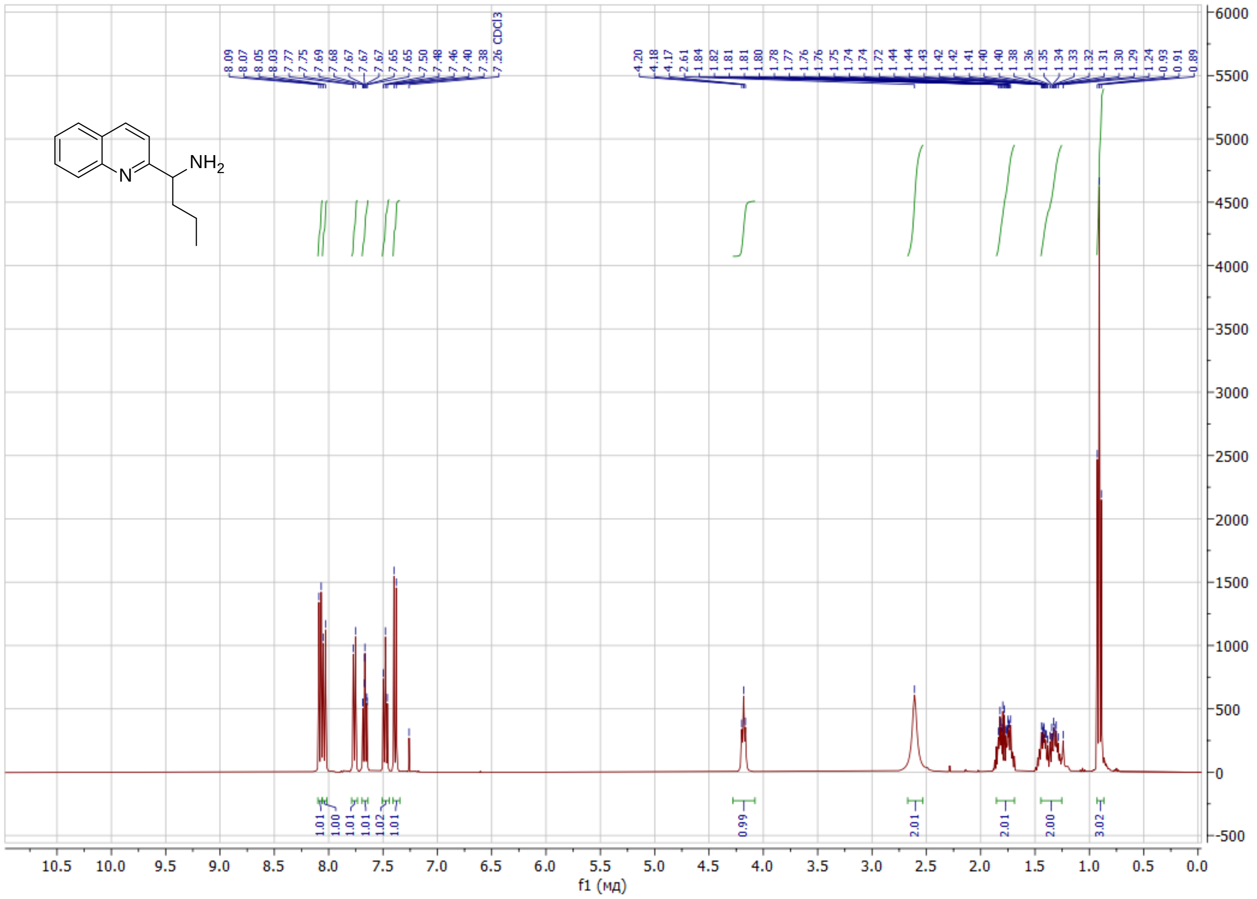

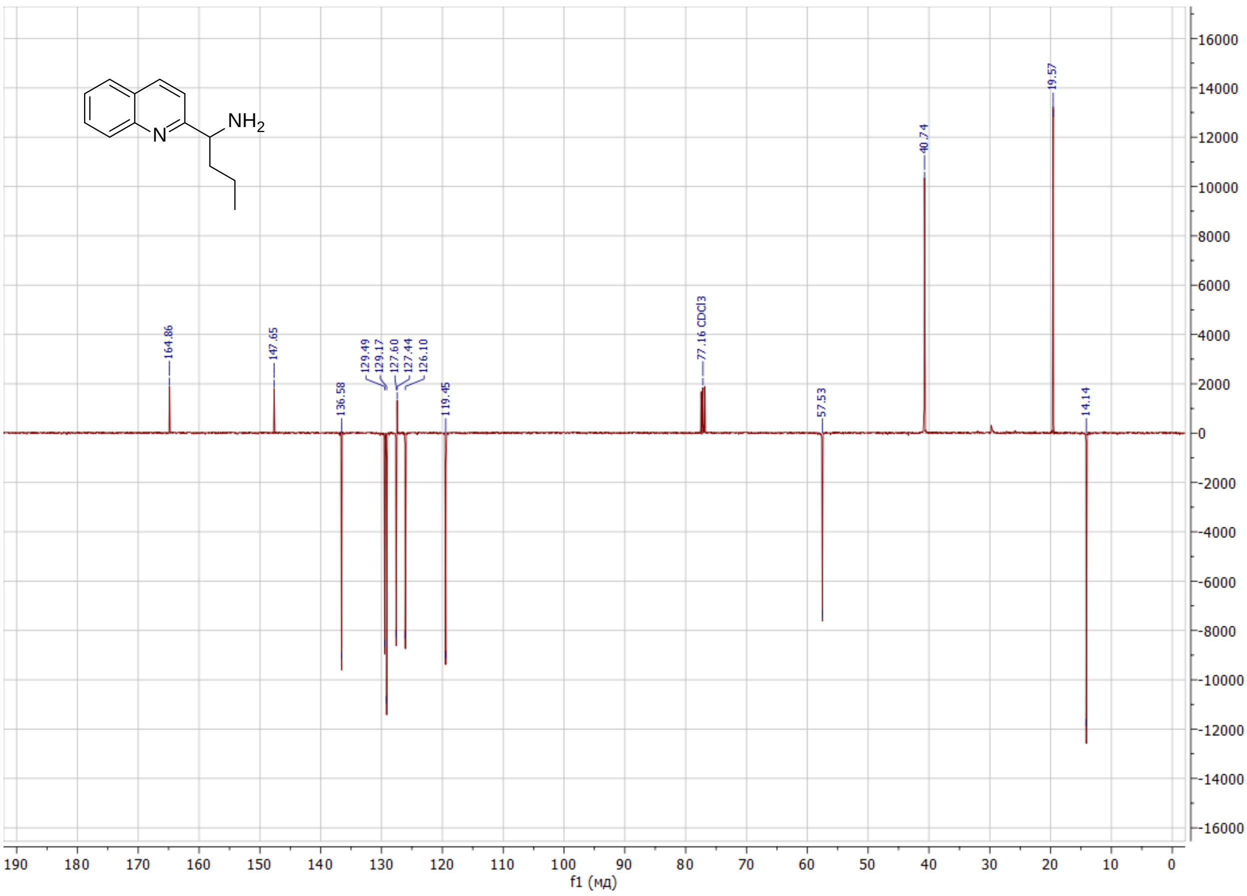

^{1}H (400 MHz) and ^{13}C (101 MHz) NMR spectra of **18c** in $CDCl_3$.

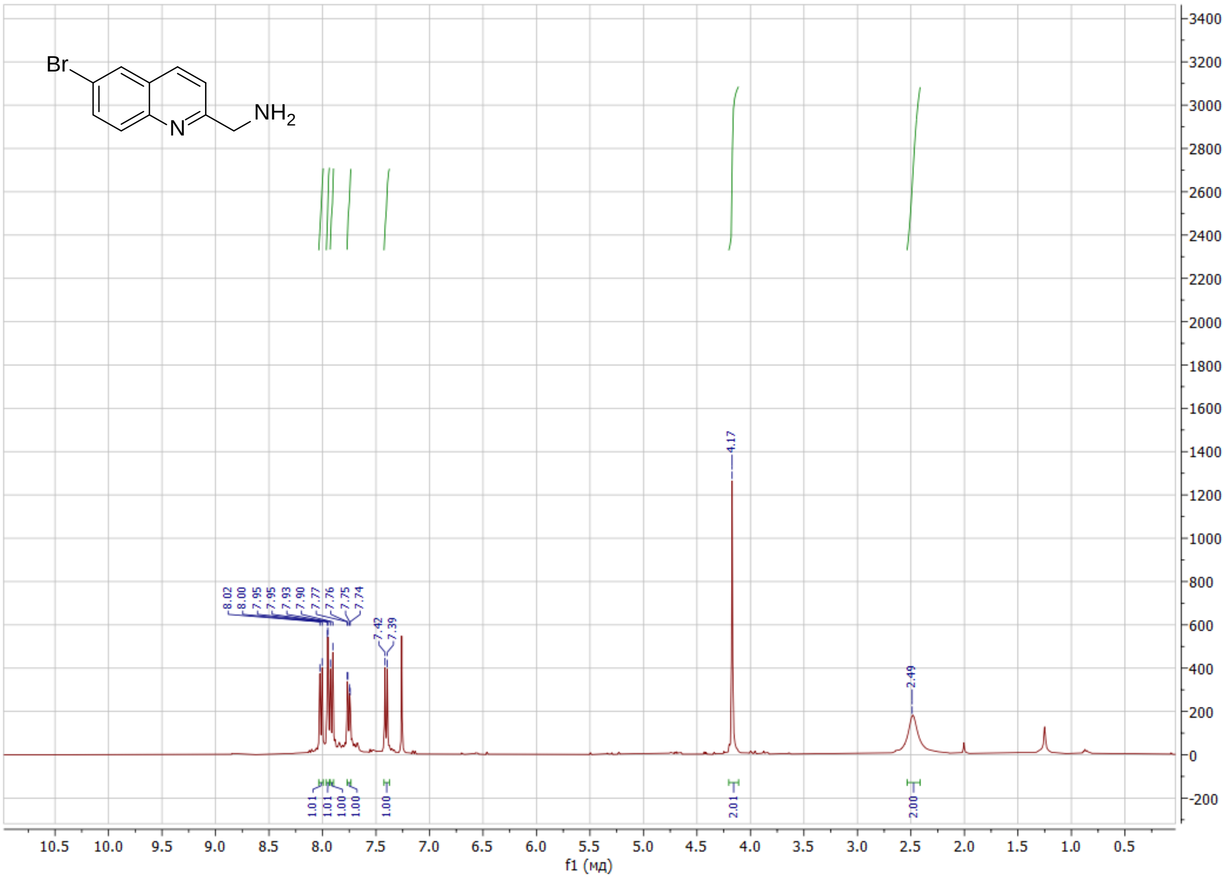

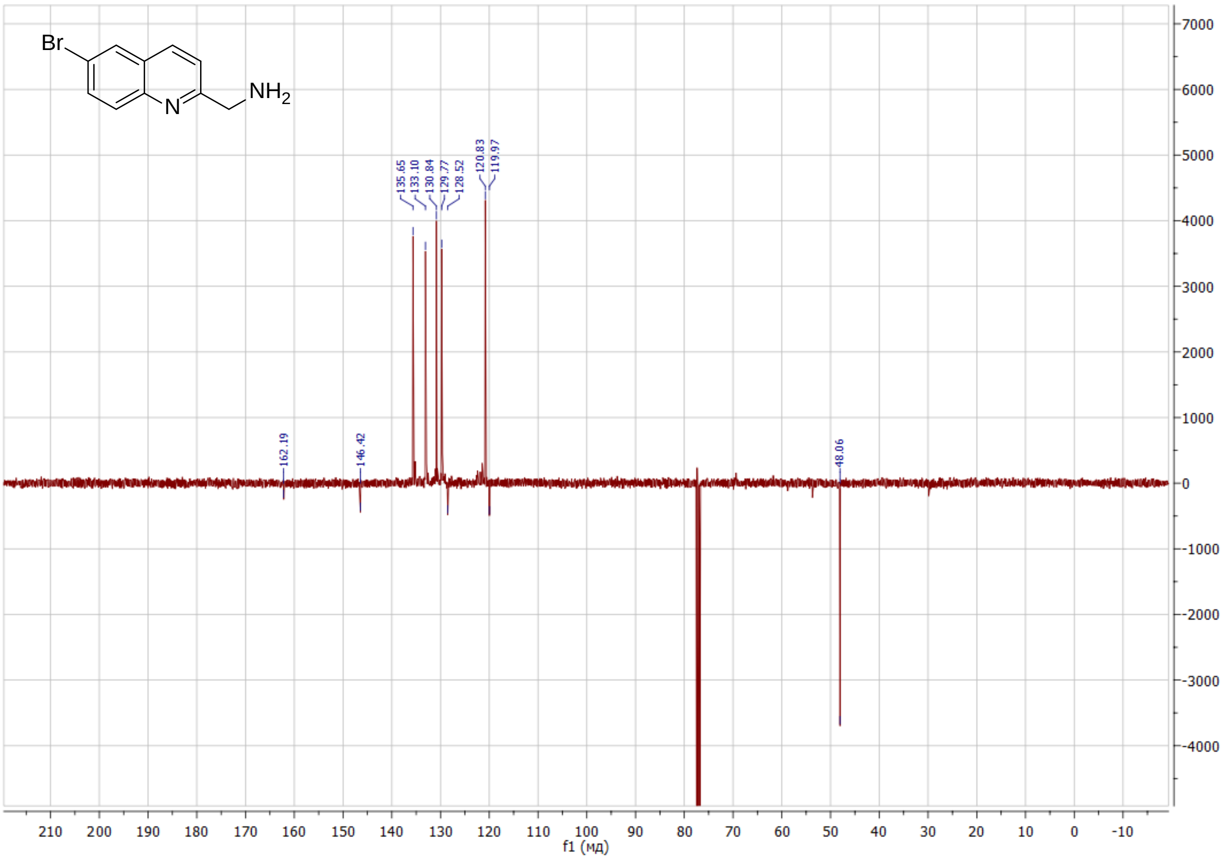

^{1}H (400 MHz) and ^{13}C (101 MHz) NMR spectra of **18d** in DMSO-d_6.

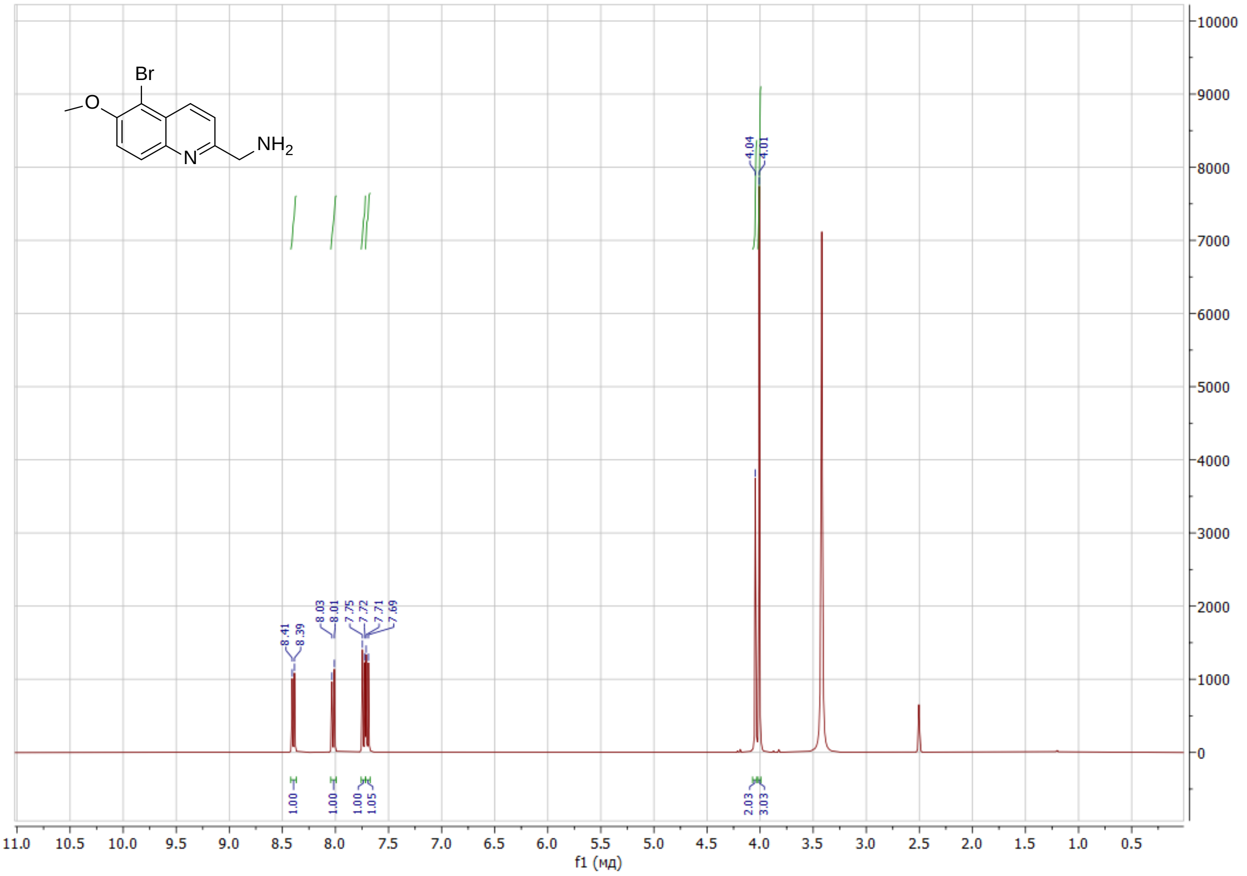

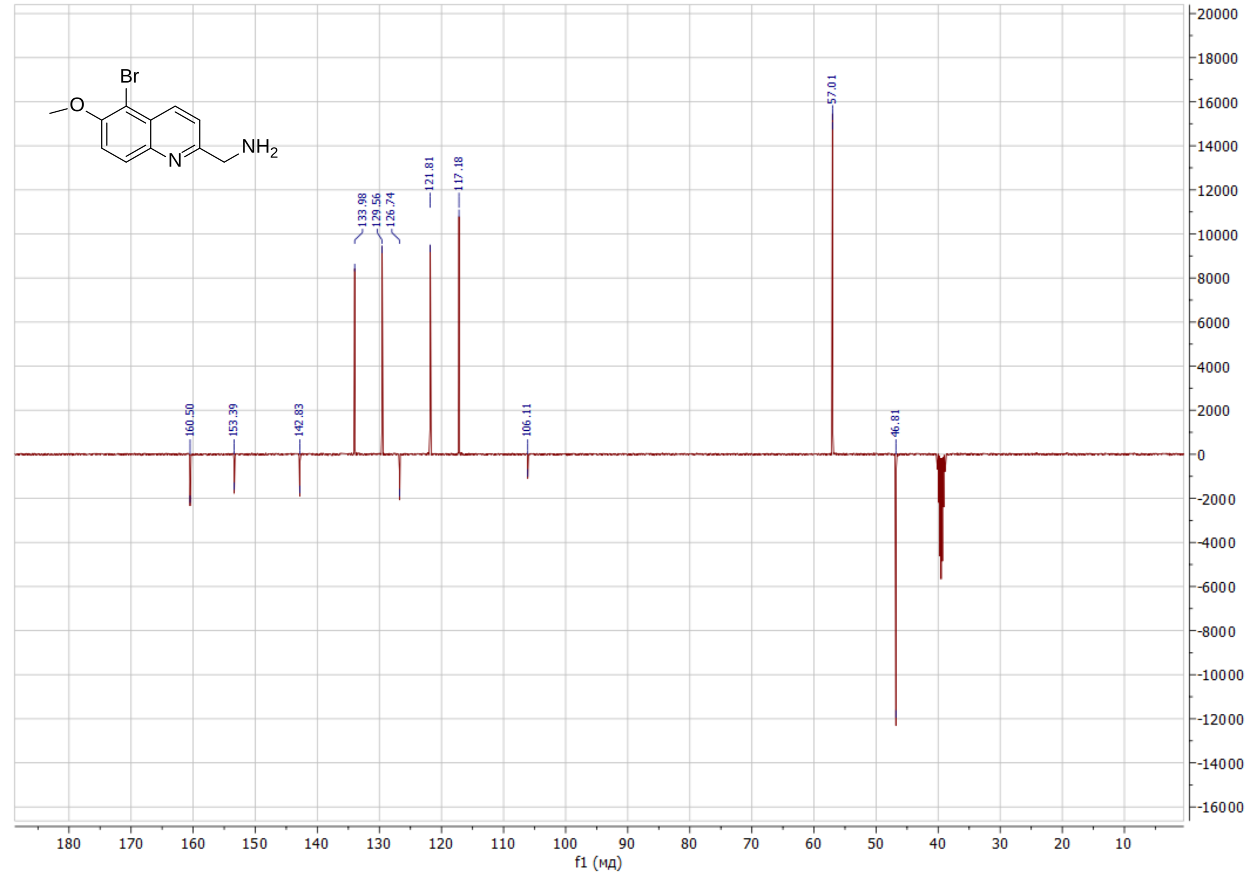

^{1}H (400 MHz) and ^{13}C (101 MHz) NMR spectra of **16e** in DMSO-d_6.

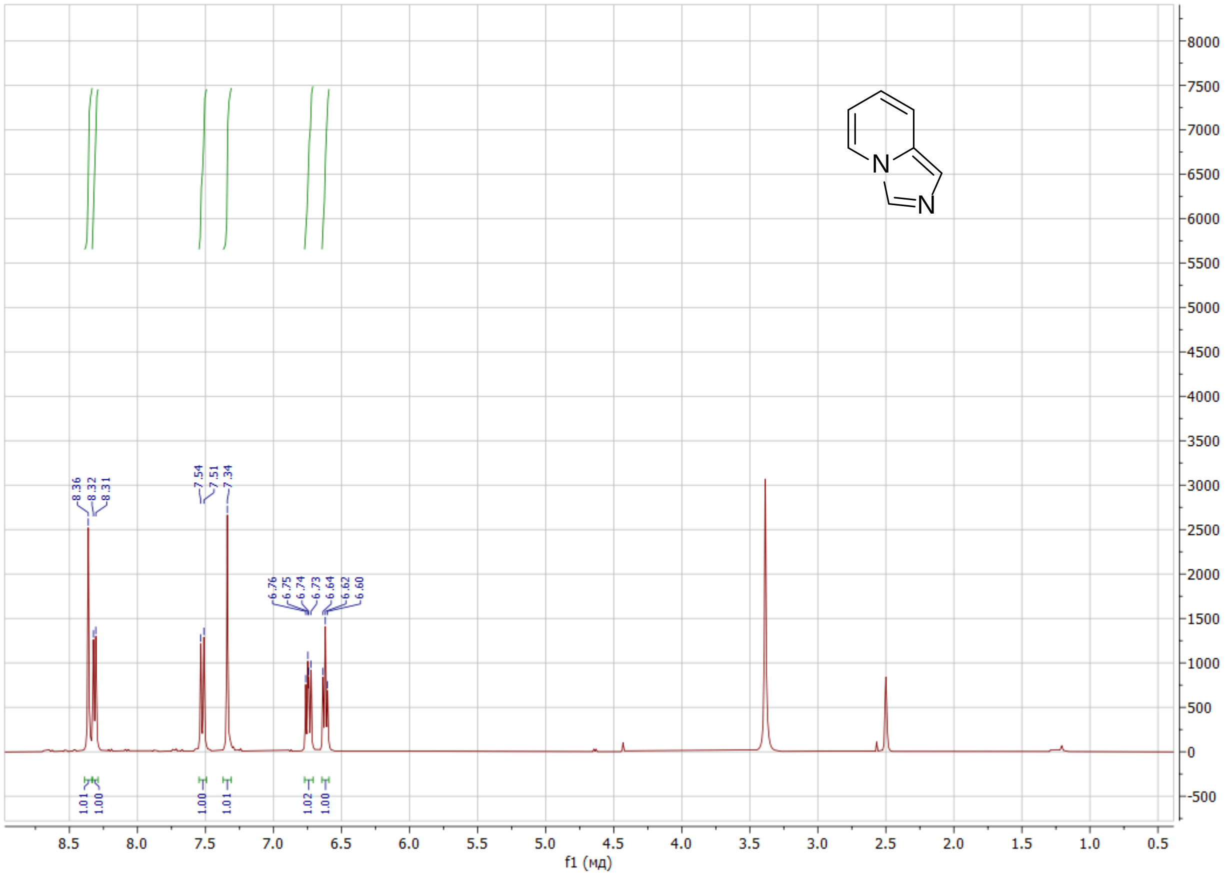

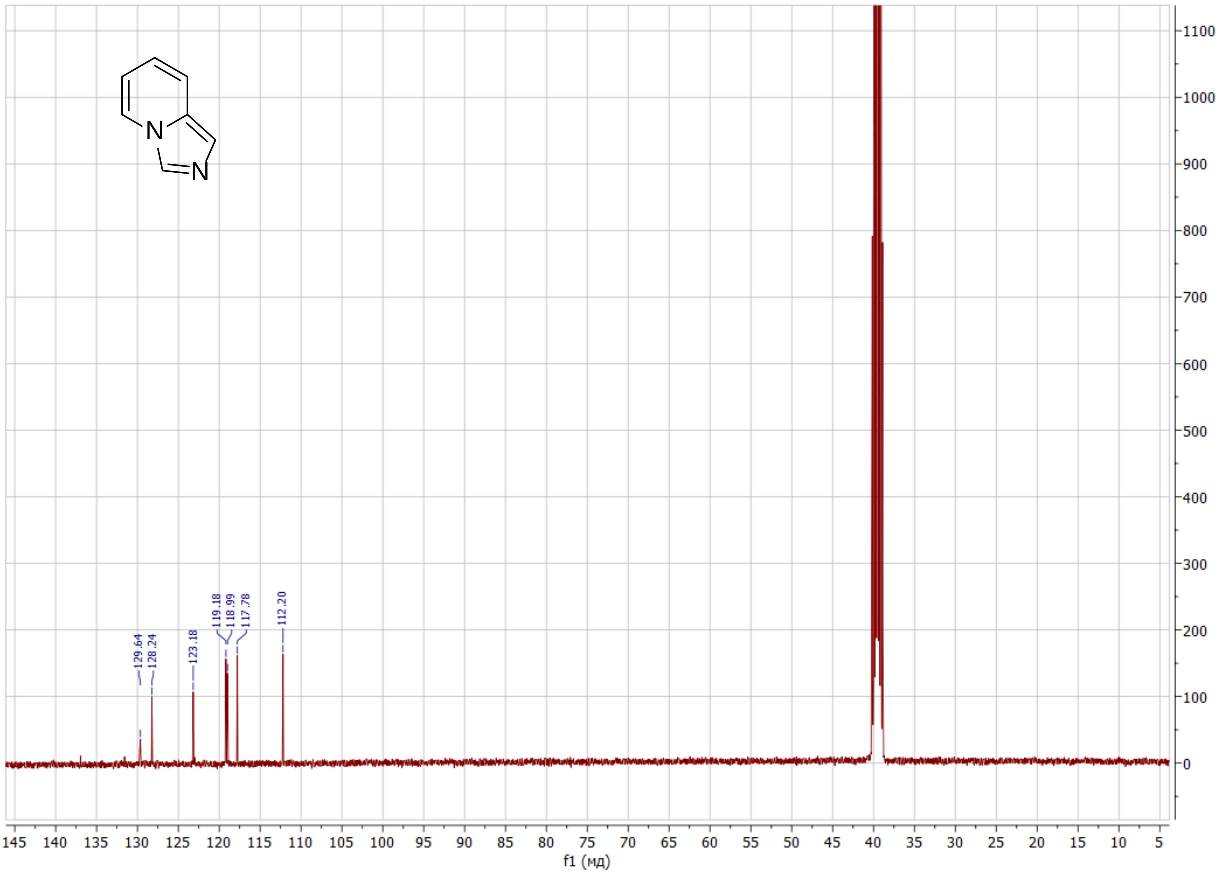

^{1}H (400 MHz) and ^{13}C (101 MHz) NMR spectra of **16a** in DMSO-d_6.

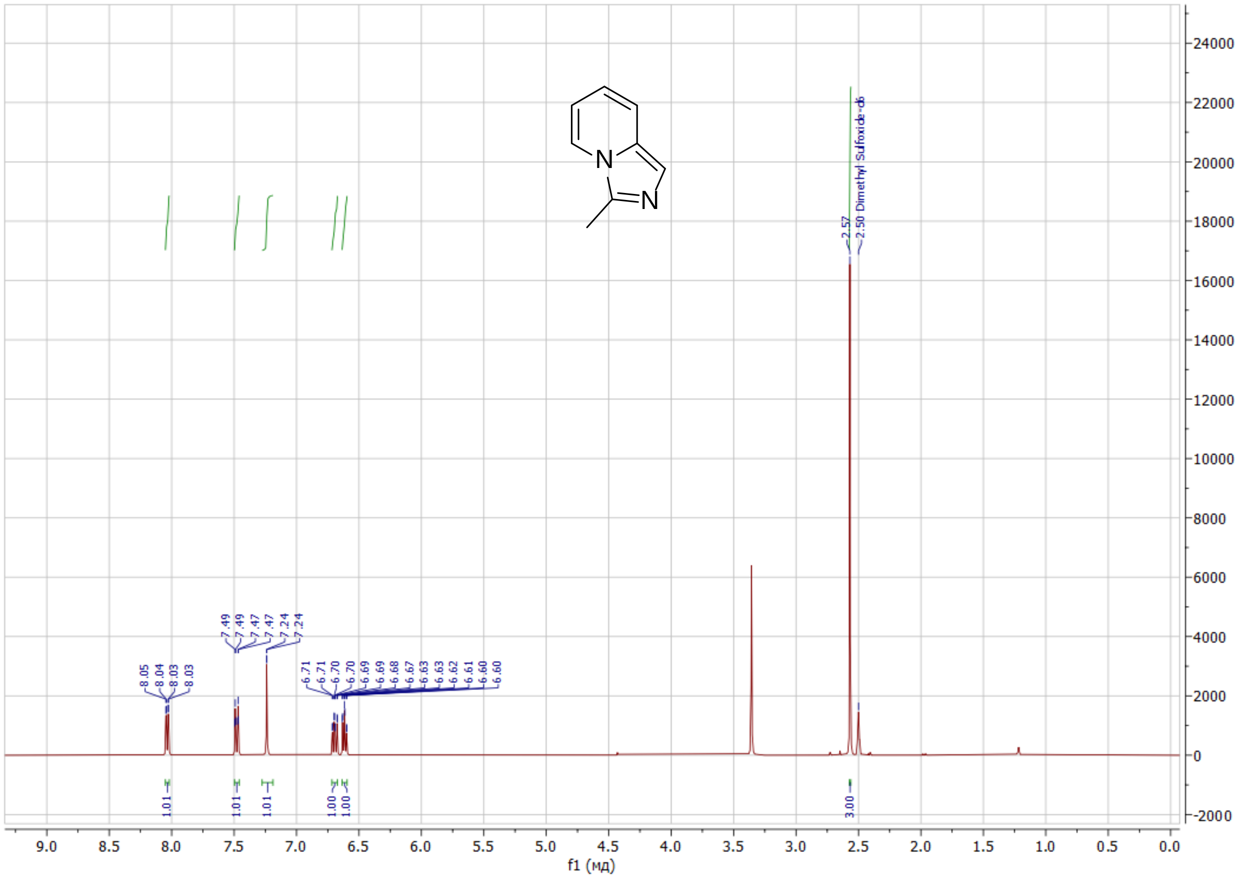

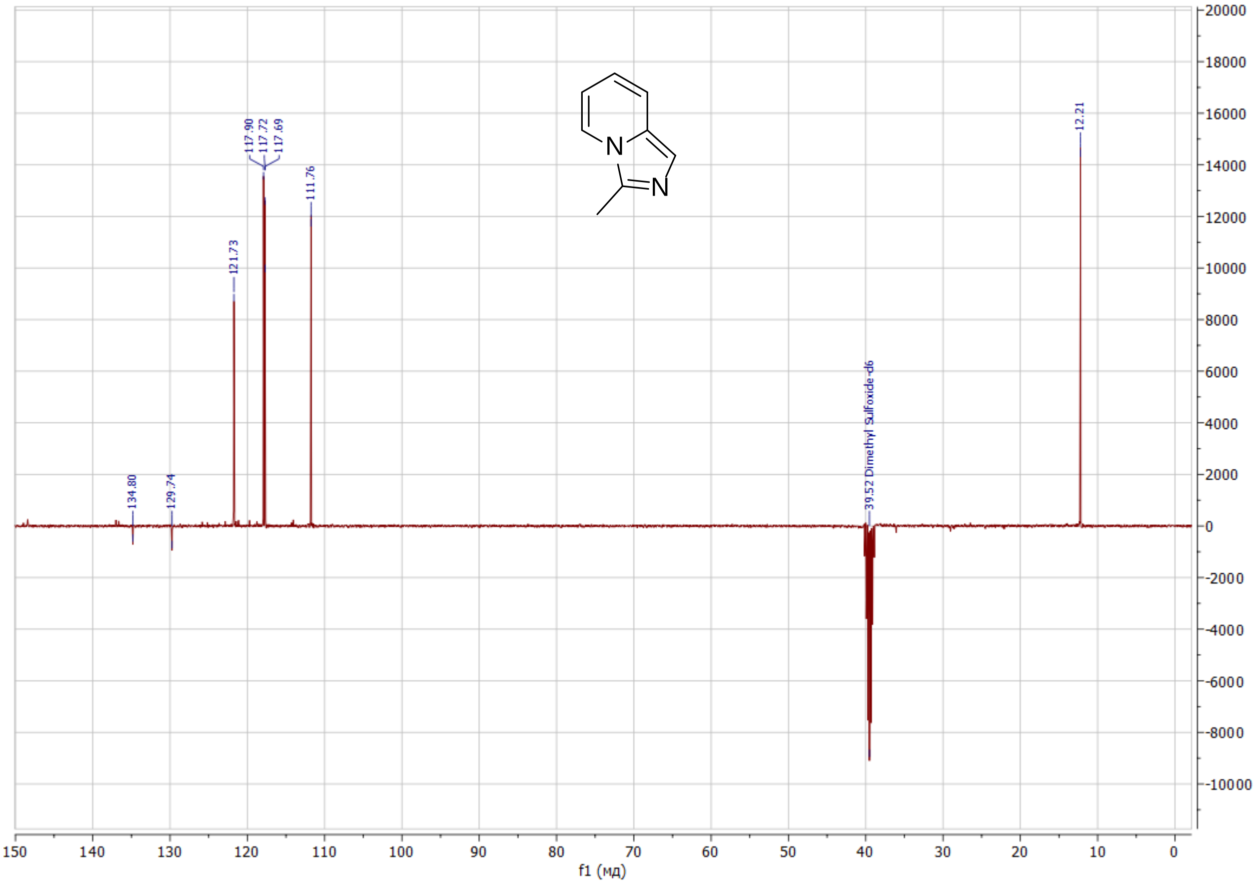

^{1}H (400 MHz) and ^{13}C (101 MHz) NMR spectra of **16b** in DMSO-d_6.

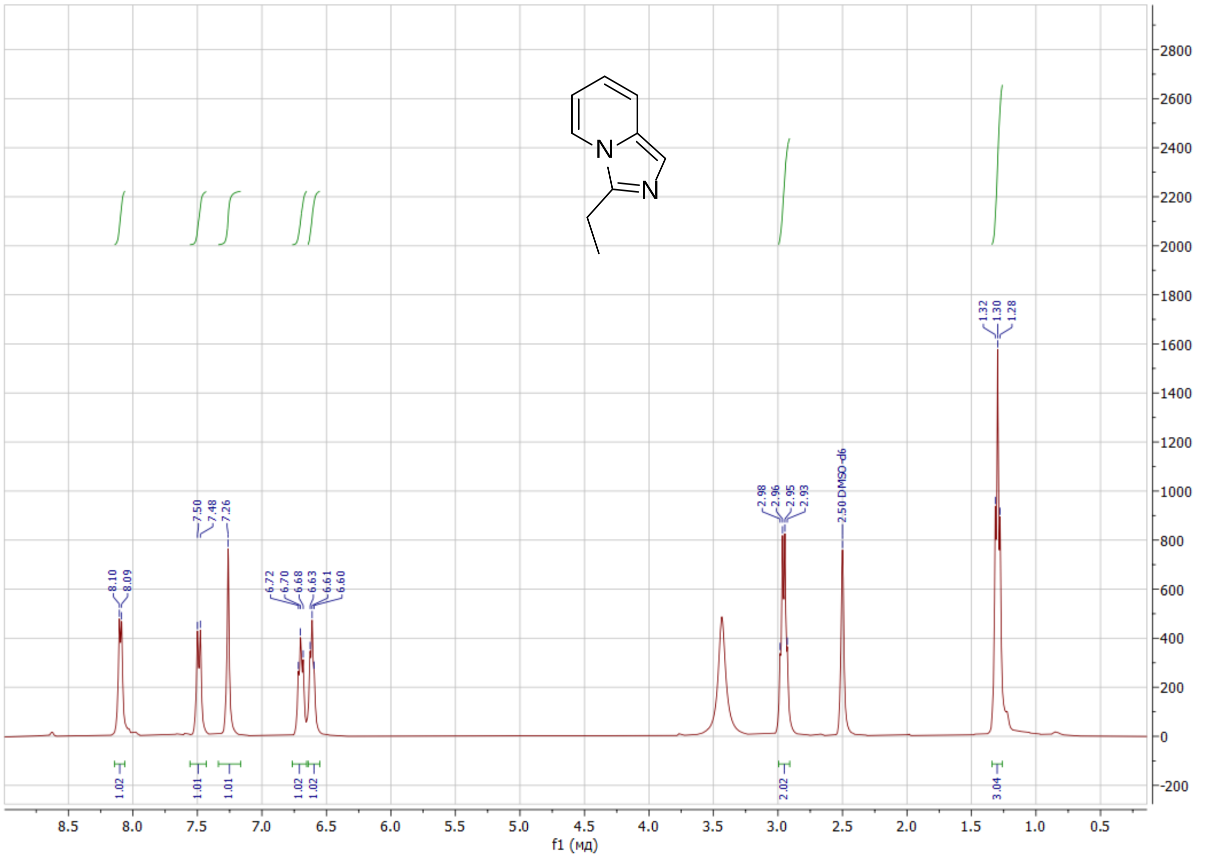

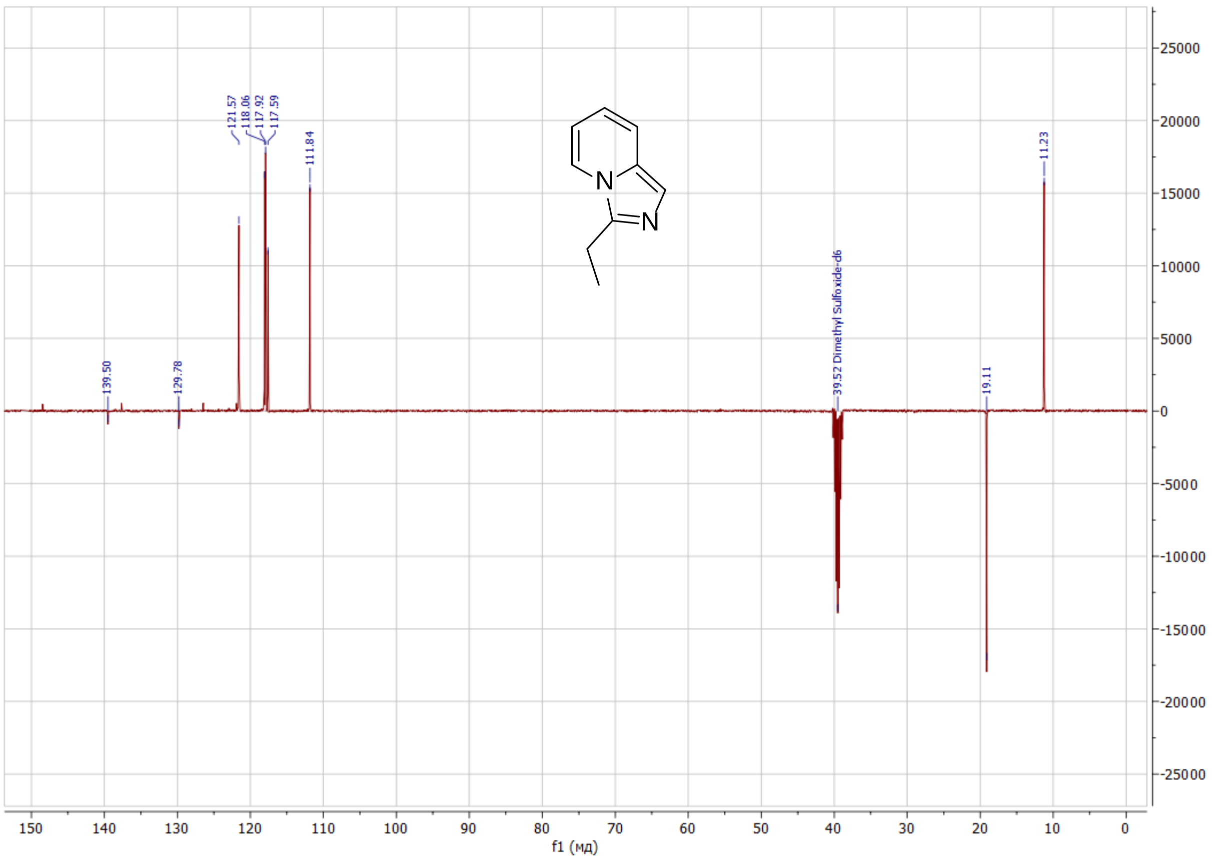

^{1}H (400 MHz) and ^{13}C (101 MHz) NMR spectra of **16c** in DMSO-d_6.

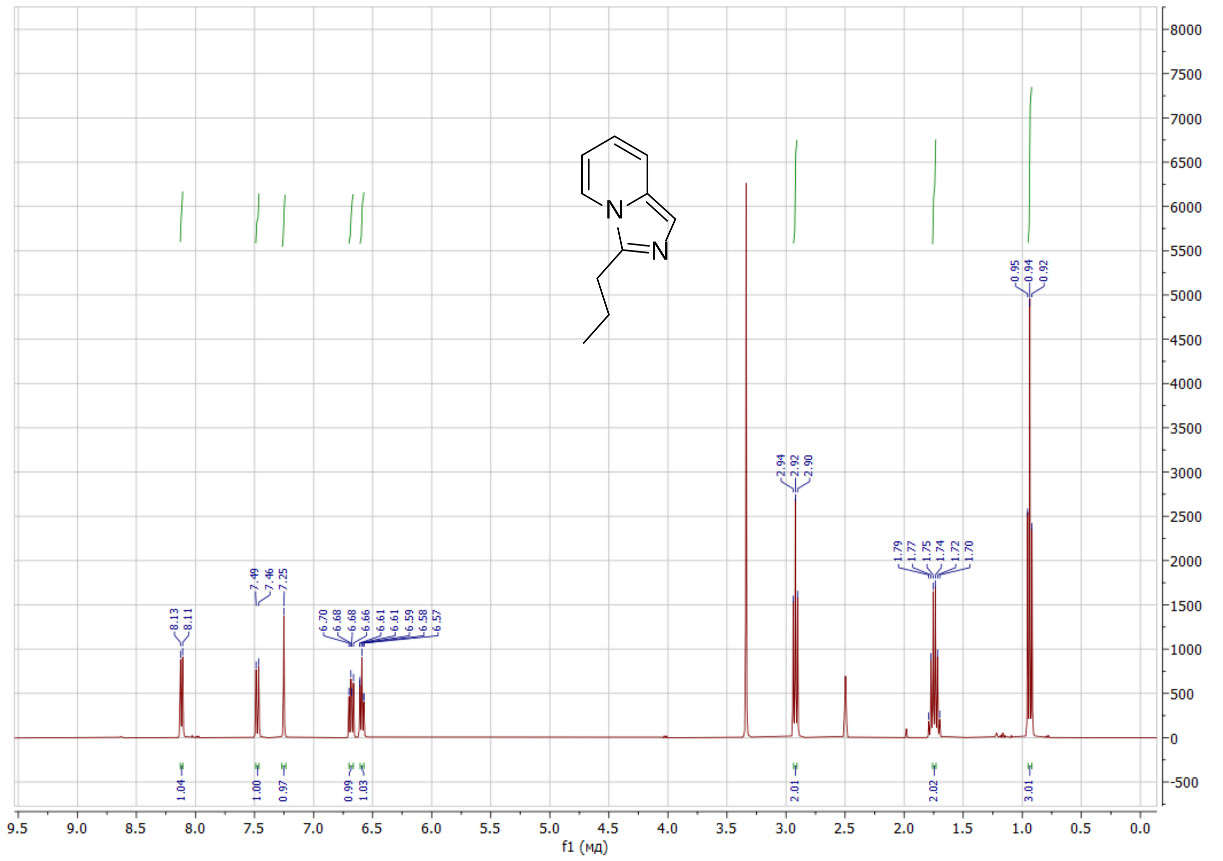

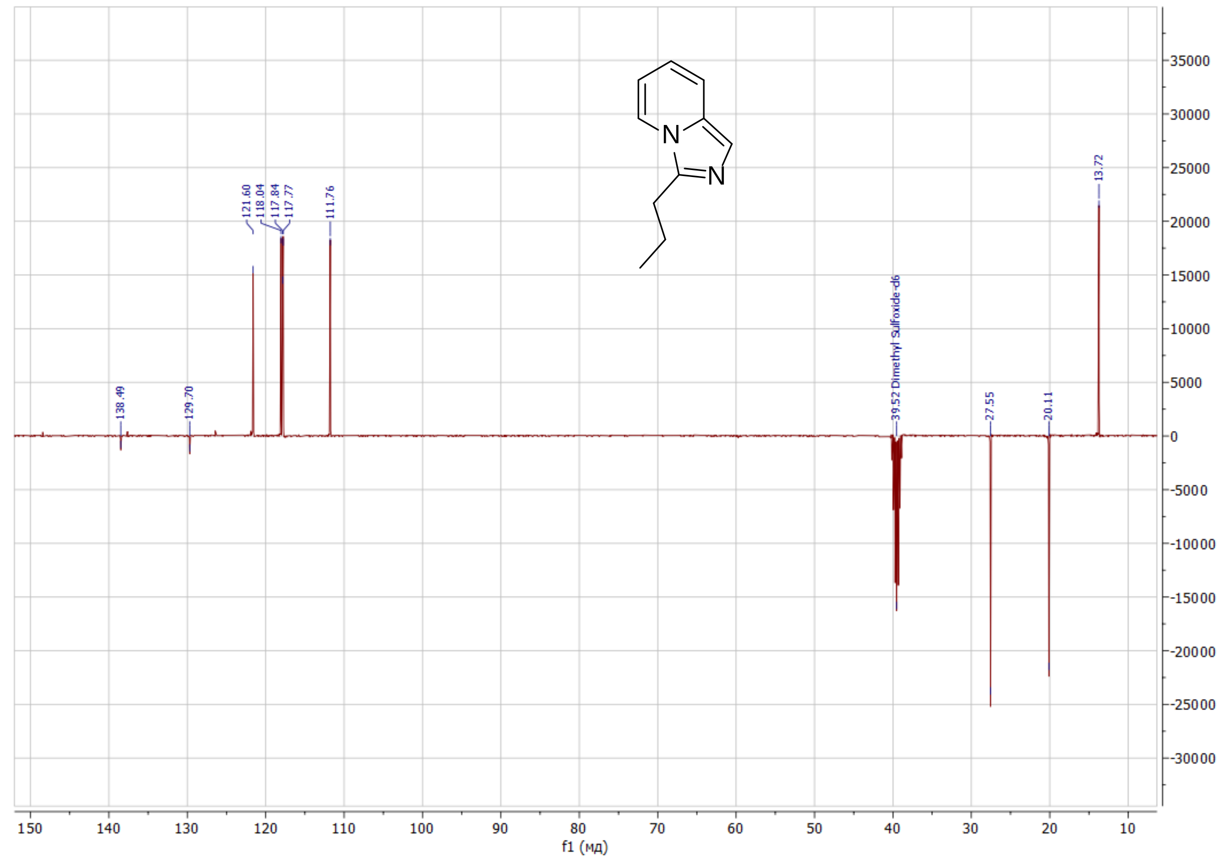

^{1}H (400 MHz) and ^{13}C (101 MHz) NMR spectra of **16d** in DMSO-d_6.

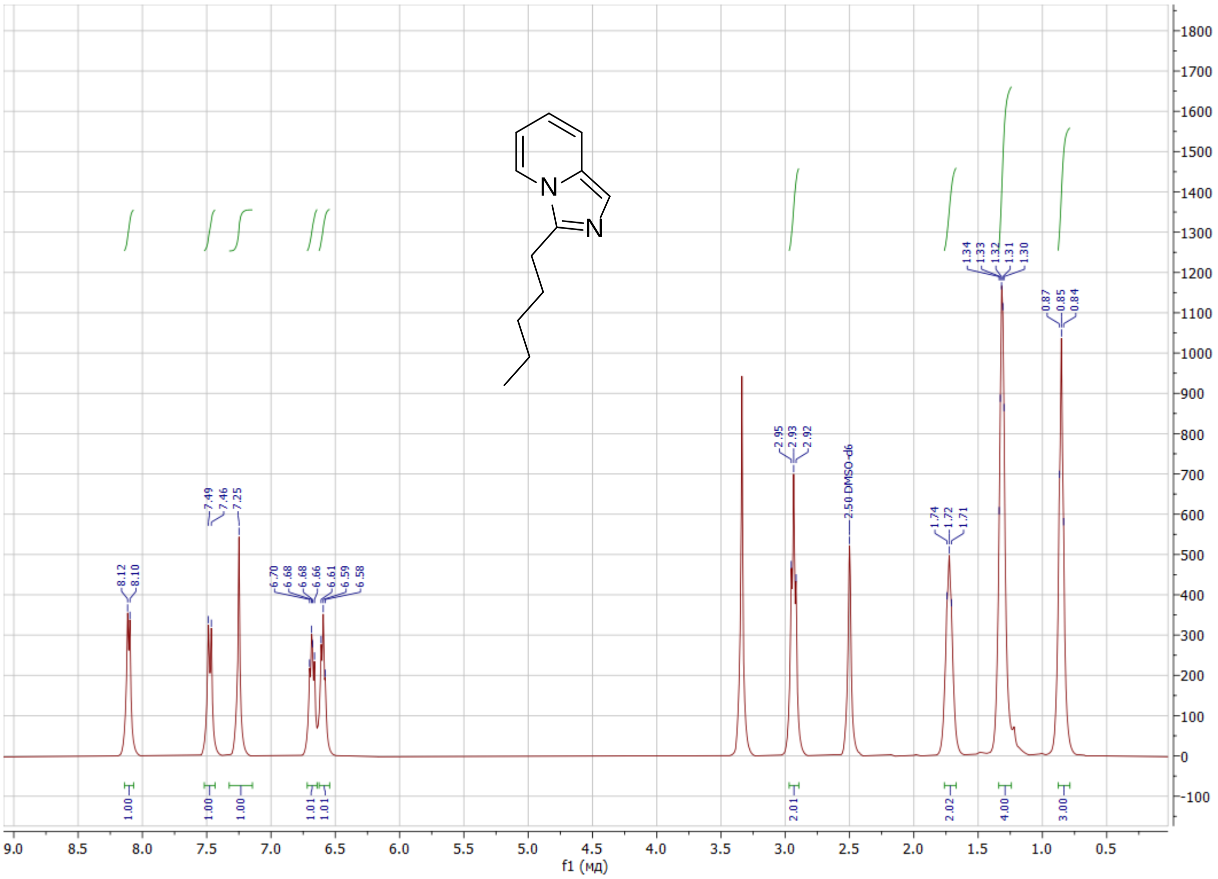

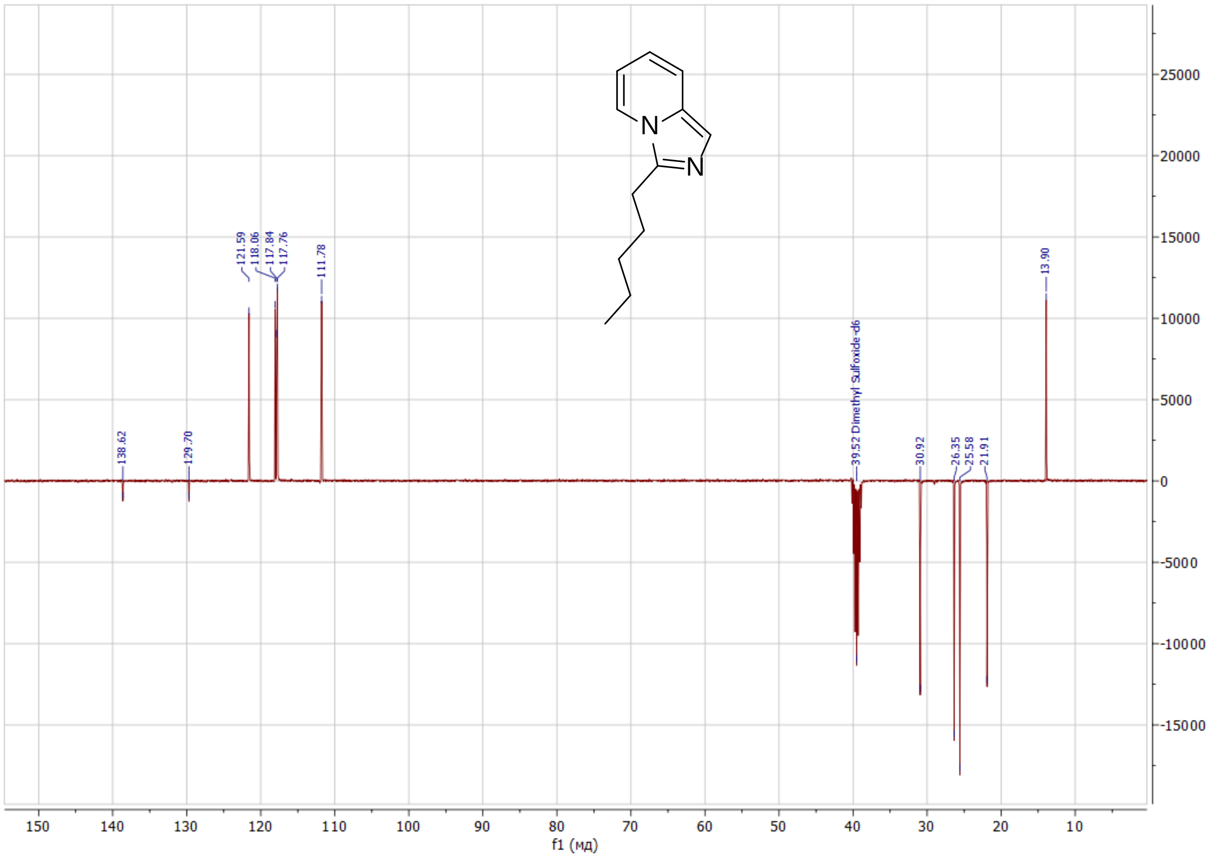

^{1}H (400 MHz) and ^{13}C (101 MHz) NMR spectra of **16f** in DMSO-d_6.

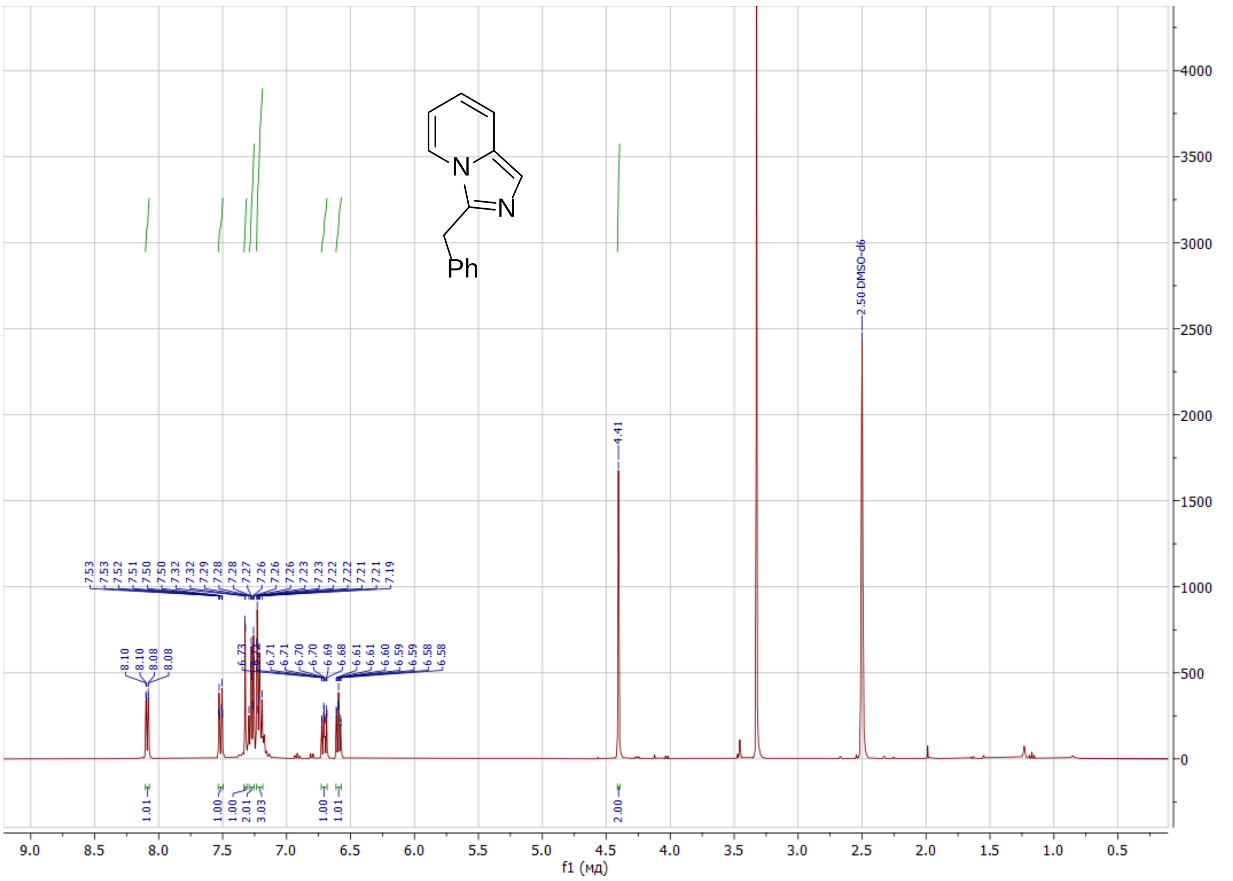

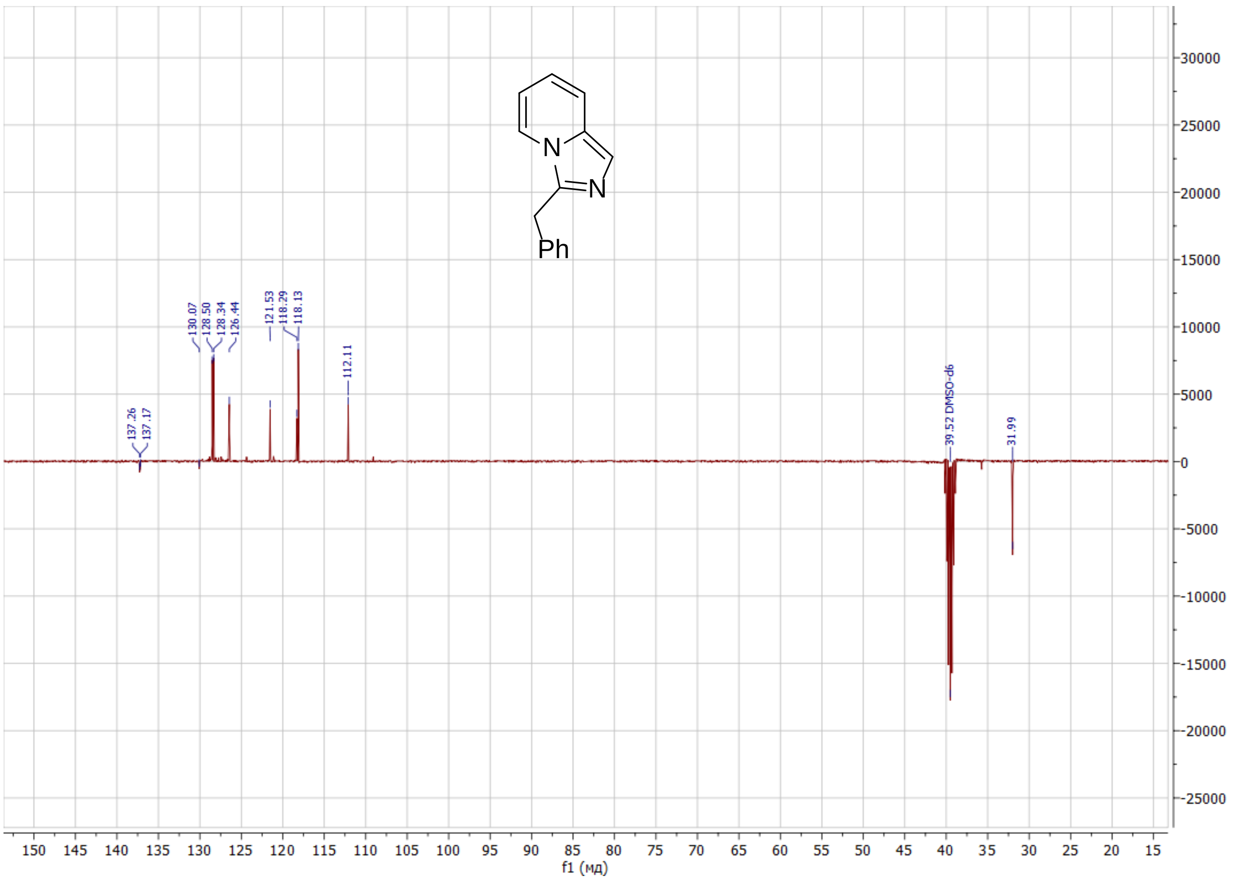

^{1}H (400 MHz) and ^{13}C (101 MHz) NMR spectra of **16g** in DMSO-d_6.

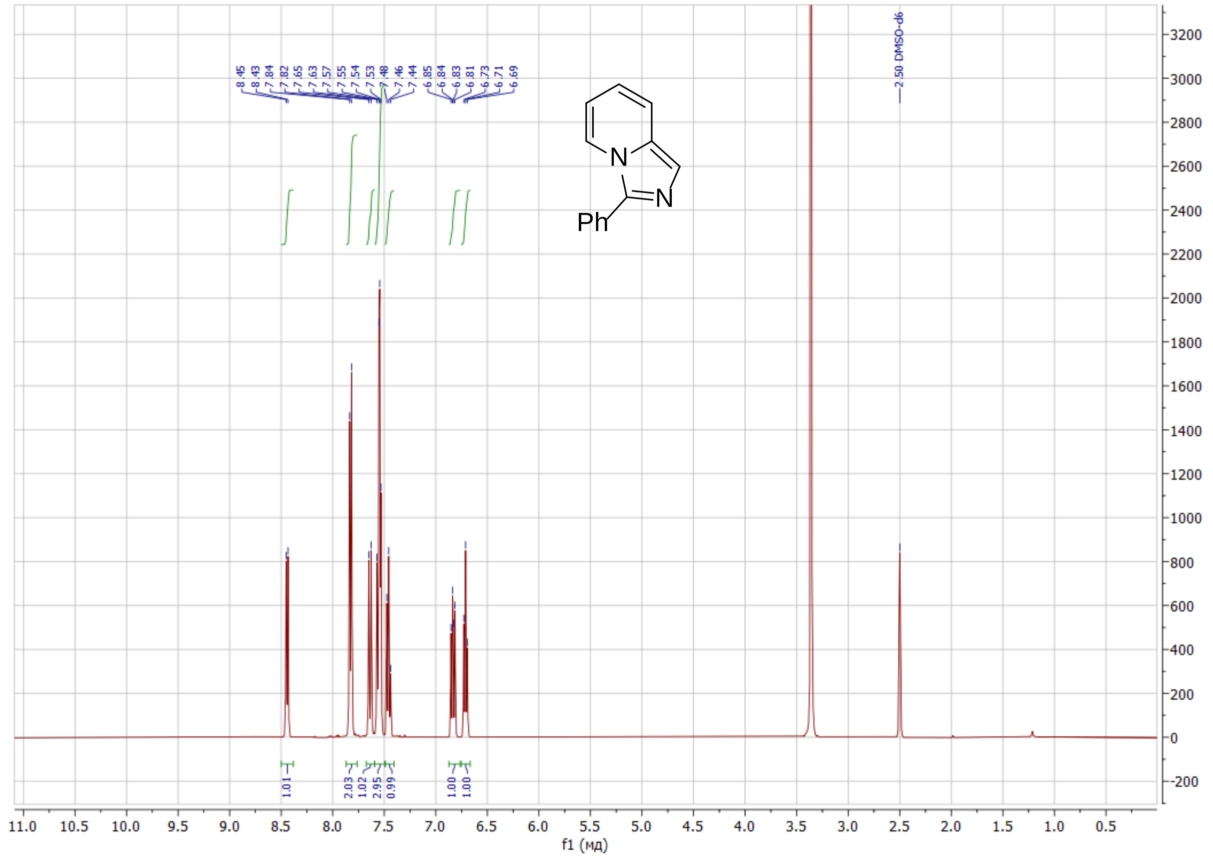

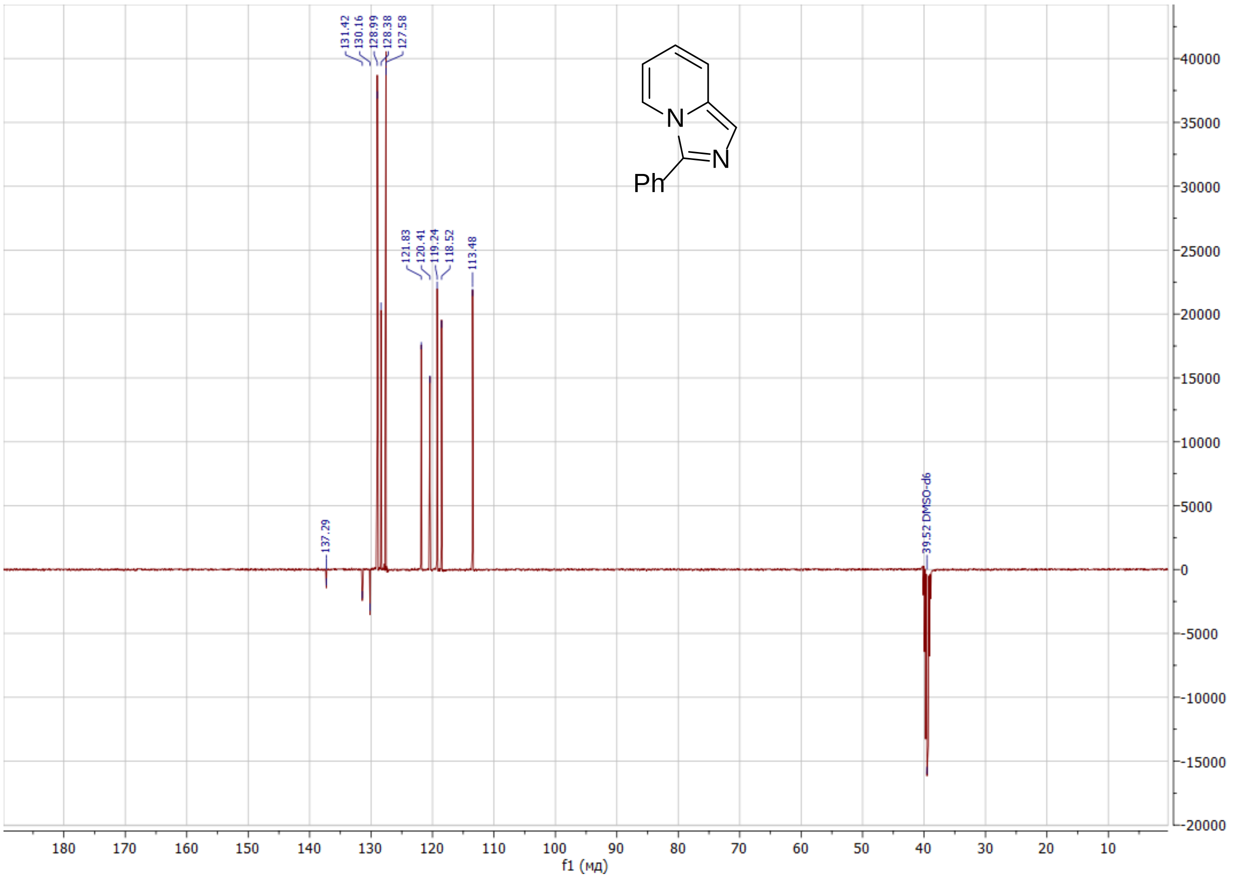

^{1}H (400 MHz) and ^{13}C (101 MHz) NMR spectra of **19ae** in DMSO-d_6.

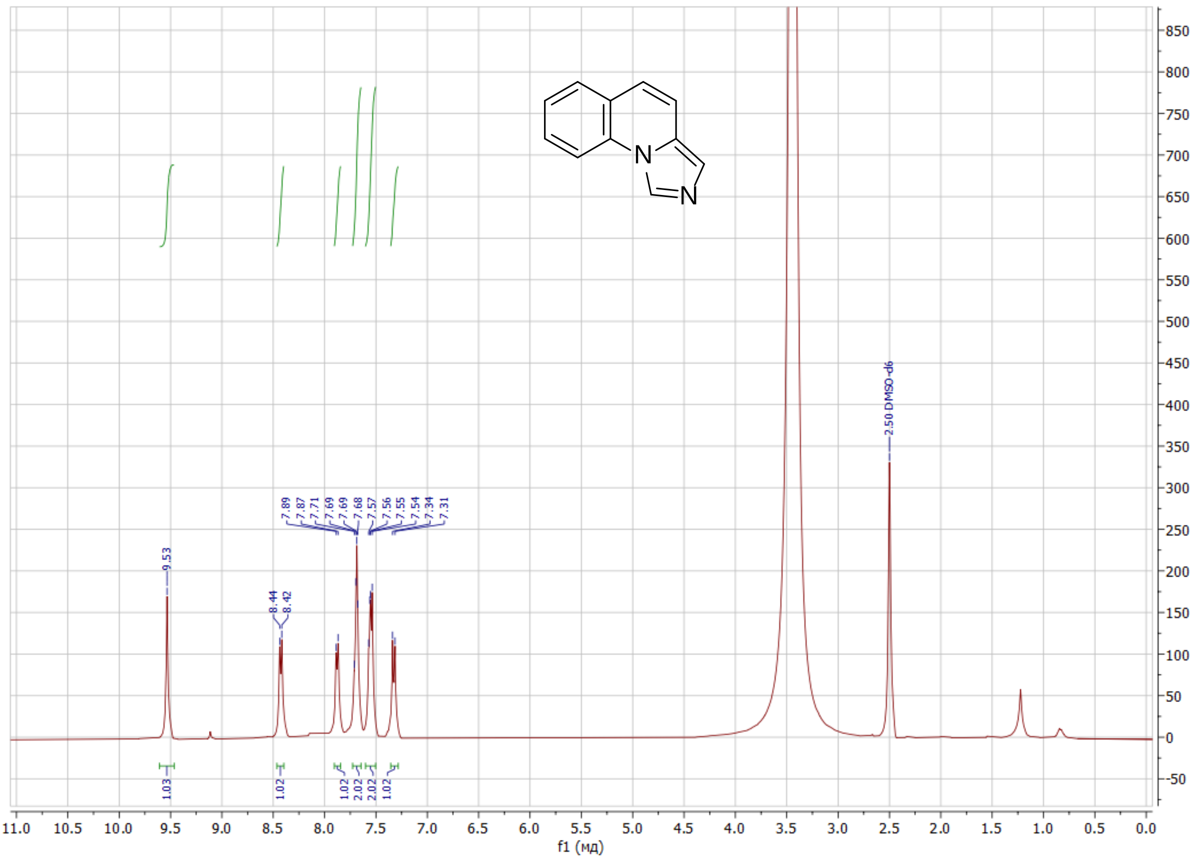

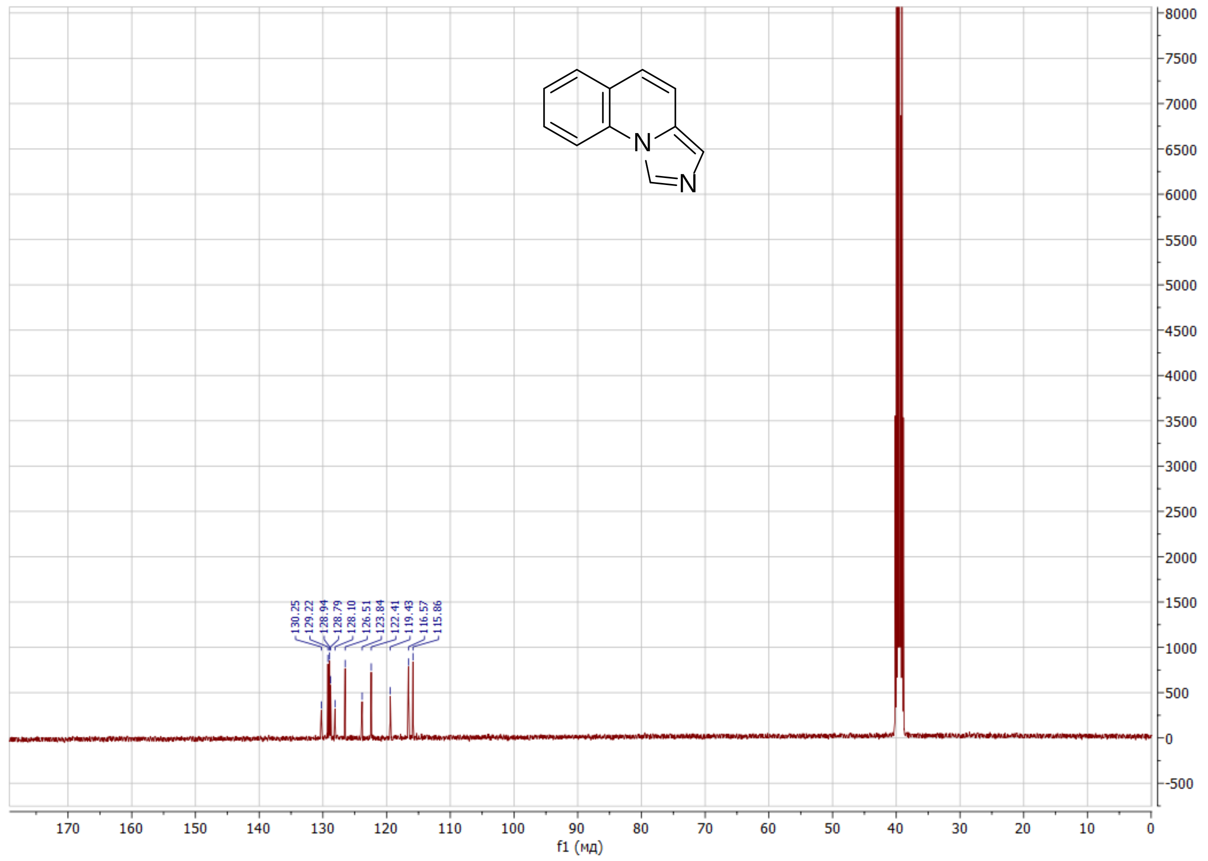

^{1}H (400 MHz) and ^{13}C (101 MHz) NMR spectra of **19aa** in $CDCl_3$.

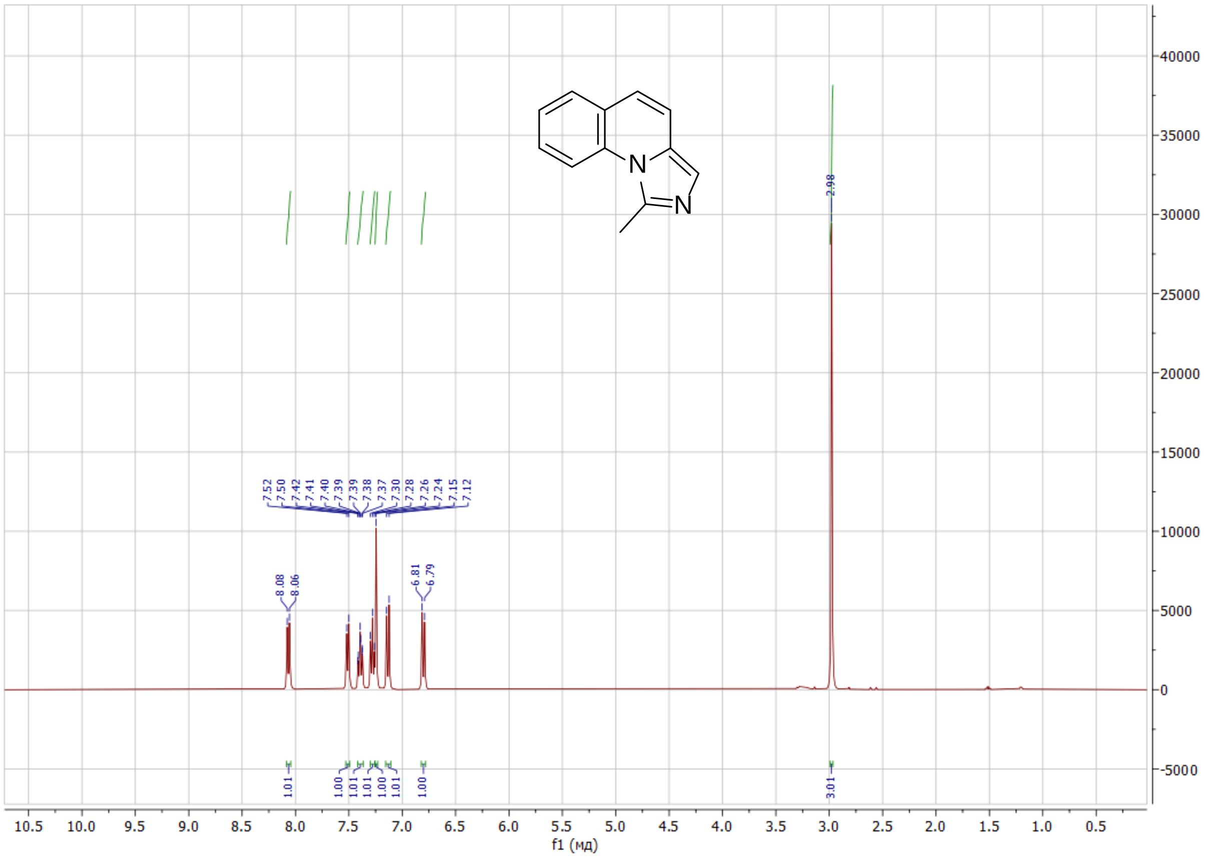

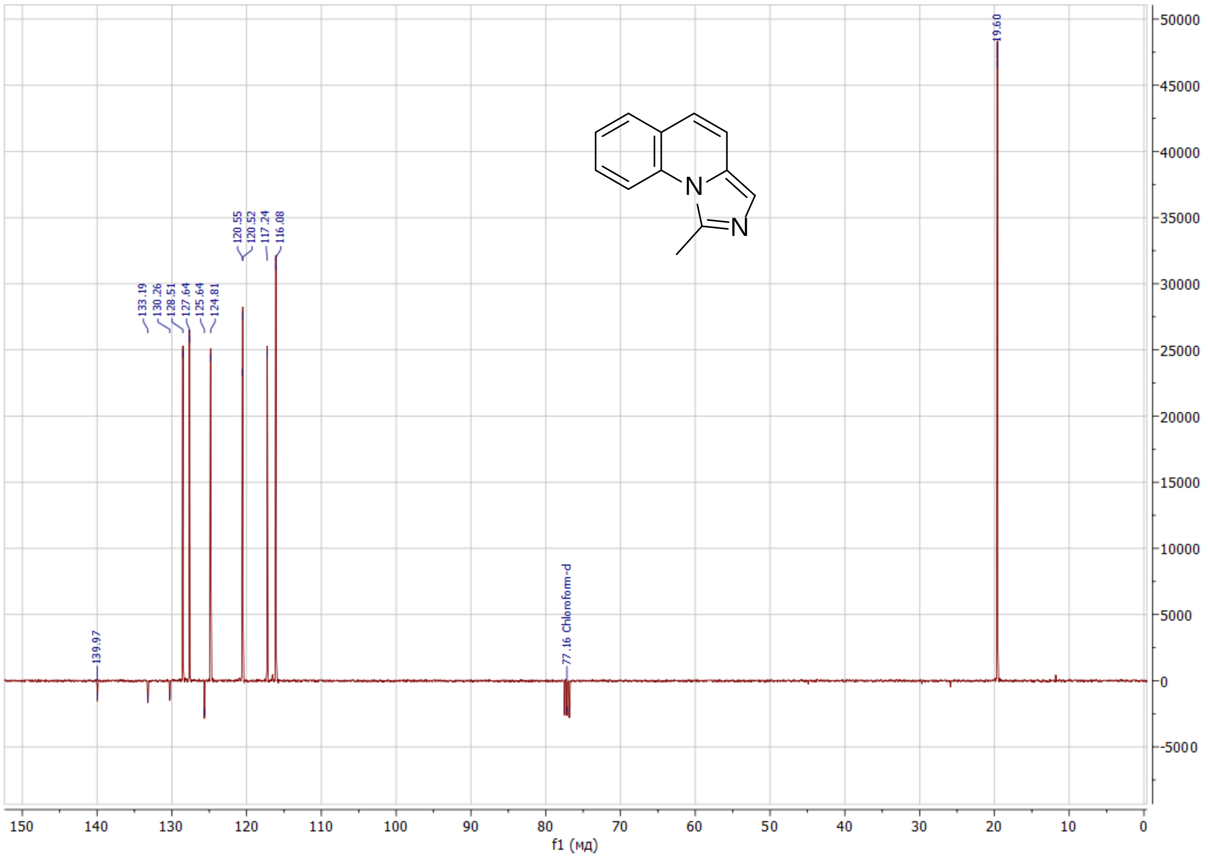

^{1}H (400 MHz) and ^{13}C (101 MHz) NMR spectra of **19ab** in $CDCl_3$.

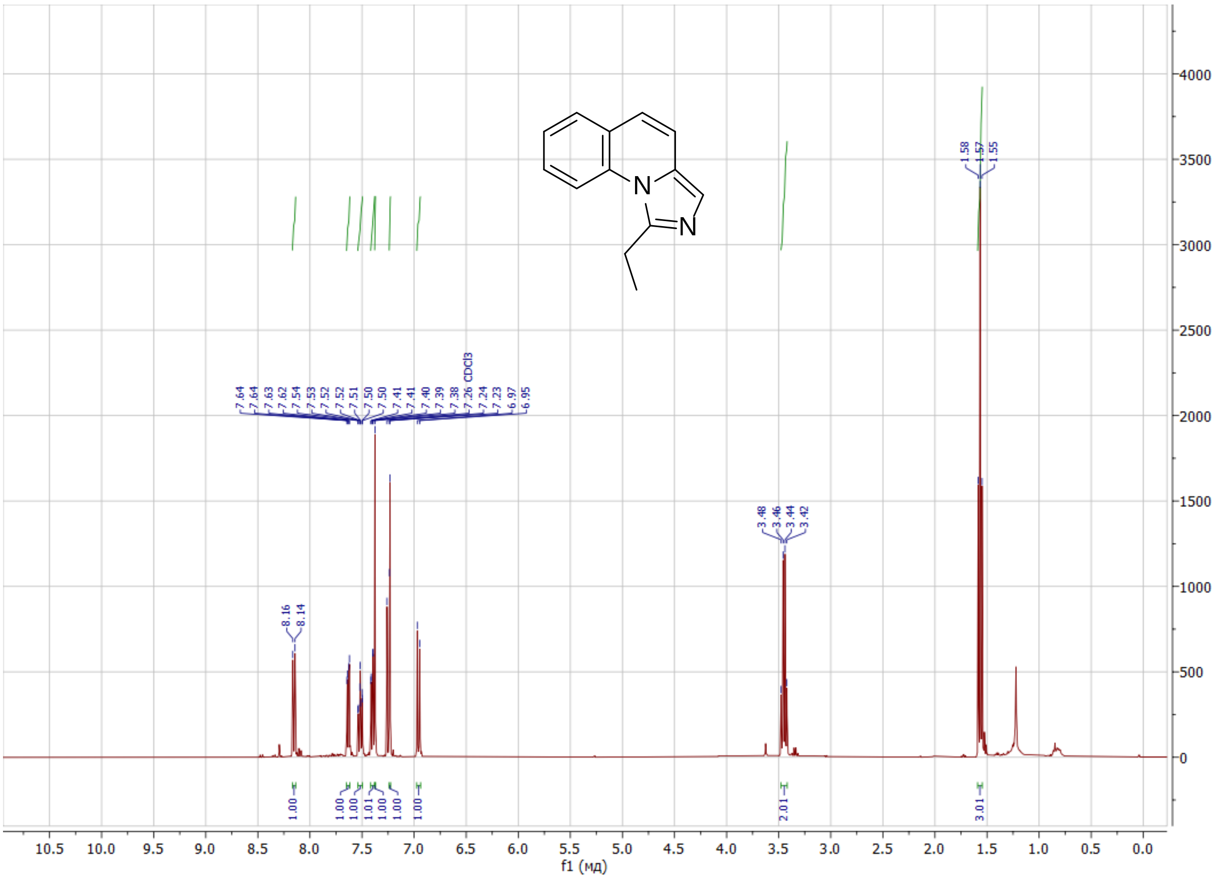

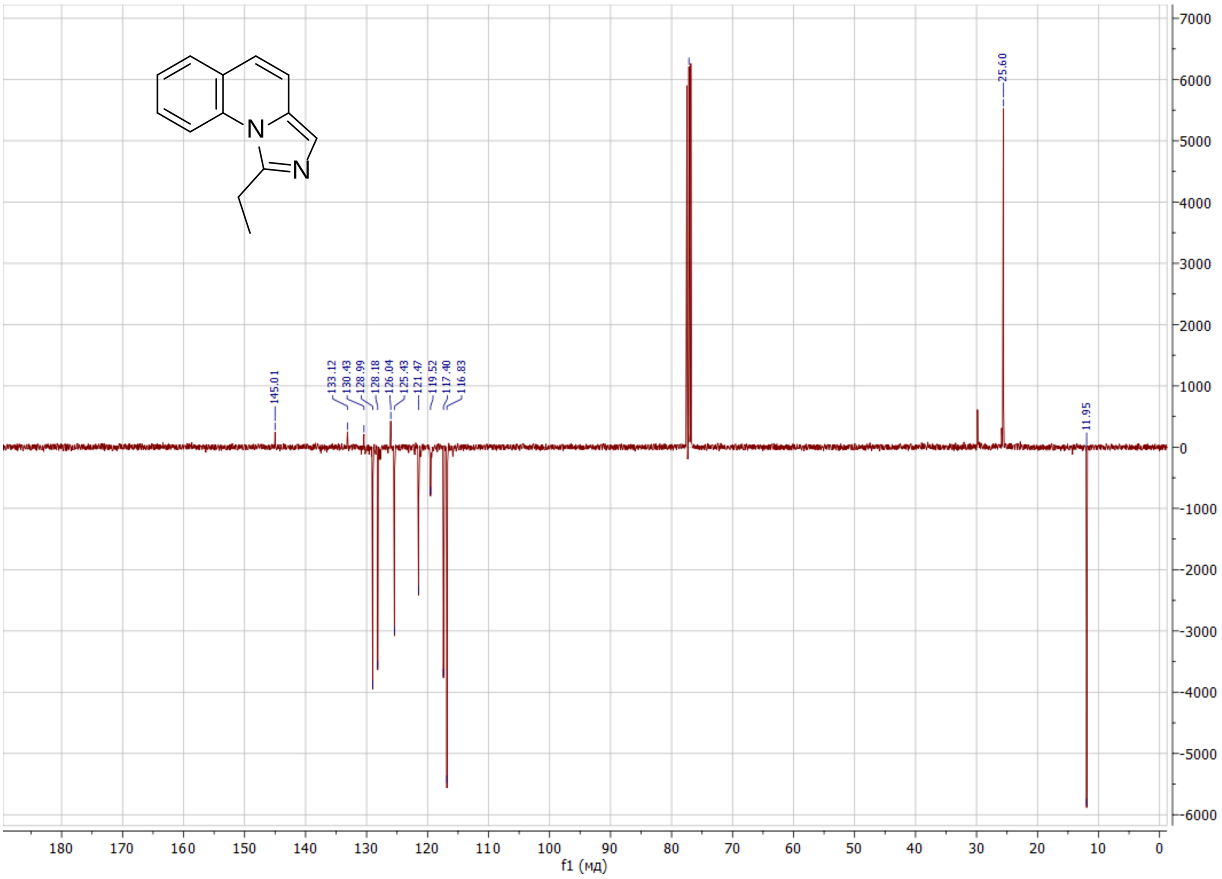

^{1}H (400 MHz) and ^{13}C (101 MHz) NMR spectra of **19ac** in DMSO-d_6.

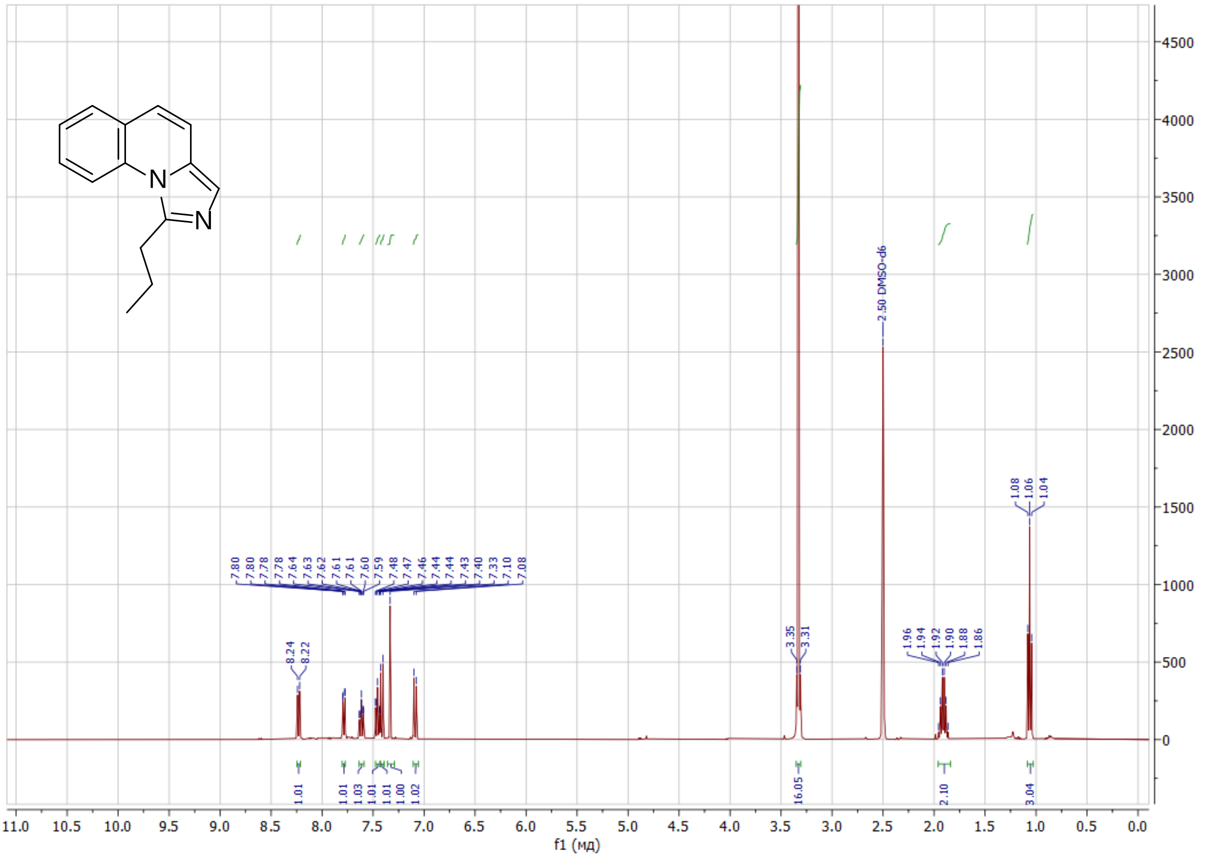

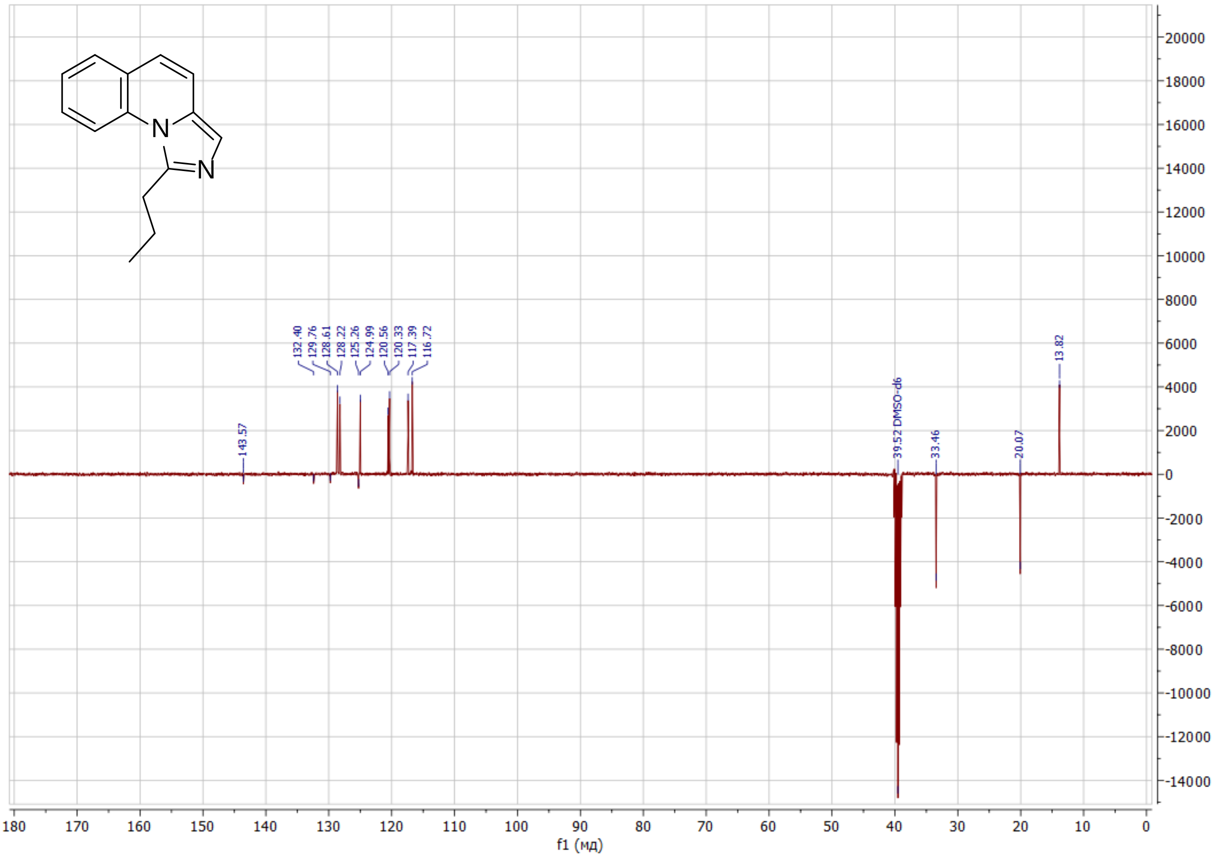

^{1}H (400 MHz) and ^{13}C (101 MHz) NMR spectra of **19ag** in DMSO-d_6.

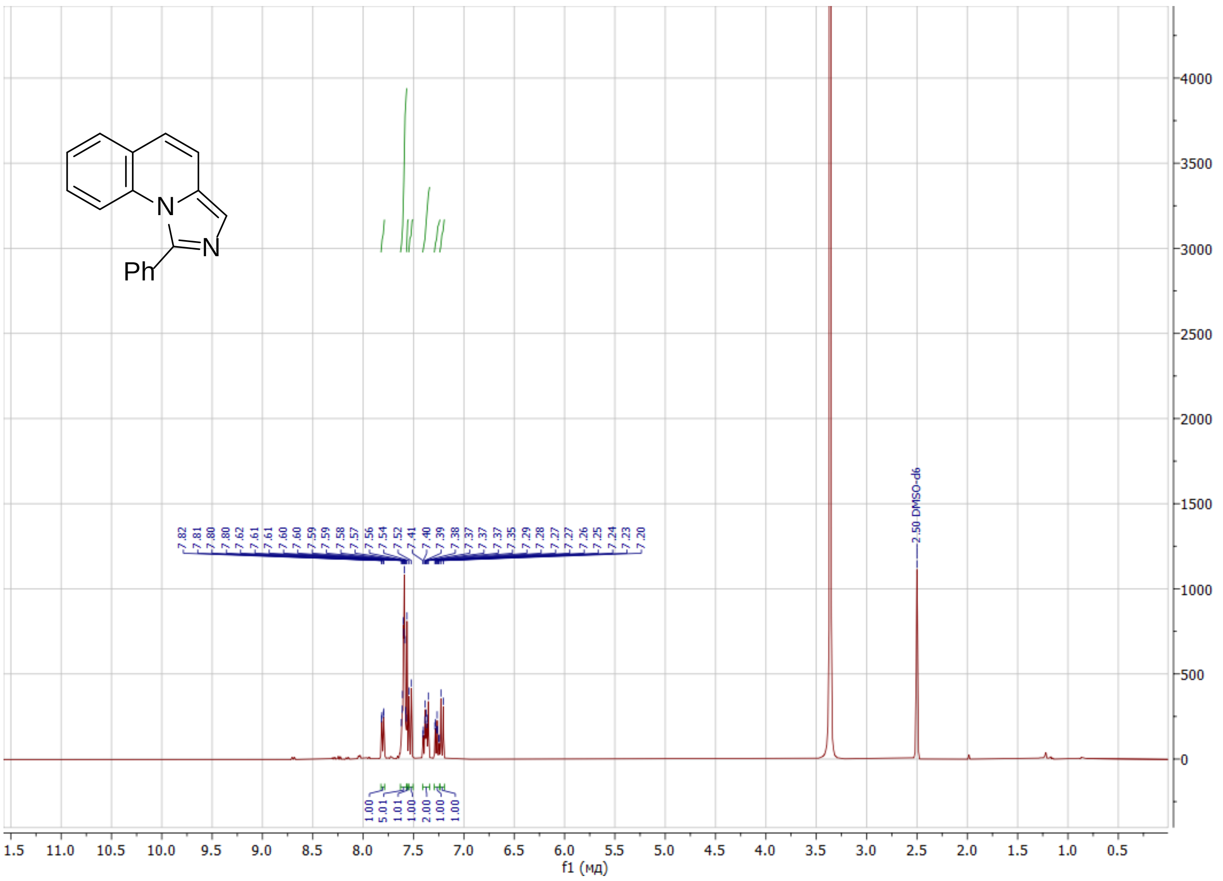

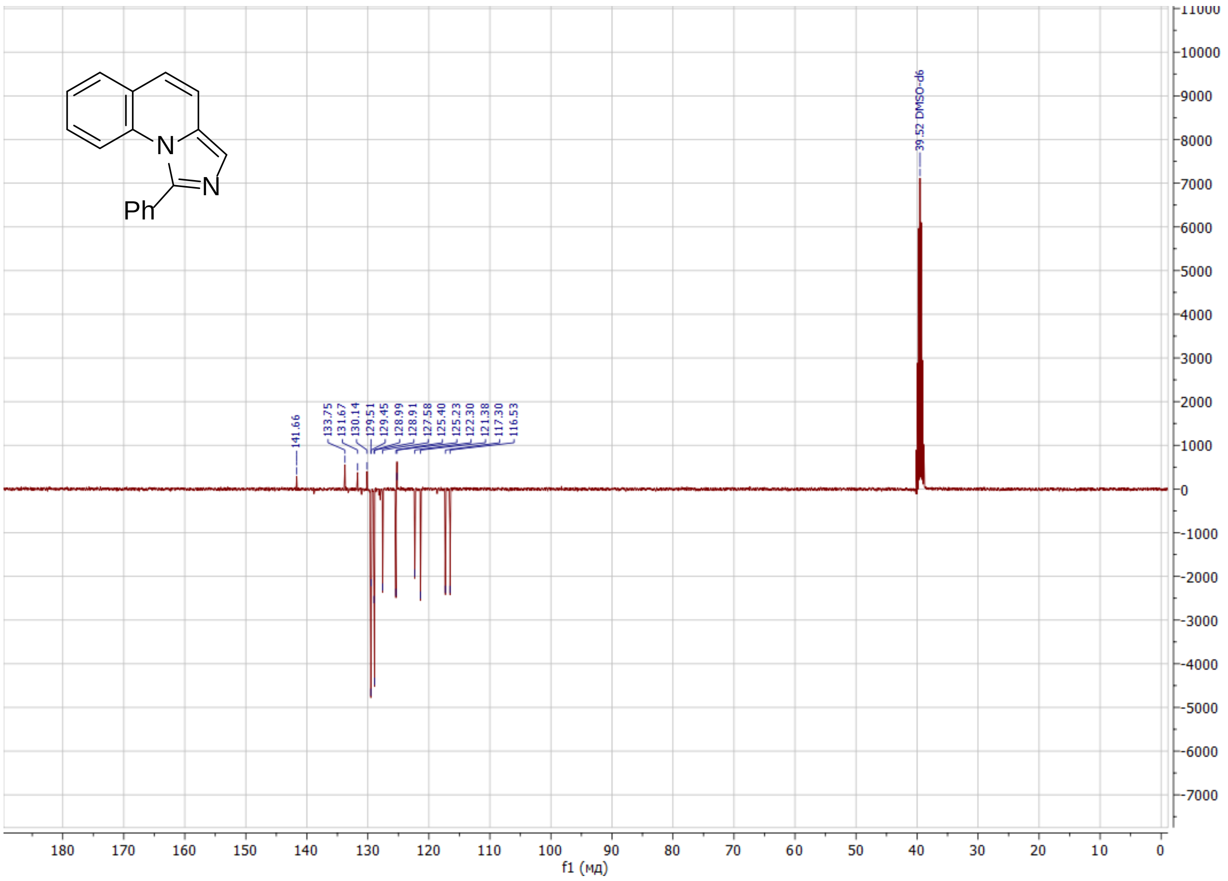

^{1}H (400 MHz) and ^{13}C (101 MHz) NMR spectra of **19be** in DMSO-d_6.

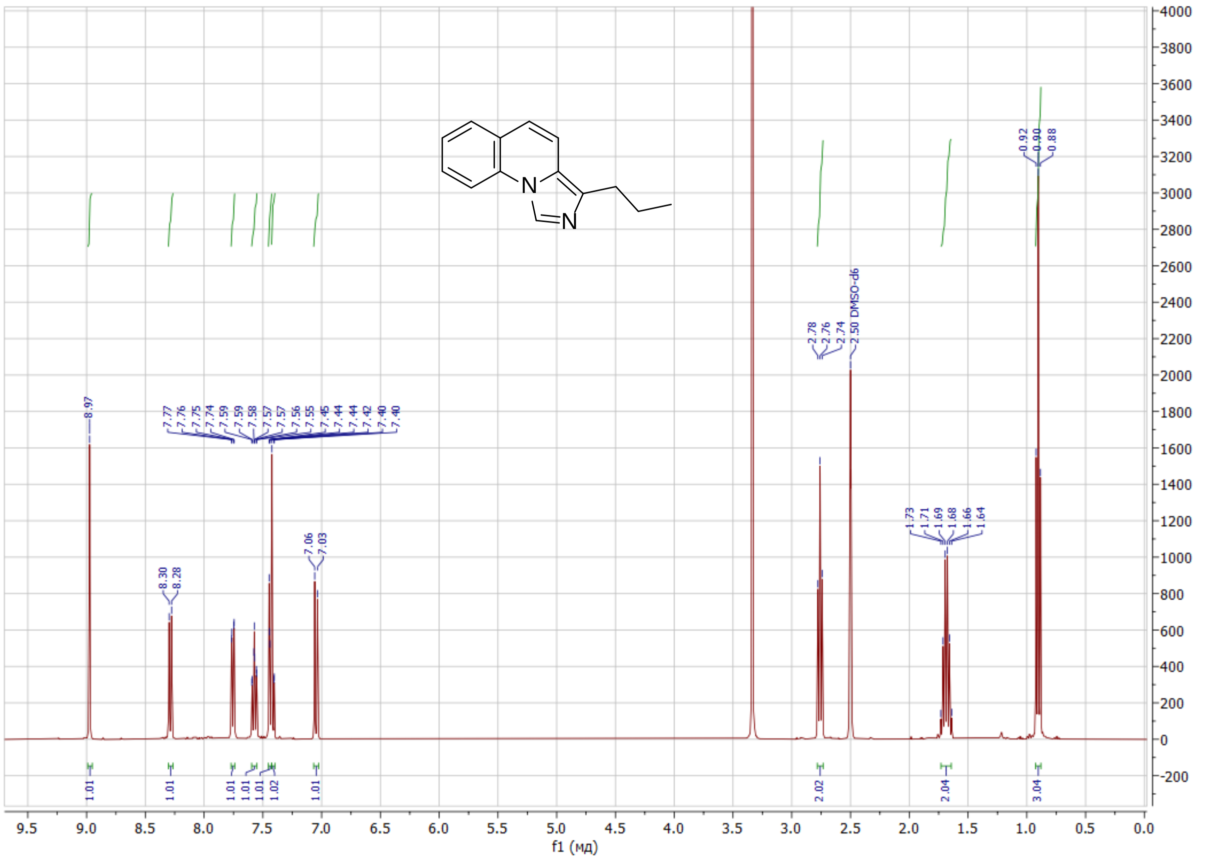

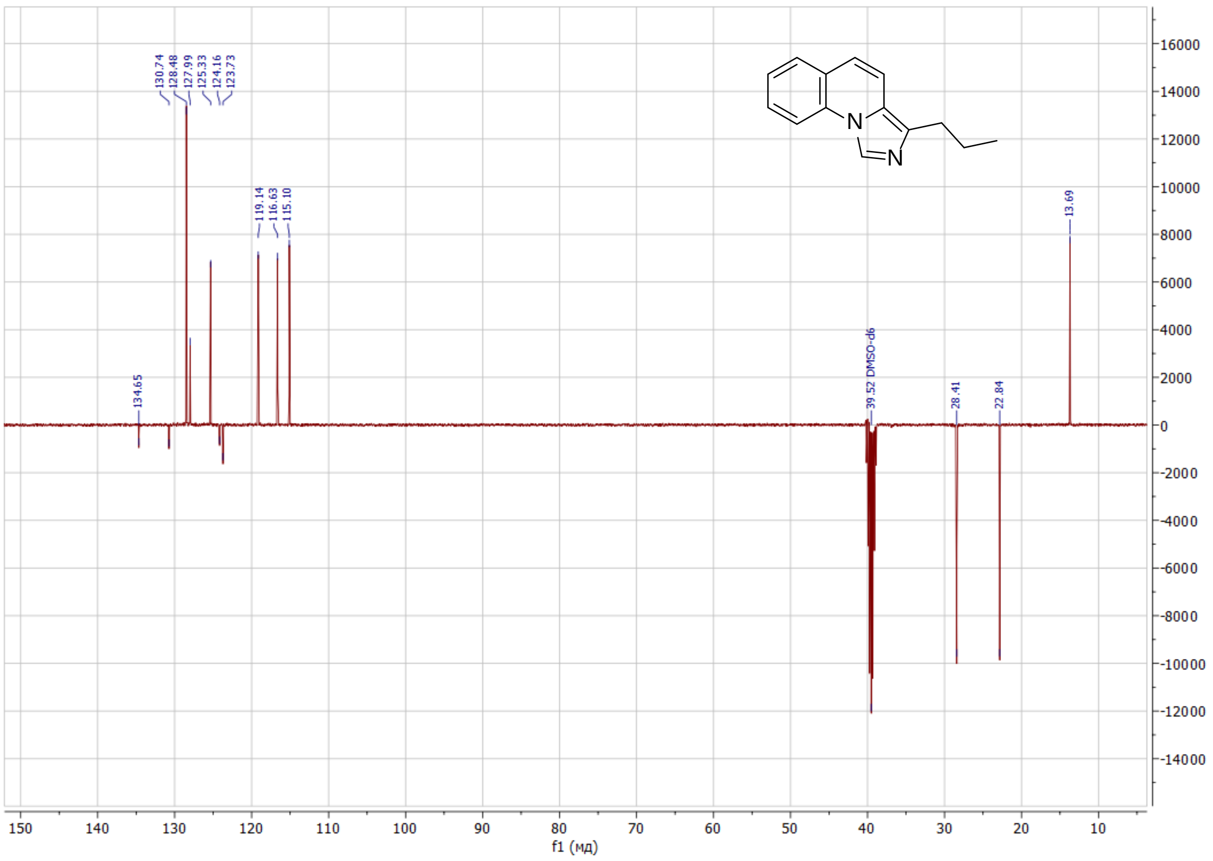

^{1}H (400 MHz) and ^{13}C (101 MHz) NMR spectra of **19bb** in $CDCl_3$.

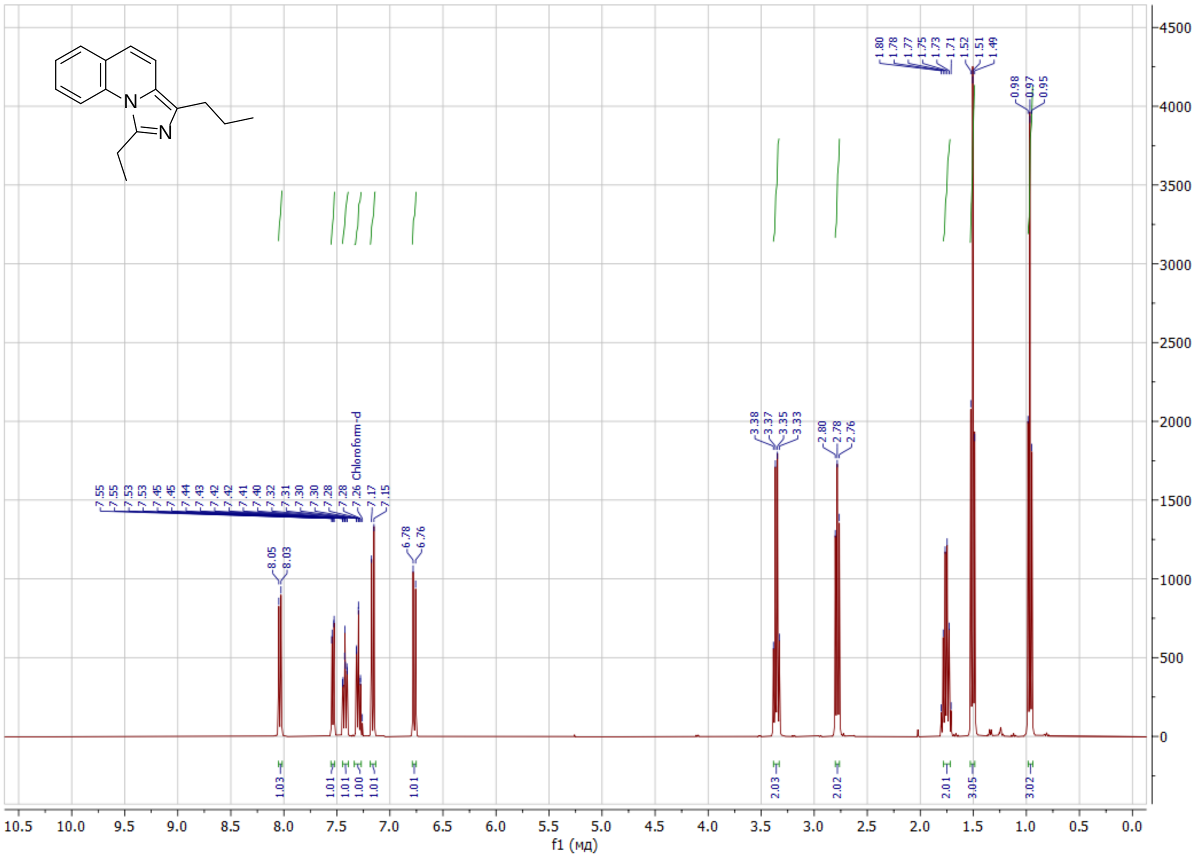

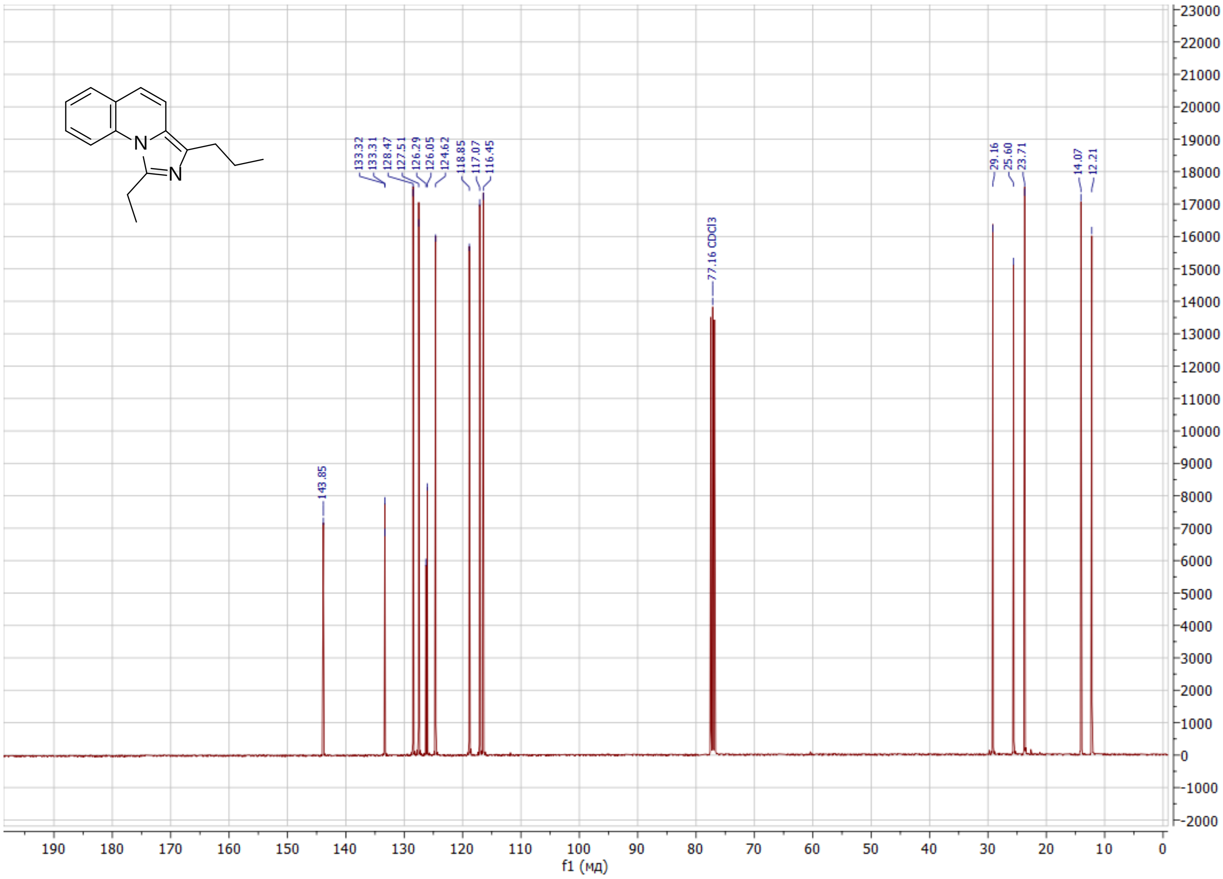

^{1}H (400 MHz) and ^{13}C (101 MHz) NMR spectra of **19bg** in $CDCl_3$.

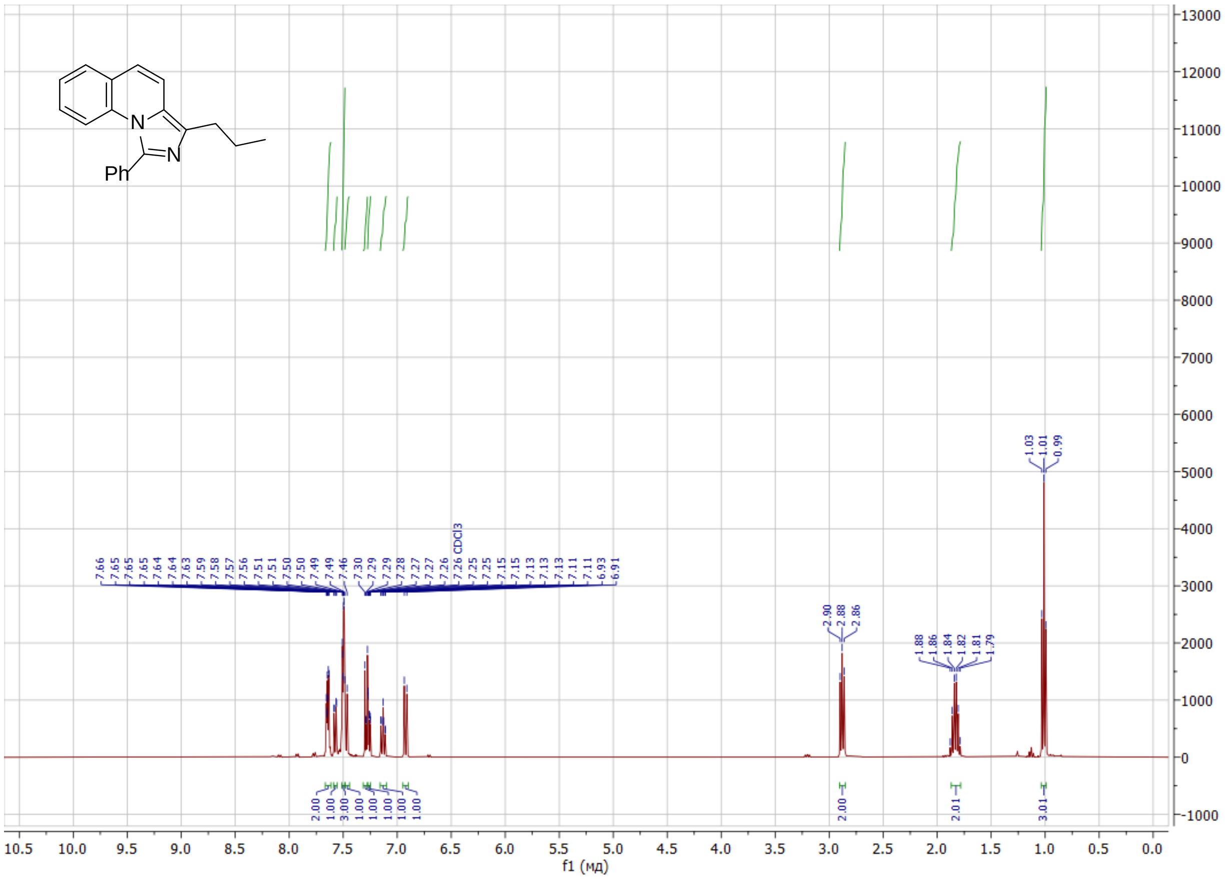

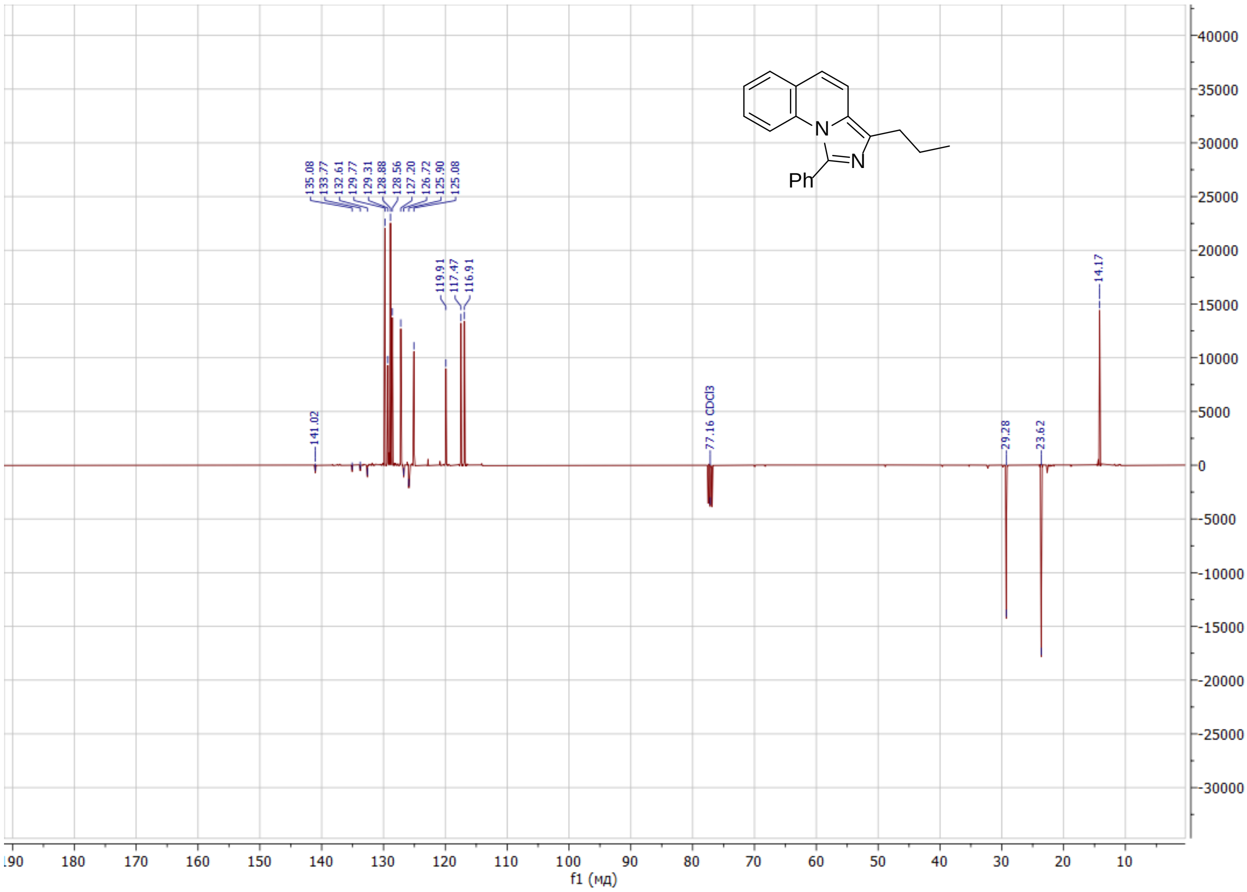

^{1}H (400 MHz) and ^{13}C (101 MHz) NMR spectra of **19ce** in $CDCl_3$.

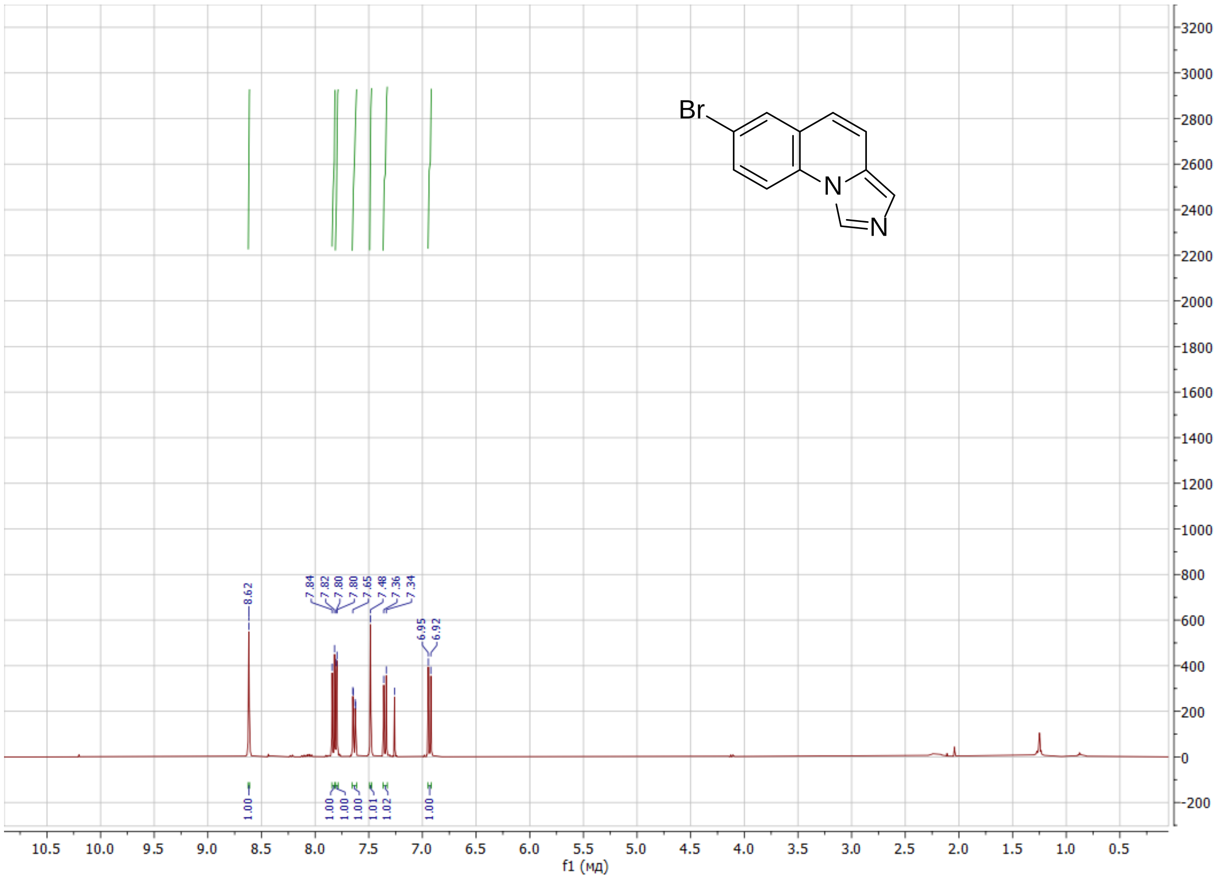

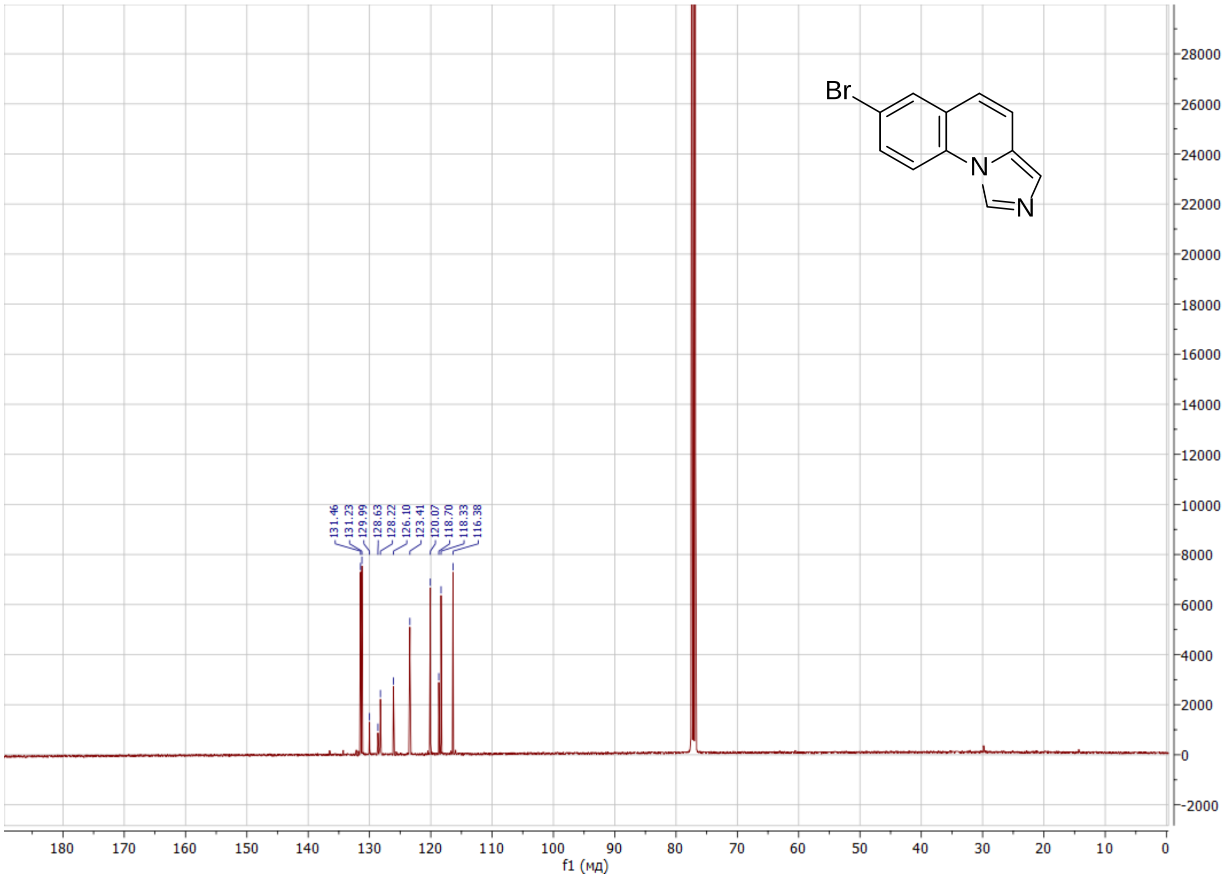

^{1}H (400 MHz) and ^{13}C (101 MHz) NMR spectra of **19de** DMSO-d_6.

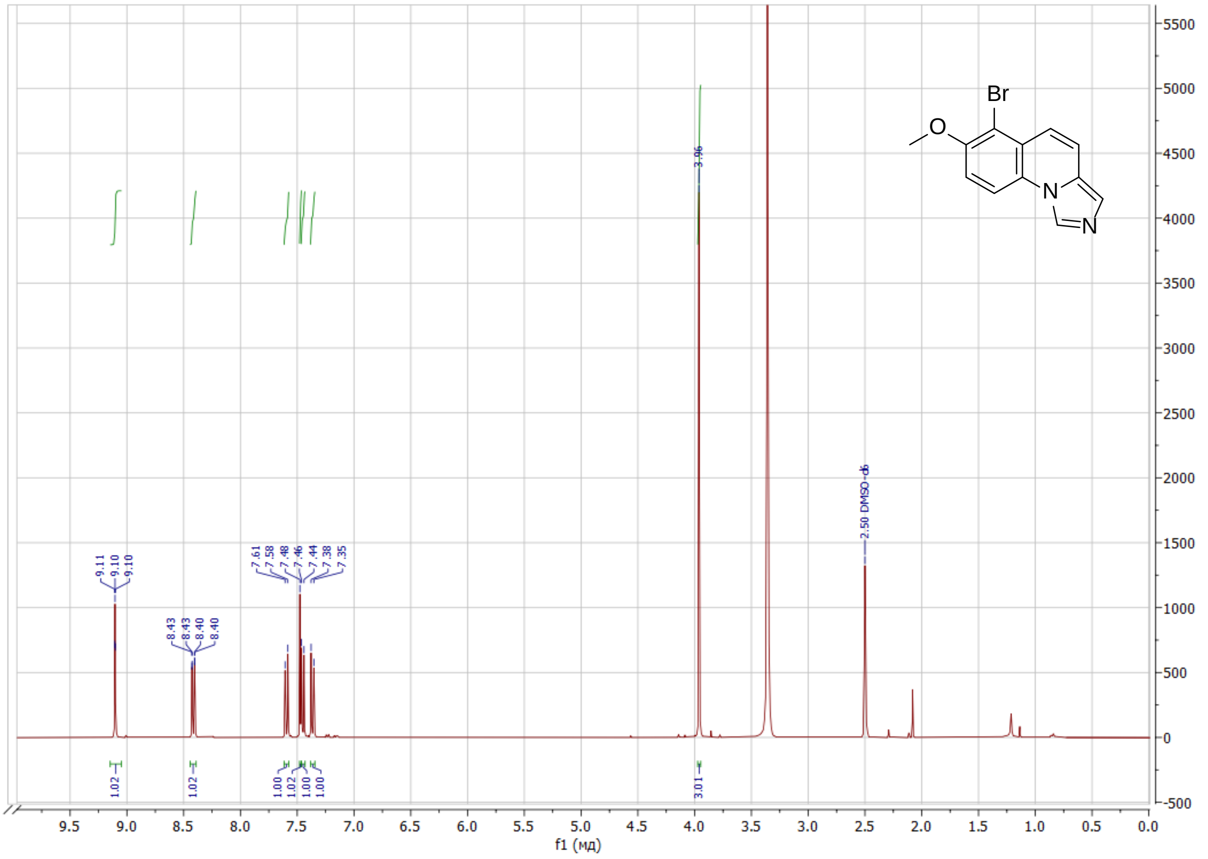

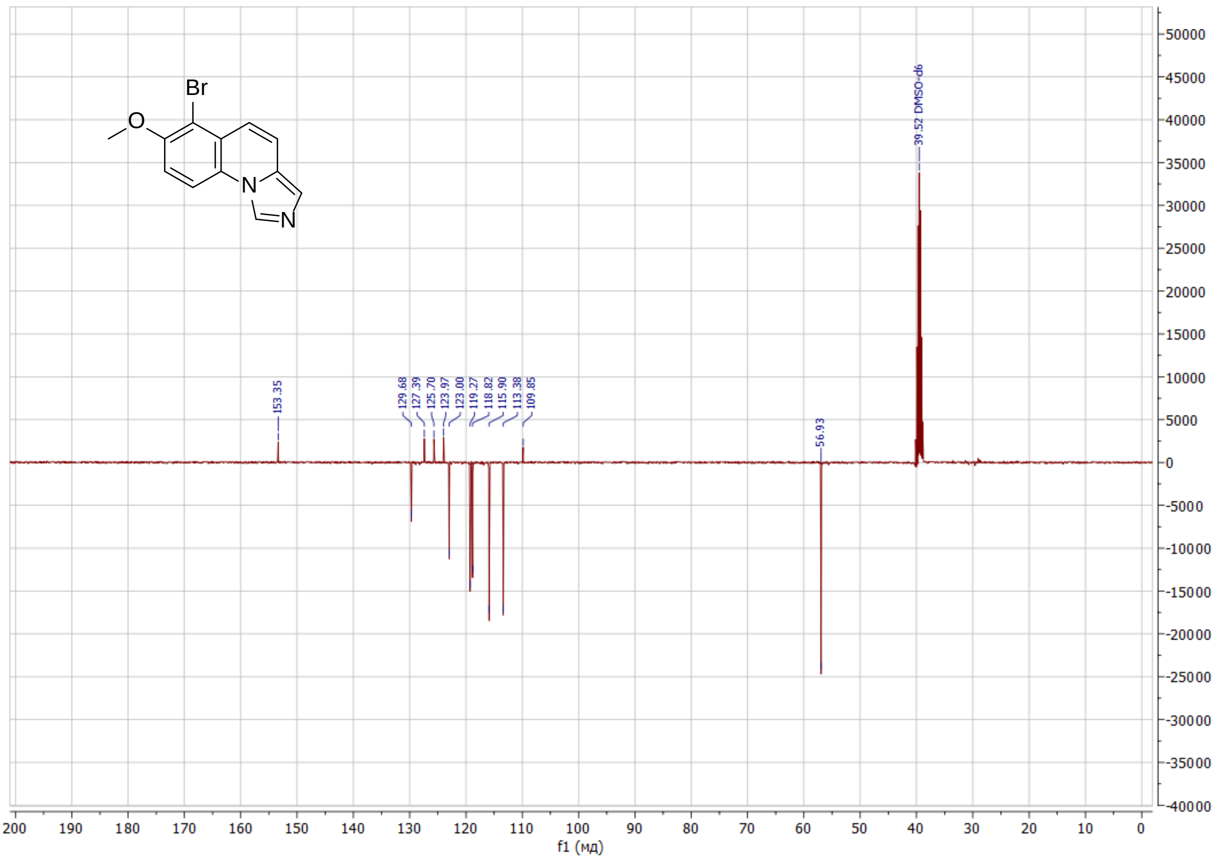

^{1}H (400 MHz) and ^{13}C (101 MHz) NMR spectra of **20** DMSO-d_6.

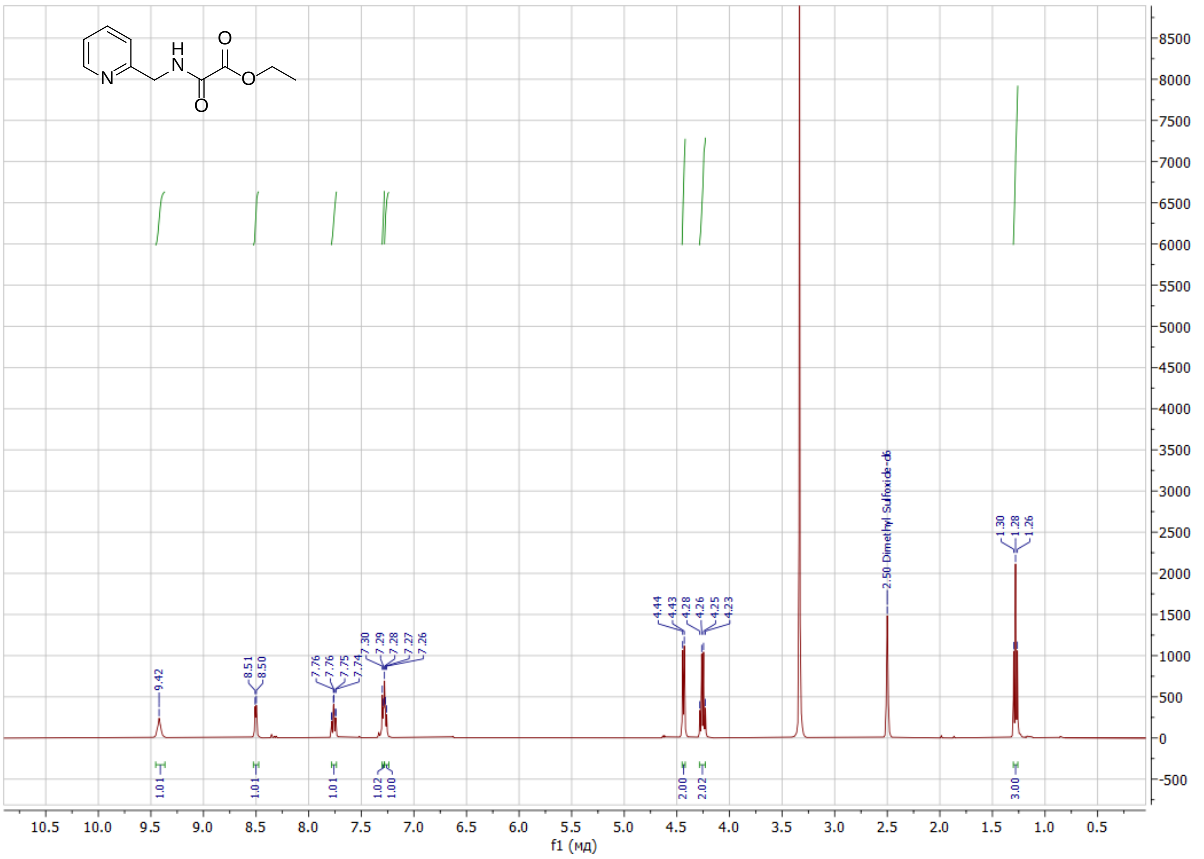

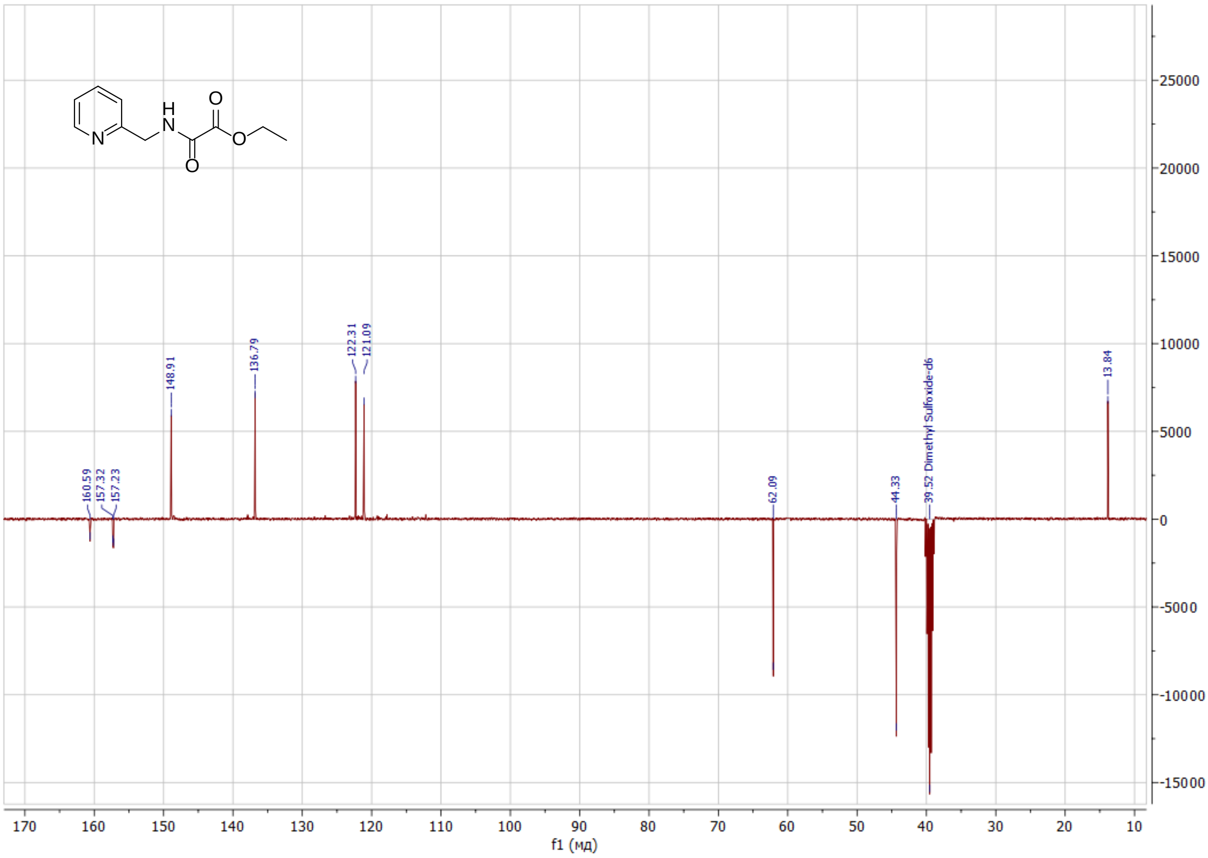

HRMS spectral charts

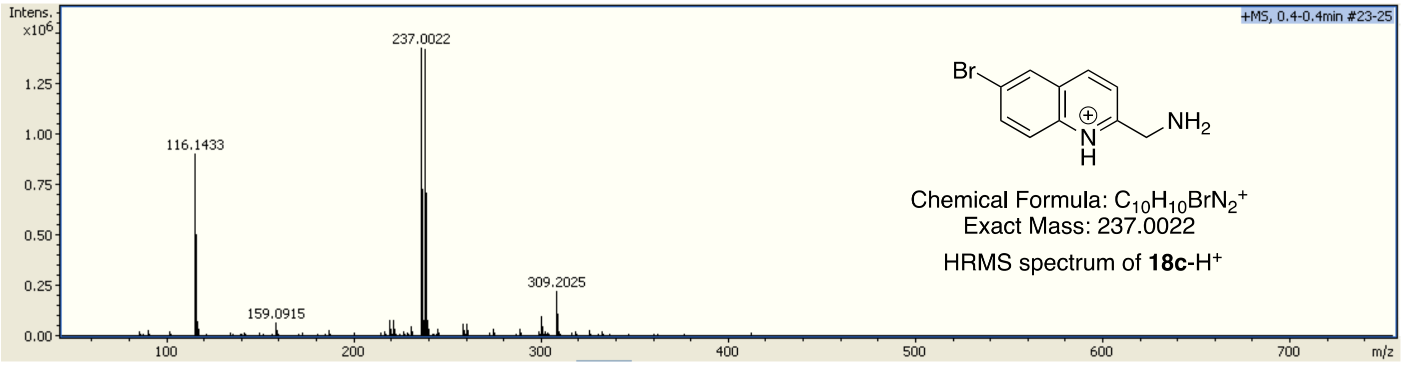

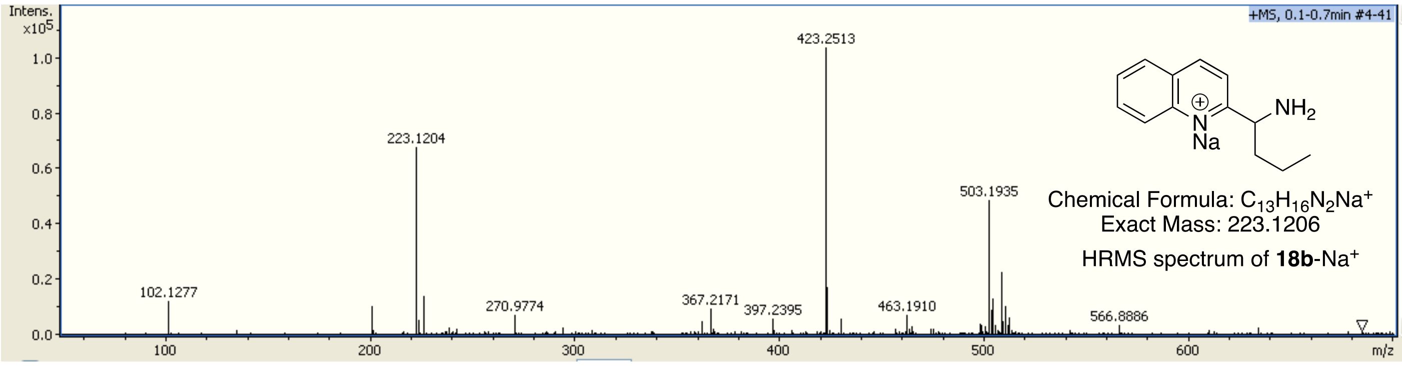

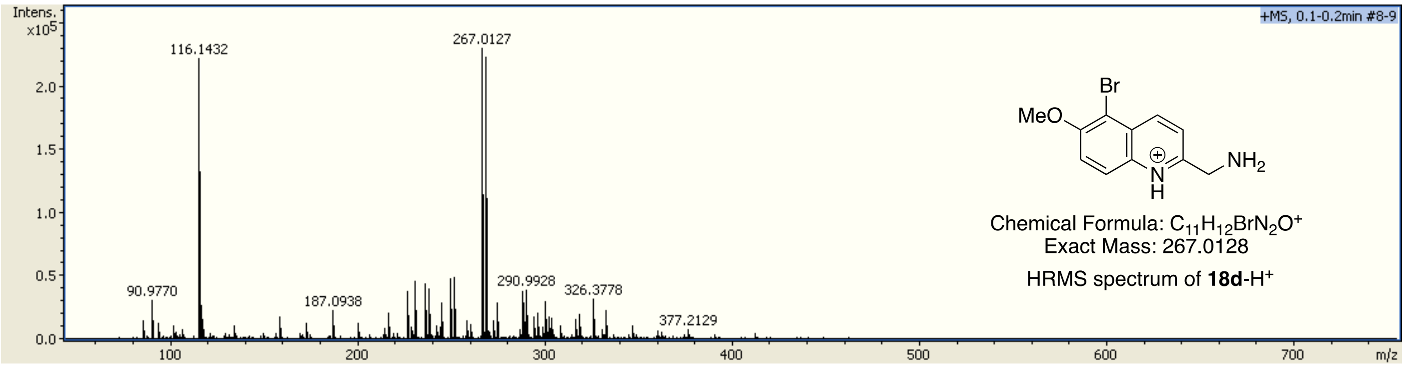

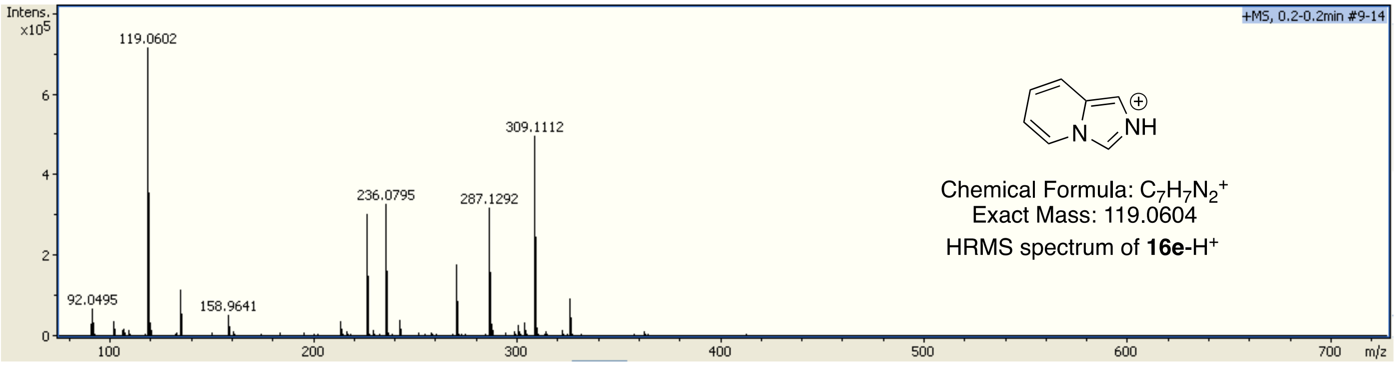

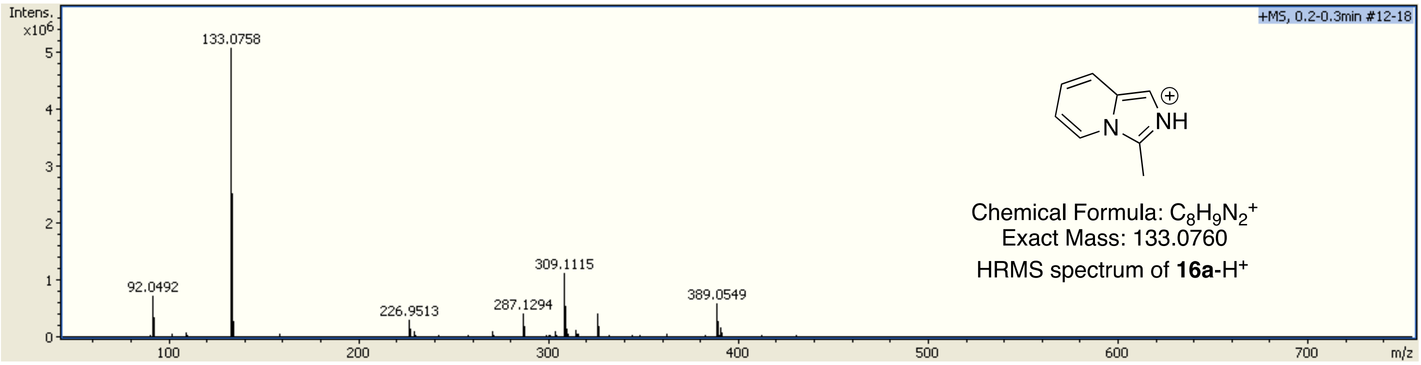
Intens.
x10^6
+MS, 0.2-0.3min #12-18
133.0758
92.0492
226.9513
287.1294
309.1115
389.0549
m/z
NH
N
Chemical Formula: $C_8H_9N_2^+$
Exact Mass: 133.0760
HRMS spectrum of **16a**-H+

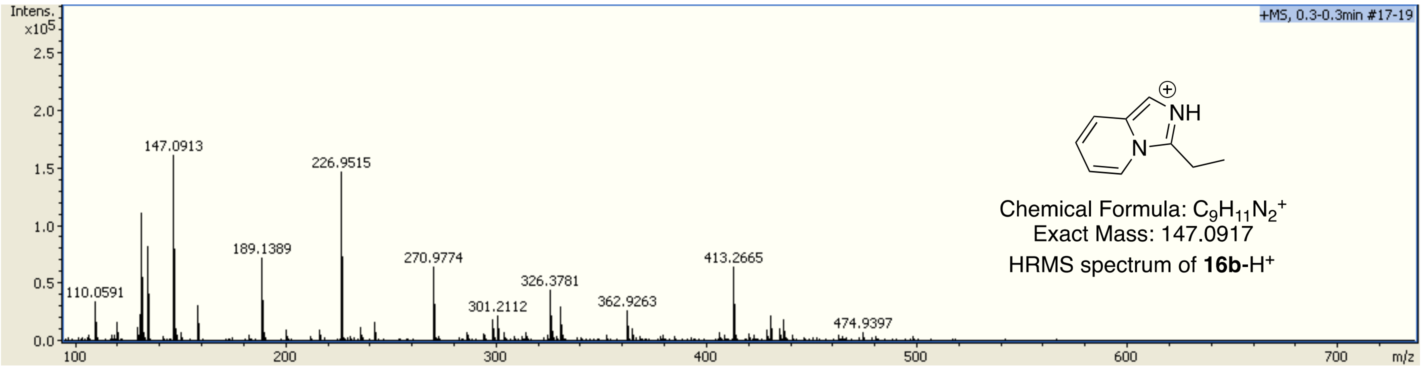
Intens.
x10^5
+MS, 0.3-0.3min #17-19
147.0913
226.9515
189.1389
270.9774
413.2665
110.0591
326.3781
301.2112
362.9263
474.9397
m/z
NH
N
Chemical Formula: $C_9H_{11}N_2^+$
Exact Mass: 147.0917
HRMS spectrum of **16b**-H+

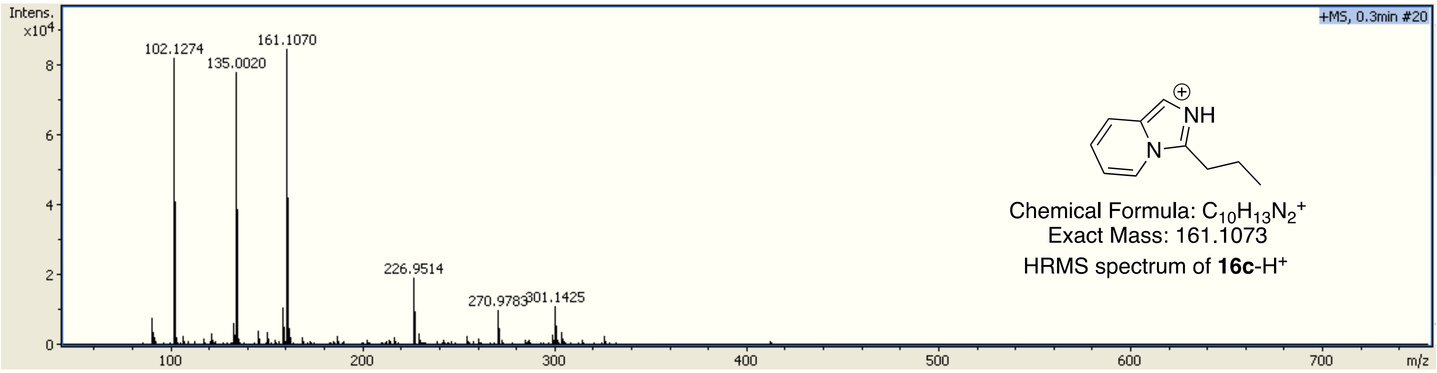
Intens.
x10^4
+MS, 0.3min #20
102.1274
161.1070
135.0020
226.9514
270.9783
301.1425
m/z
NH
N
Chemical Formula: $C_{10}H_{13}N_2^+$
Exact Mass: 161.1073
HRMS spectrum of **16c**-H+

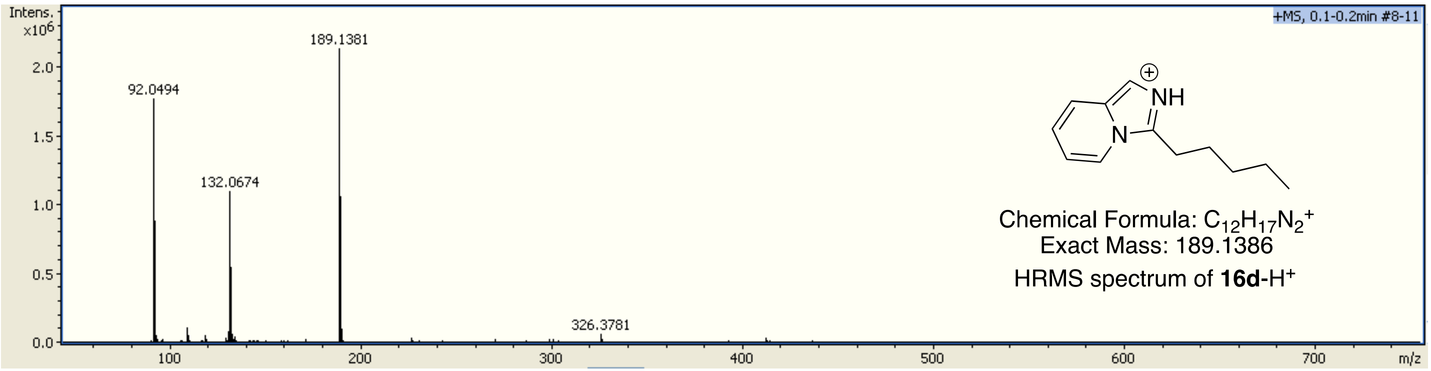
Intens.
x10^6
+MS, 0.1-0.2min #8-11
189.1381
92.0494
132.0674
326.3781
m/z
NH
N
Chemical Formula: $C_{12}H_{17}N_2^+$
Exact Mass: 189.1386
HRMS spectrum of **16d**-H+

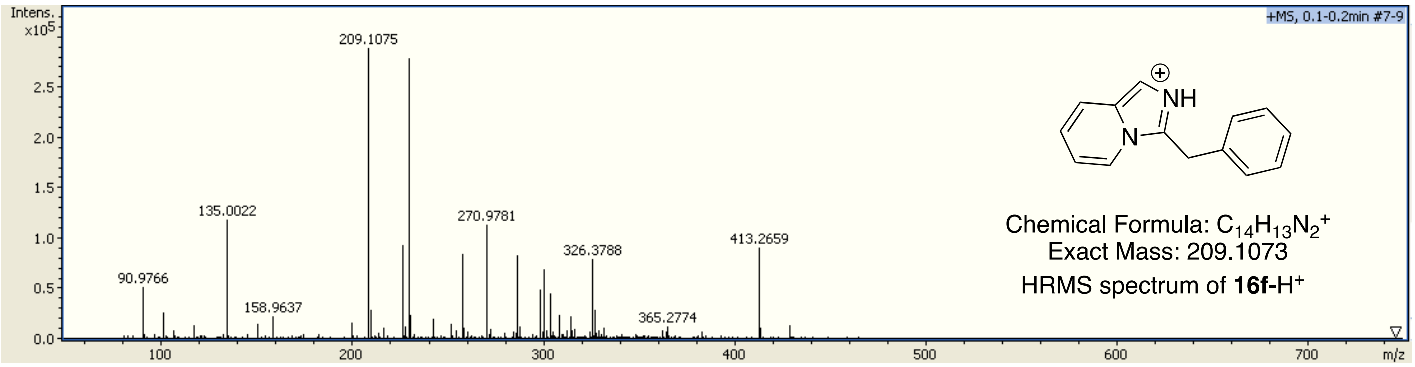
Intens.
x10^5
+MS, 0.1-0.2min #7-9
209.1075
135.0022
270.9781
326.3788
413.2659
90.9766
158.9637
365.2774
m/z
NH
N
Chemical Formula: $C_{14}H_{13}N_2^+$
Exact Mass: 209.1073
HRMS spectrum of **16f**-H+

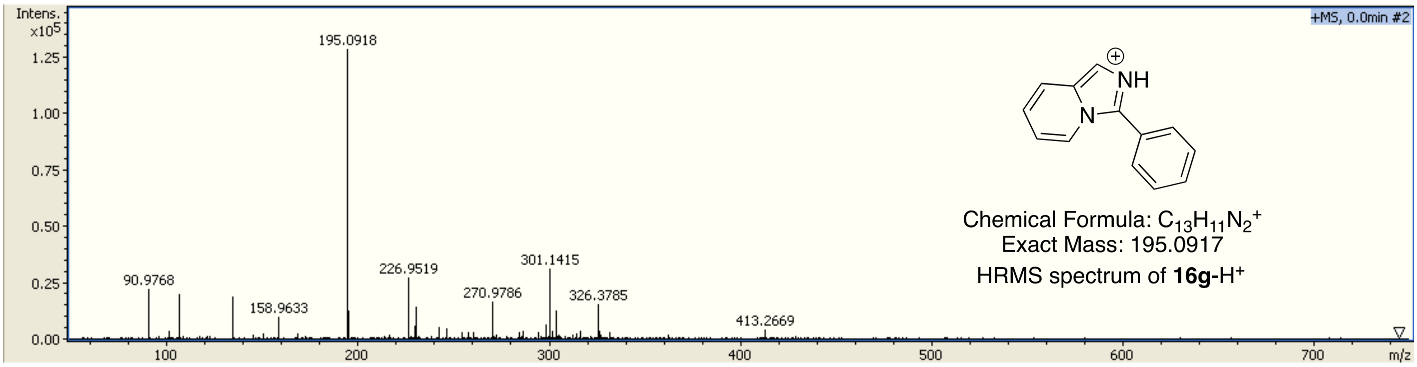
Intens.
x10^5
+MS, 0.0min #2
195.0918
90.9768
158.9633
226.9519
270.9786
301.1415
326.3785
413.2669
m/z
NH
N
Chemical Formula: $C_{13}H_{11}N_2^+$
Exact Mass: 195.0917
HRMS spectrum of **16g**-H+

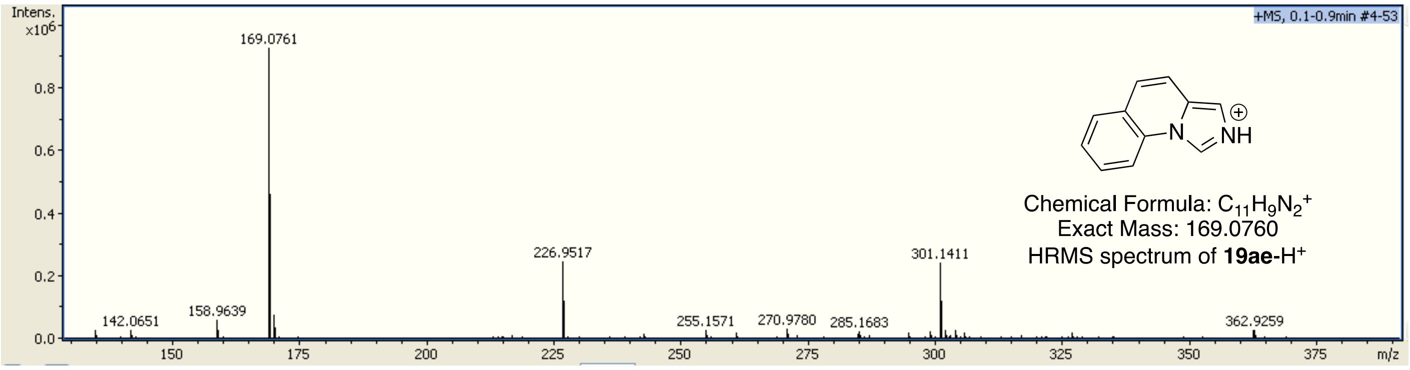
Intens.
x10^6
+MS, 0.1-0.9min #4-53
169.0761
142.0651
158.9639
226.9517
255.1571
270.9780
285.1683
301.1411
362.9259
m/z
N
NH
Chemical Formula: $C_{11}H_9N_2^+$
Exact Mass: 169.0760
HRMS spectrum of **19ae**-H+

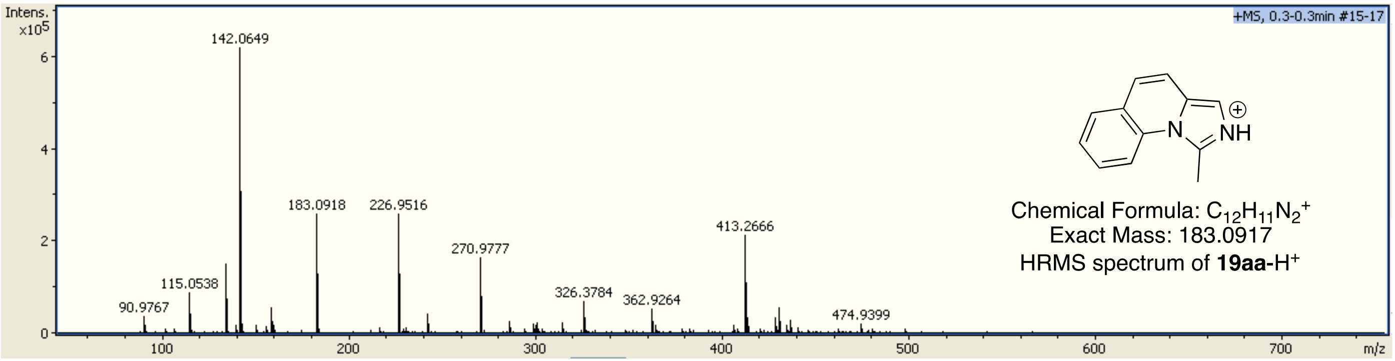
Intens.
x10^5
+MS, 0.3-0.3min #15-17
142.0649
183.0918
226.9516
270.9777
115.0538
90.9767
326.3784
362.9264
413.2666
474.9399
m/z
N
NH
Chemical Formula: $C_{12}H_{11}N_2^+$
Exact Mass: 183.0917
HRMS spectrum of **19aa**-H+

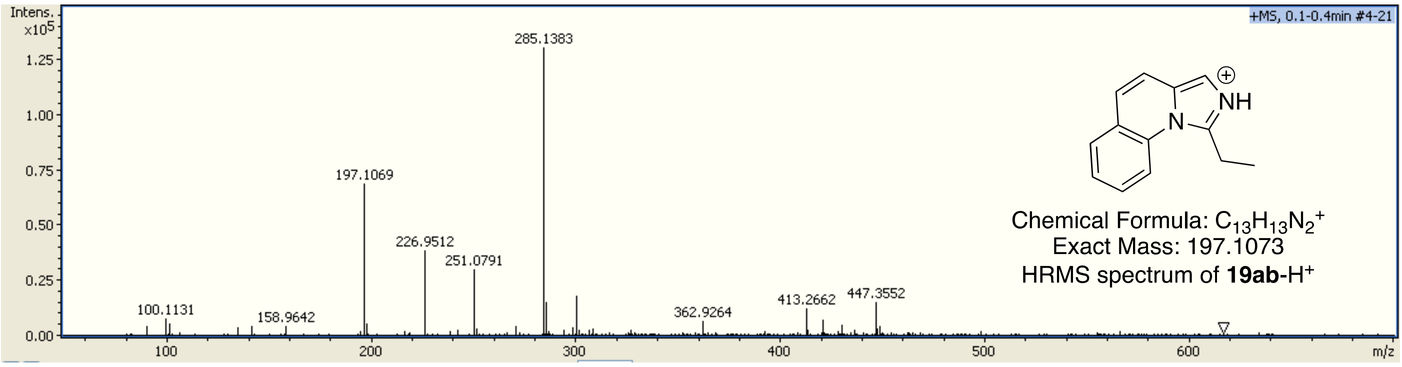
Intens.
x10^5
+MS, 0.1-0.4min #4-21
285.1383
197.1069
226.9512
251.0791
100.1131
158.9642
362.9264
413.2662
447.3552
m/z
NH
N
Chemical Formula: $C_{13}H_{13}N_2^+$
Exact Mass: 197.1073
HRMS spectrum of **19ab**-H+

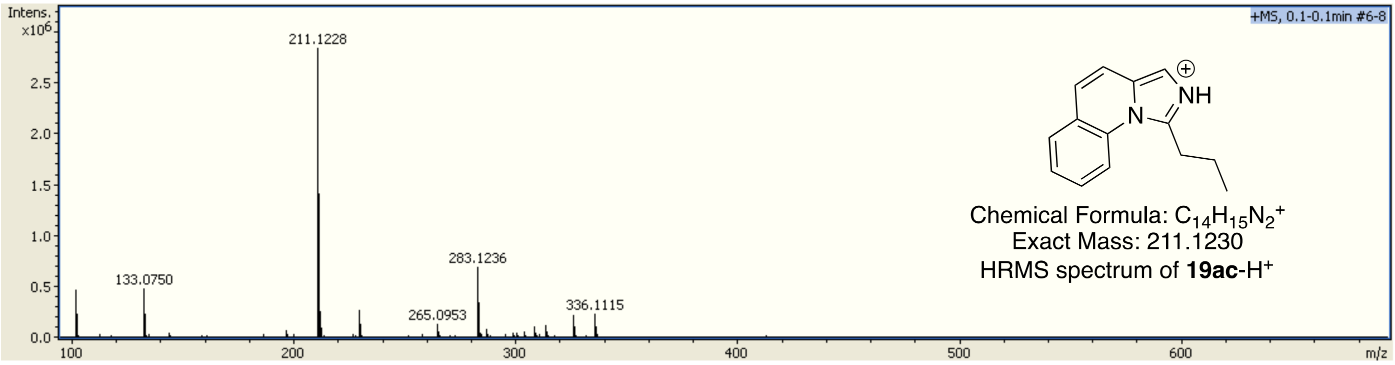
Intens.
x10^6
+MS, 0.1-0.1min #6-8
211.1228
133.0750
283.1236
265.0953
336.1115
m/z
NH
N
Chemical Formula: $C_{14}H_{15}N_2^+$
Exact Mass: 211.1230
HRMS spectrum of **19ac**-H+

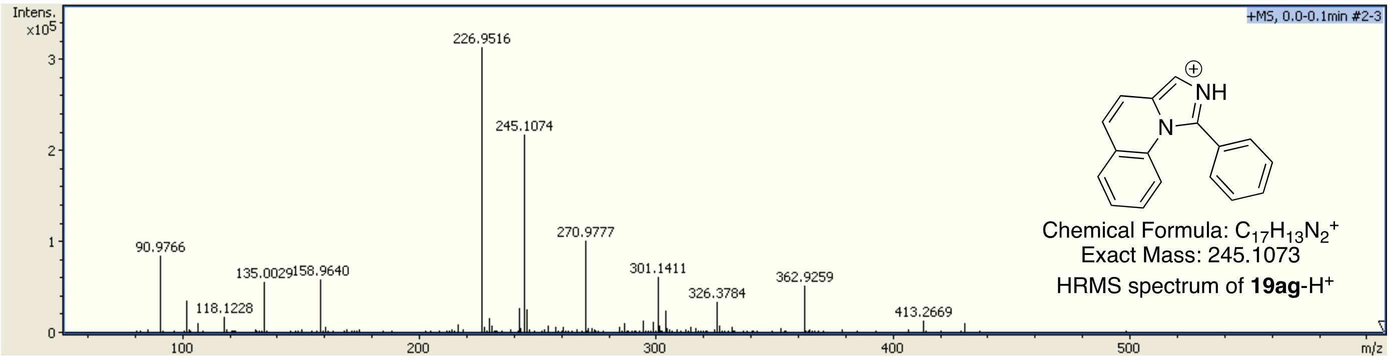
Intens.
x10^5
+MS, 0.0-0.1min #2-3
226.9516
245.1074
270.9777
90.9766
135.0029
158.9640
118.1228
301.1411
326.3784
362.9259
413.2669
m/z
NH
N
Chemical Formula: $C_{17}H_{13}N_2^+$
Exact Mass: 245.1073
HRMS spectrum of **19ag**-H+

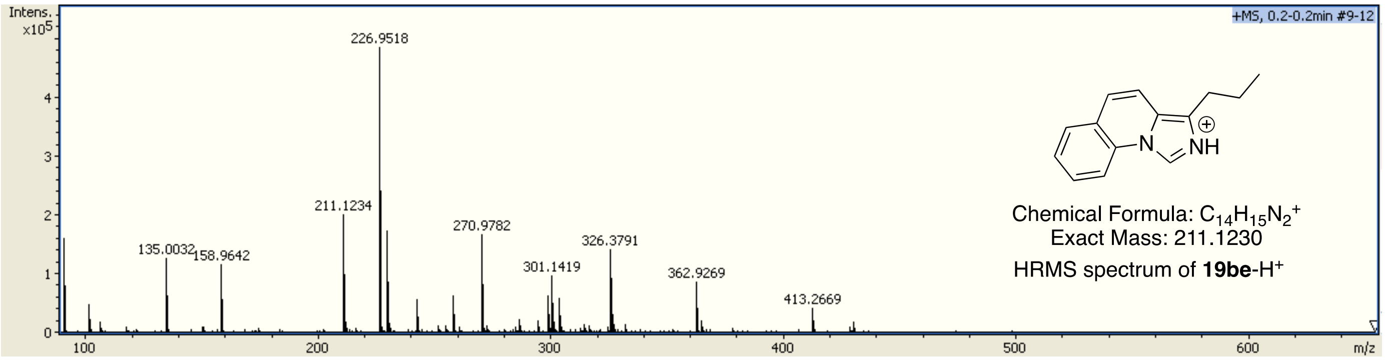
Intens.
x10^5
+MS, 0.2-0.2min #9-12
226.9518
211.1234
270.9782
135.0032
158.9642
326.3791
301.1419
362.9269
413.2669
m/z
N
NH
Chemical Formula: $C_{14}H_{15}N_2^+$
Exact Mass: 211.1230
HRMS spectrum of **19be**-H+

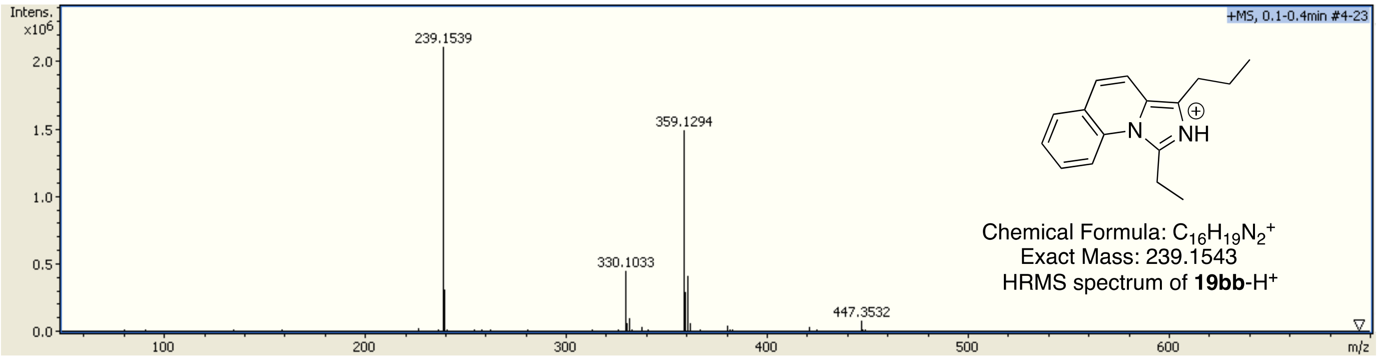
Intens.
x10^6
+MS, 0.1-0.4min #4-23
239.1539
359.1294
330.1033
447.3532
m/z
N
NH
Chemical Formula: $C_{16}H_{19}N_2^+$
Exact Mass: 239.1543
HRMS spectrum of **19bb**-H+

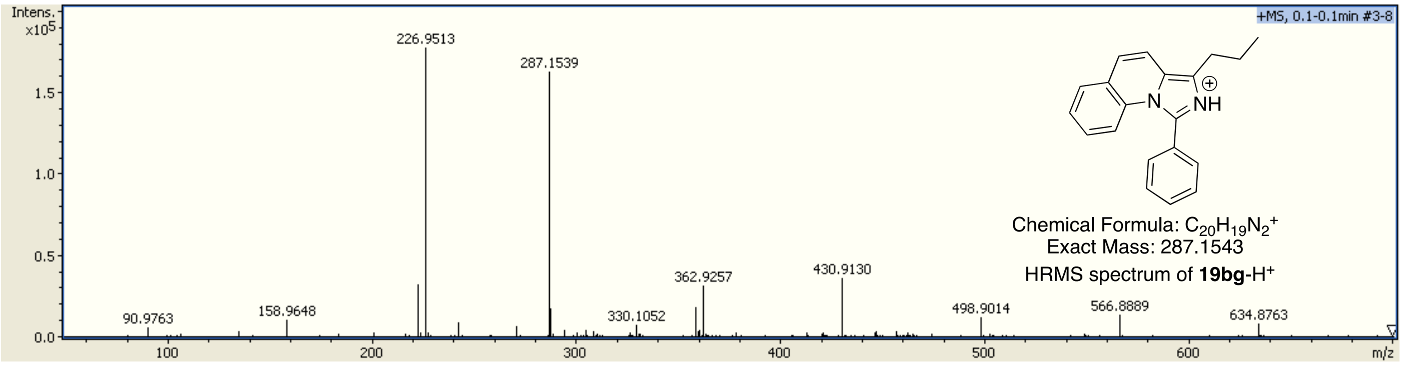
Intens.
x10^5
+MS, 0.1-0.1min #3-8
226.9513
287.1539
362.9257
430.9130
90.9763
158.9648
330.1052
498.9014
566.8889
634.8763
m/z
N
NH
Chemical Formula: $C_{20}H_{19}N_2^+$
Exact Mass: 287.1543
HRMS spectrum of **19bg**-H+

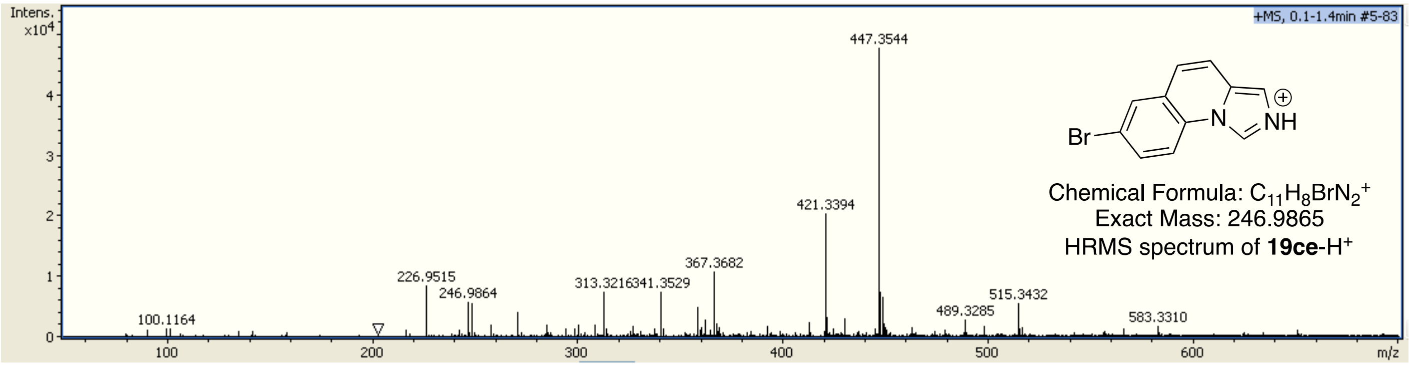
Intens.
x10^4
+MS, 0.1-1.4min #5-83
447.3544
421.3394
367.3682
226.9515
246.9864
313.3216
341.3529
100.1164
489.3285
515.3432
583.3310
m/z
Br
N
NH
Chemical Formula: $C_{11}H_8BrN_2^+$
Exact Mass: 246.9865
HRMS spectrum of **19ce**-H+

‘

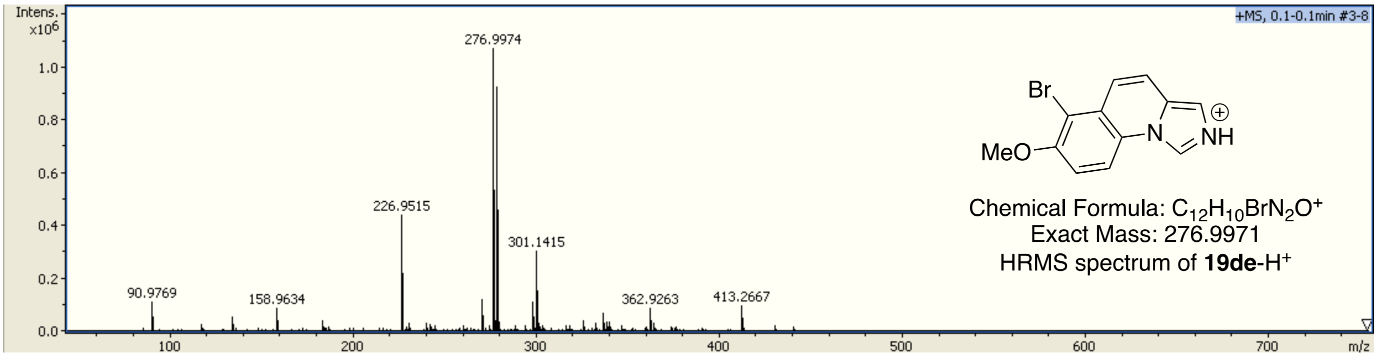

Intens.
x10^6
+MS, 0.1-0.1min #3-8
276.9974
226.9515
301.1415
90.9769
158.9634
362.9263
413.2667
Br
MeO
N
NH
Chemical Formula: $C_{12}H_{10}BrN_2O^+$
Exact Mass: 276.9971
HRMS spectrum of **19de**-H+
m/z

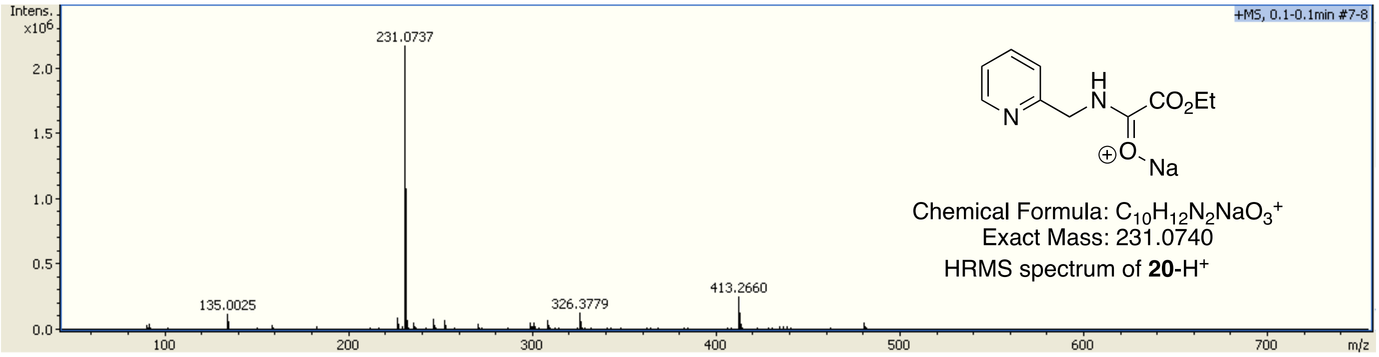

Intens.
x10^6
+MS, 0.1-0.1min #7-8
231.0737
135.0025
326.3779
413.2660
H
N
N
CO_2Et
O
Na
Chemical Formula: $C_{10}H_{12}N_2NaO_3^+$
Exact Mass: 231.0740
HRMS spectrum of **20**-H+
m/z
